# Identification of immunity-related lncRNAs and construction of a ceRNA network of potential prognostic biomarkers in acute myeloid leukemia

**DOI:** 10.3389/fgene.2023.1203345

**Published:** 2023-06-14

**Authors:** Jia Xue, Haoran Chen, Jinqi Lu, Haojun Zhang, Jie Geng, Peifeng He, Xuechun Lu

**Affiliations:** ^1^ School of Basic Medical Sciences, Shanxi Medical University, Taiyuan, Shanxi, China; ^2^ School of Management, Shanxi Medical University, Taiyuan, Shanxi, China; ^3^ Department of Computer Science, Boston University, Boston, MA, United States; ^4^ Shanxi Key Laboratory of Big Data for Clinical Decision Research, Taiyuan, Shanxi, China; ^5^Department of Hematology, The Second Medical Center and National Clinical Research Center for Geriatric Diseases, Chinese PLA General Hospital, Beijing, China

**Keywords:** acute myeloid leukemia, competing endogenous RNA, prognostic model, immunity-related biomarker, bioinformatics

## Abstract

**Objective:** Using bioinformatics analyses, this study aimed to identify lncRNAs related to the immune status of acute myeloid leukemia (AML) patients and ascertain the potential impact in immunity-related competing endogenous RNA (ceRNA) networks on AML prognosis.

**Methods:** AML-related RNA-seq FPKM data, AML-related miRNA expression microarray data, and gene sets associated with immunity-related pathways were, respectively, obtained from the TCGA, GEO, and ImmReg databases. An immunity-related ceRNA network was then constructed according to the predicted interactions between AML-related mRNAs, lncRNAs, and miRNAs. After performing LASSO and multivariate Cox regression analyses, lncRNAs in the ceRNA network were used to establish an AML prognostic model. According to mutual regulatory relationships and consistent trends of expression among candidate ceRNAs, two ceRNA subnetworks related to the AML prognostic model were determined. Finally, the correlation between the expression levels of mRNAs, lncRNAs, and miRNAs in each ceRNA subnetwork and immune cell infiltration (assessed by combining the ESTIMATE and CIBERSORT methods and ssGSEA) was analyzed.

**Results:** A total of 424 immunity-related differentially expressed (IR-DE) mRNAs (IR-DEmRNAs), 191 IR-DElncRNAs, and 69 IR-DEmiRNAs were obtained, and a ceRNA network of 20 IR-DElncRNAs, 6 IR-DEmRNAs, and 3 IR-DEmiRNAs was established. Univariate Cox regression analysis was conducted on 20 IR-DElncRNAs, and 7 of these were identified to be significantly correlated with the overall survival (OS) time in AML patients. Then, two IR-DElncRNAs (MEG3 and HCP5) were screened as independent OS-related factors by LASSO and multivariable Cox regression analyses, and a prognostic model was constructed to evaluate the survival risk in AML patients. Survival analyses indicated that the OS of patients was often poor in the high-risk group. Additionally, from this model, two ceRNA regulatory pathways, namely, MEG3/miR-125a-5p/SEMA4C and HCP5/miR-125b-5p/IL6R, which were potentially involved in the immune regulation of AML prognosis were identified.

**Conclusion:** lncRNAs HCP5 and MEG3 may act as key ceRNAs in the pathogenesis in AML by regulating immune cell representation as part of the regulatory lncRNA-miRNA-mRNA axes. The candidate mRNAs, lncRNAs, and miRNAs included in the ceRNA network identified here may serve as useful prognostic biomarkers and immunotherapeutic targets for AML.

## 1 Introduction

Acute myeloid leukemia (AML) is a blood disease that is malignant and genetically heterogeneous. In 2020, it accounted for 3.1% of new deaths and 2.5% of new cancer cases around the whole world, with a median age of 68 years at diagnosis (Yamamoto and Goodman, 2008; Sung et al., 2021). In younger adults with *de novo* AML, the 5-year overall survival rate range was 40%–50% (Appelbaum et al., 2006; Korn and Méndez-Ferrer, 2017; Stein et al., 2017). With continuous advances in drug research and development and improved treatment choices for AML, the prognosis of young patients has improved further in recent decades. However, the prognosis of adult and especially elderly patients is poor as before, with a 5-year expected survival rate of only 20%–25% (Shah et al., 2013; Shallis et al., 2019). Therefore, new diagnostic and prognostic biomarkers are urgently required to further enhance AML patients’ prognosis.

Long-strand non-coding RNAs (lncRNAs) and microRNAs (miRNAs) are both non-coding RNAs, the former with over 200 nucleotides in length that plays a crucial role in chromatin and genome remodeling, RNA stability, and transcriptional regulation, and the latter with about 22 nucleotides are single-stranded RNAs. They inhibit protein translation by binding to the 3′-untranslated region of mRNAs, thus silencing the targeted mRNAs at the post-transcriptional or translational levels (Gebert and MacRae, 2019; Treiber et al., 2019). Abnormal expression of lncRNAs and miRNAs prove to be pivotal in the occurrence, progression, metastasis, and prognosis of cancer. Mounting evidence shows that lncRNAs and miRNAs can act as new biomarkers for treating AML patients (Chen et al., 2016; Wallace and O’Connell, 2017; Chen et al., 2019). Both lncRNAs and miRNAs can be used as competing endogenous RNAs (ceRNAs), conforming complex post-transcriptional regulatory networks through mutual interactions with each other and with mRNA species. Through competitive binding, lncRNAs can regulate and control the expression levels of miRNA target genes by acting as miRNA sponges, thus establishing ceRNA networks defined by specific lncRNA-miRNA-mRNA interactions (Sun et al., 2018; Wang et al., 2019). Several research studies have shown that dysregulated lncRNA expression affects immune cell activation correlative gene expression, which significantly influences tumor microenvironment through regulating immune cell infiltration (Atianand et al., 2017; Chen et al., 2017).

In the last few years, the analysis of infiltrating immune cells has pushed forward the development of immune checkpoint inhibitors, which has proved highly valuable in the treatment of solid tumor types (Tawbi et al., 2018). However, limited research devoted to systematic analysis of the tumor microenvironment in AML has precluded the development of robust prognostic models based on immune-related genes. Therefore, the aims of this study are 1) to identify key regulatory ceRNA networks involved in immune cell activity influencing the pathogenesis of AML; 2) to examine the role of lncRNAs in AML-related immune cell infiltration; and 3) to build a model that can reliably predict the prognosis of AML based on immunity-related lncRNAs. To this end, we applied bioinformatics methods to identify immunity-related differentially expressed (IR-DE) lncRNAs (IR-DElncRNAs), IR-DEmiRNAs, and IR-DEmRNAs in AML patient samples. The prognostic model presented here provides novel insights in the study of AML and may be a valuable aid in clinical decision-making.

## 2 Materials and methods

### 2.1 Data acquisition

Clinical and RNA-seq (FPKM format) data from 150 AML patients were downloaded from the TCGA database (TCGA-LAML; https://portal.gdc.cancel.gov/). Samples without survival information were filtered out, and 131 samples with corresponding prognosis information were finally obtained. The samples were randomized into training sets (*n* = 67) and validation sets (*n* = 64) for further analyses. In order to compare, we downloaded the RNA-seq data from GTEx (https://gtexportal.org/home/), which included 70 healthy controls. The GSE142699 data set, containing microarray-based whole-blood miRNA gene expression data (platform ID: GPL26945) from 24 AML patients and 24 healthy donors, was retrieved from GEO (https://www.ncbi.nlm.nih.gov/geo/).

### 2.2 Analysis of differentially expressed genes

We downloaded the reference human genome annotation file set (the GTF) from Ensembl (https://asia.ensembl.org) and ran Perl scripts to perform ID conversion on the GTEx gene expression data and convert Ensembl transcript IDs into gene names for subsequent analyses. In RStudio (R version 4.1.1), we screened for differentially expressed mRNAs (DEmRNAs), lncRNAs (DElncRNAs), and miRNAs (DEmiRNAs) by using the “limma” package in R, defined by |log2-fold change (FC)|> 1 and *p* < 0.05, in AML samples relative to healthy controls.

### 2.3 Identification of immunity-related differentially expressed RNAs

The list of immunity-related lncRNAs (IRlncRNAs) and miRNAs (IRmiRNAs) in TCGA-LAML was extracted from ImmReg (the regulon atlas of immune-related pathways across cancer types, http://bio-bigdata.hrbmu.edu.cn/ImmReg/index.Jsp) (Jiang et al., 2021). Then, we further downloaded a list of recognized IRmRNAs from ImmPort (Immunology Database and Analysis Portal, https://www.immport.org/shared/genelists) to retrieve a set of AML-related IR-DElncRNAs, IR-DEmiRNAs, and IR-DEmRNAs via intersection analysis with the previously identified DElncRNAs, DEmiRNAs, and DEmRNAs.

### 2.4 Construction of AML-related ceRNA network

According to the identified lncRNA-miRNA-mRNA interactions, we constructed an AML-related ceRNA network. We first predicted the miRNAs interacting with IR-DElncRNAs in the miRcode (http://www.mircode.org) and intersected miRNAs with IR-DEmiRNAs to retrieve candidate IR-DEmiRNAs. Three databases, namely, TargetScan (https://www.targetscan.org), miRDB (https://mirdb.org) and miRTarBase (https://miRTarBase.cuhk.edu.cn), were accessed to predict miRNA interactions with target mRNAs, and we finally intersected the identified IR-DEmRNAs to obtain the shared mRNAs. Then, the AML-related ceRNA network was constructed by Cytoscape (version 3.8.2).

### 2.5 Construction of IR-DElncRNA–based prognostic model for AML

The IR-DElncRNAs included in the ceRNA network were first analyzed by univariate Cox regression analysis on the TCGA training data set to select those related to the overall survival (OS) of AML patients. Then, the least absolute shrinkage and selection operator (LASSO) and multivariate Cox regression analyses were applied to identify the factors that were independently related to the OS and build a clinical predictive model to evaluate the prognostic risk of AML patients. Subsequently, we assigned a risk score to each AML patient based on the formula that we created for OS. The AML patients were grouped on the basis of the median risk score, and then they were classified into low- and high-risk groups. The survival status and total survival period of patients in each group were compared using the “survival” and “timeROC” packages to generate the receiver operating characteristic (ROC) curves for 1-, 3-, and 5-year OS. The principal component analysis (PCA) was applied to test OS differences between the groups. Finally, based on mutual regulatory relationships and consistent trends of the change in the expression levels among ceRNAs, a ceRNA subnetwork related to the AML prognostic model was determined.

### 2.6 Analysis of the correlation between AML prognostic model and AML microenvironment

Stromal cells and immune cells constitute two main classes of non-tumor cells with diagnostic and prognostic values that coexist in the tumor microenvironment. The Estimation of STromal and Immune cells in MAlignant Tumors using Expression data (ESTIMATE) algorithm allows predicting the abundance of infiltrating immune cells (Yoshihara et al., 2013). According to the gene expression of 11,057 samples in 33 tumors and based on the ratio of stromal to immune cells in each tumor sample, tumor purity was calculated by using the “limma” and “ESTIMATE” packages after deleting normal samples. Then, we used the Spearman’s test to analyze the correlation between the risk score of the prognostic model and tumor microenvironment scores and draw the correlation distribution by using the “ggplot2,” “ggExtra,” and “ggpubr” packages in R.

### 2.7 Analysis of correlation between AML prognostic model and immune cell characteristics

Immune cell infiltration analysis was performed on the MSigDB database (http://www.gsea-msigdb.org/gsea/msigdb/) using the single-sample gene set enrichment analysis (ssGSEA), which allows calculating the scores of 29 immune signatures corresponding to 16 immune cell types and 13 immune functions. After retrieving the relevant gene sets, we performed ssGSEA on the transformed expression matrix through the “GSVA” package. According to the results of the ssGSEA, we calculated the correlation between the immune cells and functions by using the “corrplot” package, with results visualized as a heatmap. In addition, to assess score differences of immune cells and functions between the two patient groups, the sample group and ssGSEA expression matrix results were integrated, and the rank-sum test was performed for data visualization by using the “ggpubr” package.

To analyze the presence of 22 human immune cell types in tumor samples, we used the Cell-type Identification By Estimating Relative Subsets Of RNA Transcripts (CIBERSORT, http://cibersort.stanford.edu). Then, we analyzed RNA-seq FPKM data from TCGA-LAML using the “CIBERSORT” package.

### 2.8 Enrichment analysis of lncRNAs associated with AML prognostic model

To determine relevant functions and assess signaling pathway involvement for the lncRNAs, which includes the AML prognostic model, the TCGA-LAML data set was separated into high- and low-risk groups based on the model’s risk score. We used the “org.Hs.eg.db,” “clusterProfiler,” and “enrichplot” packages for enrichment analysis and selected the top five GO (Gene Ontology) and Kyoto Encyclopedia of Genes and Genomes (KEGG) terms and pathways to draw enrichment curves.

### 2.9 Statistical analyses

We performed statistical analyses in RStudio (R version 4.1.1). The lncRNA expression data set and OS were analyzed using univariate and multivariate Cox regression analysis methods, and prognosis was estimated by generating Kaplan–Meier survival curves. Correlations between the risk score of the prognostic model and tumor microenvironment scores were assessed using the Spearman’s correlation analysis. All statistical tests were two-sided, and *p* < 0.05 was considered significant.

## 3 Results

### 3.1 Obtaining immunity-related differentially expressed RNAs in AML

**FIGURE 1:**
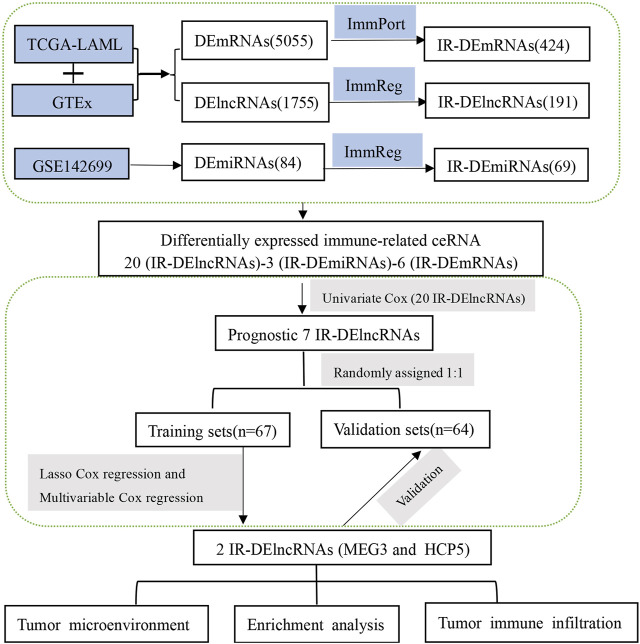
The workflow displays the process leading to identification of two IR-DElncRNAs with prognostic predictive value in AML.

By combining the TCGA-LAML and GTEx data sets, we identified differentially expressed (DE) transcripts between AML patients (*n* = 150) and healthy controls (*n* = 70). A total of 5,055 DEmRNAs (2,496 upregulated and 2,559 downregulated) and 1,755 DElncRNAs (966 upregulated and 789 downregulated) were detected. In turn, analysis of differential miRNA expression between 24 AML and 24 normal tissue samples was performed in the GSE142699 data set. In this analysis, 84 DEmiRNAs (65 upregulated and 19 downregulated) were found altogether. In addition, a total of 1,873 IR-lncRNAs and 650 IR-miRNAs were obtained from the ImmReg database, 1,793 IRmRNAs were obtained from the ImmPort database, and 424 IR-DEmRNAs, 191 IR-DElncRNAs, and 69 IR-DEmiRNAs were obtained from the corresponding intersection analysis. The study workflow is shown in Figure 1.

### 3.2 Construction of immunity-related ceRNA network

**FIGURE 2:**
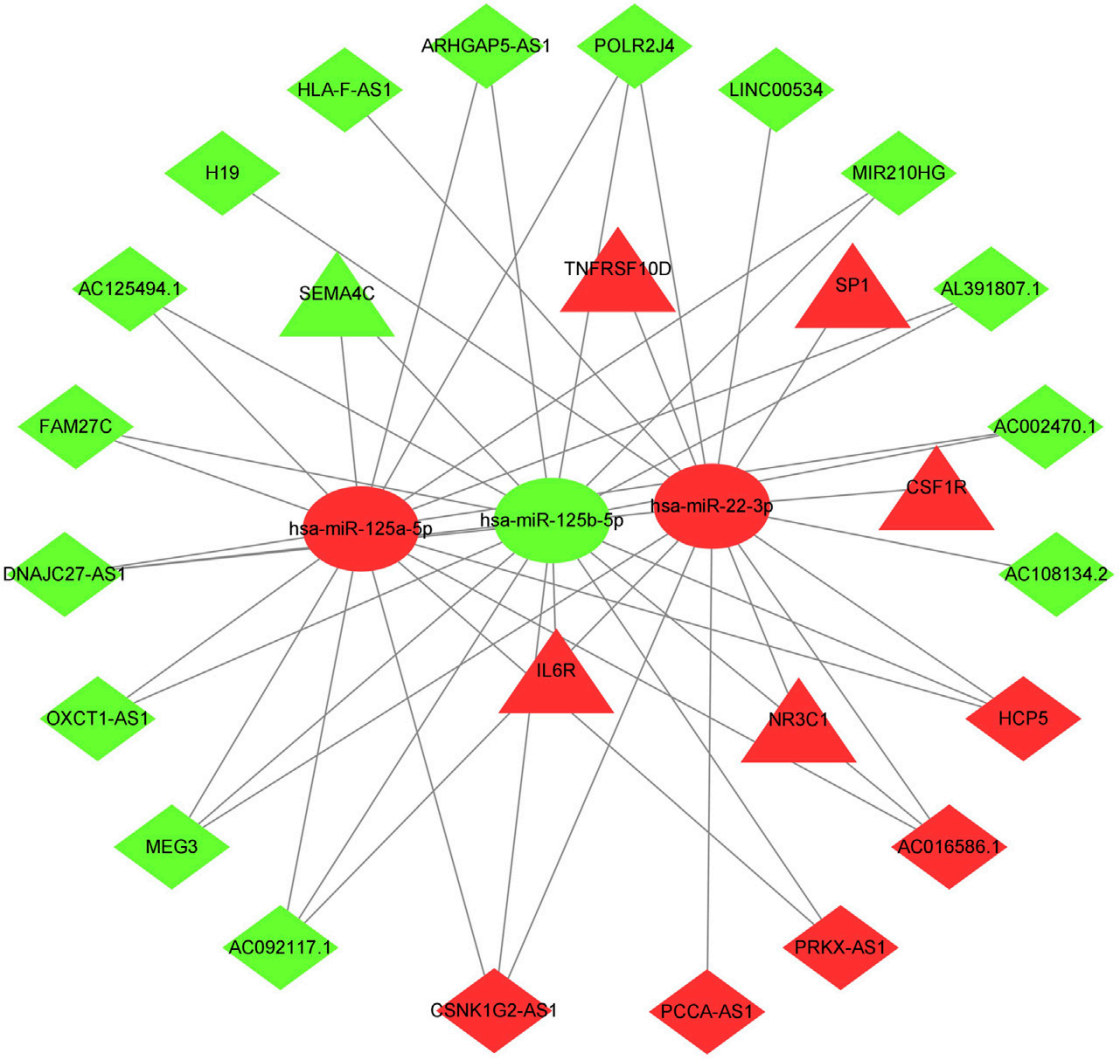
ceRNA network of immune-related lncRNA-miRNA-mRNA transcripts in AML. Triangles represent IR-DEmRNAs, ovals represent IR-DEmiRNAs, and rhombuses represent IR-DElncRNAs. Red represents upregulated expression, and green represents downregulated expression.

The previously extracted 191 IR-DElncRNAs were predicted by miRcode to interact with 85 miRNAs. Among these miRNAs, three transcripts, namely, hsa-mir-22-3p, hsa-mir-125b-5p, and hsa-mir-125a-5p had been categorized as IR-DEmiRNAs in our preceding analysis. Upon examining the three databases mentioned in Section 2.4, 103 target mRNAs were predicted to interact with the three aforementioned miRNAs. Among these mRNAs, six were identified as IR-DEmRNAs based on our previous analysis. Then, we obtained the immunity-related ceRNA networks, which included 20 IR-DElncRNAs (HCP5, PCCA-AS1, AC016586.1, PRKX-AS1, CSNK1G2-AS1, AC092117.1, ARHGAP5-AS1, AC125494.1 AC108134.2, DNAJC27-AS1, POLR2J4, MEG3, OXCT1-AS1, AC002470.1, HLA-F-AS1, AL391807.1, FAM27C, MIR210HG, H19, and LINC00534), 6 IR-DEmRNAs (SP1, TNFRSF10D, IL6R, CSF1R, SEMA4C, and NR3C1), and 3 IR-DEmiRNAs (hsa-mir-22-3p, hsa-mir-125a-5p, and hsa-mir-125b-5p) (Figure 2).

### 3.3 Construction of IR-DElncRNA–based prognostic model for AML

**FIGURE 3:**
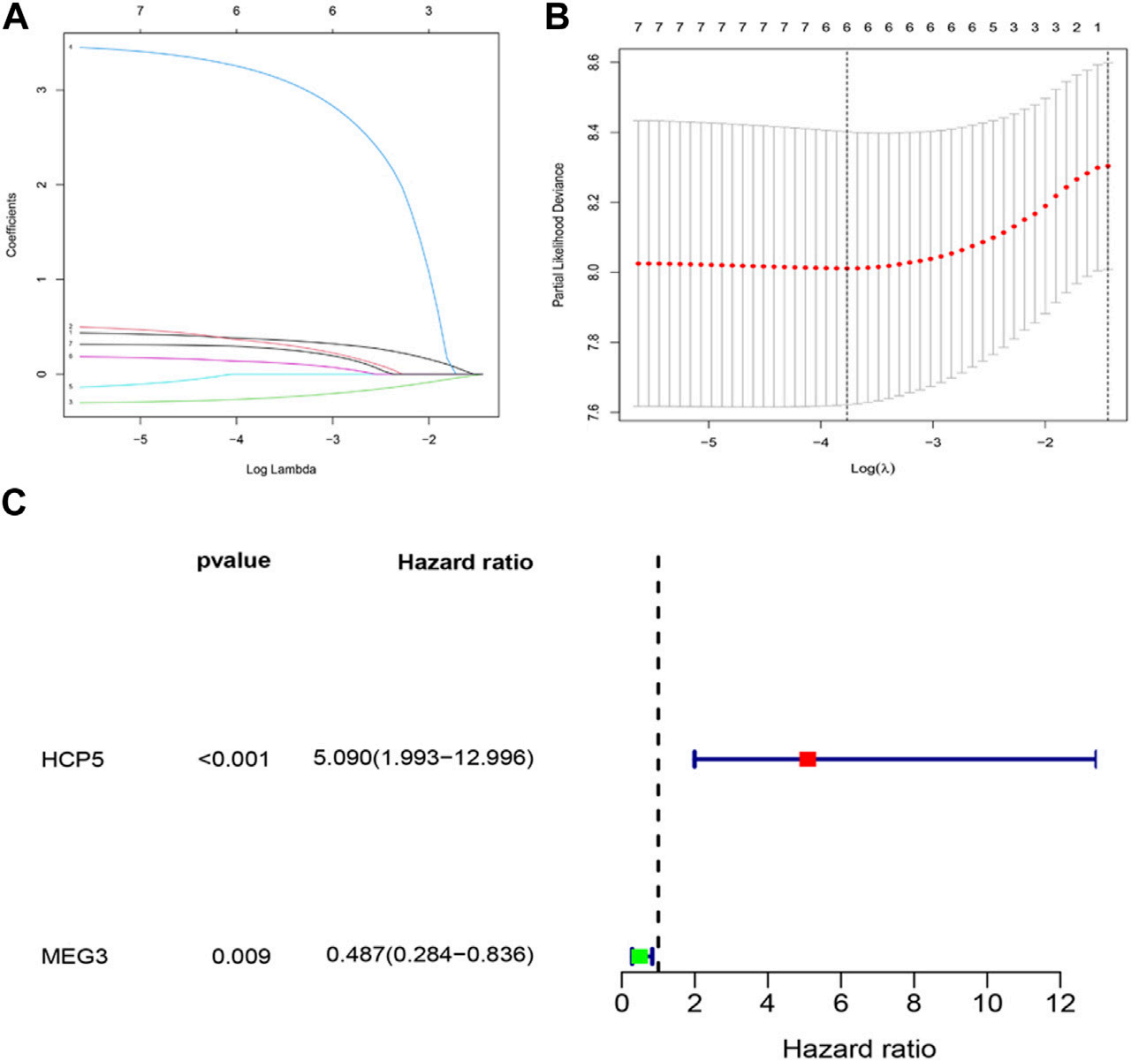
Construction of an IR-DElncRNA–based prognostic model for AML. **(A)** Validation of parameter selection was conducted by the LASSO regression model for OS. **(B)** LASSO coefficient profiles of prognostic IR-DElncRNAs. **(C)** Forest plot depicting HR with 95% CI of prognostic IR-DElncRNAs in AML, according to multivariate Cox regression. HR, hazard ratios; CI, confidence intervals.

The effect of IR-DElncRNAs, which includes those in the ceRNA network, on the prognosis of AML patients was assessed with univariate Cox regression analysis (Supplementary Table S1). Based on these results, seven IR-DElncRNAs were selected for further analyses (Supplementary Table S2). According to the clinical characteristics of the patients, the TCGA-LAML data set was divided randomly (1:1 ratio) into training sets (*n* = 67) and validation sets (*n* = 64), with detailed information included such as the TCGA ID, age, and gender of the patients of the training and validation sets that is shown in Supplementary Tables S3, S4. Subsequently, two IR-DElncRNAs were screened by LASSO (Figures 3A, B) and multivariate Cox regression analyses (Supplementary Table S5). According to the findings, the high expression of human histocompatibility leukocyte antigen (HLA) complex P5 (HCP5) lncRNA in AML patients is a risk factor of adverse prognosis [HR = 5.090, 95% CI (1.993, 12.996), and *p* < 0.001], whereas the low expression of maternally expressed gene 3 (MEG3) lncRNA is a protective prognostic factor [HR = 0.487, 95% CI (0.284 and 0.836), and *p =* 0.009] (Figure 3C).

**FIGURE 4:**
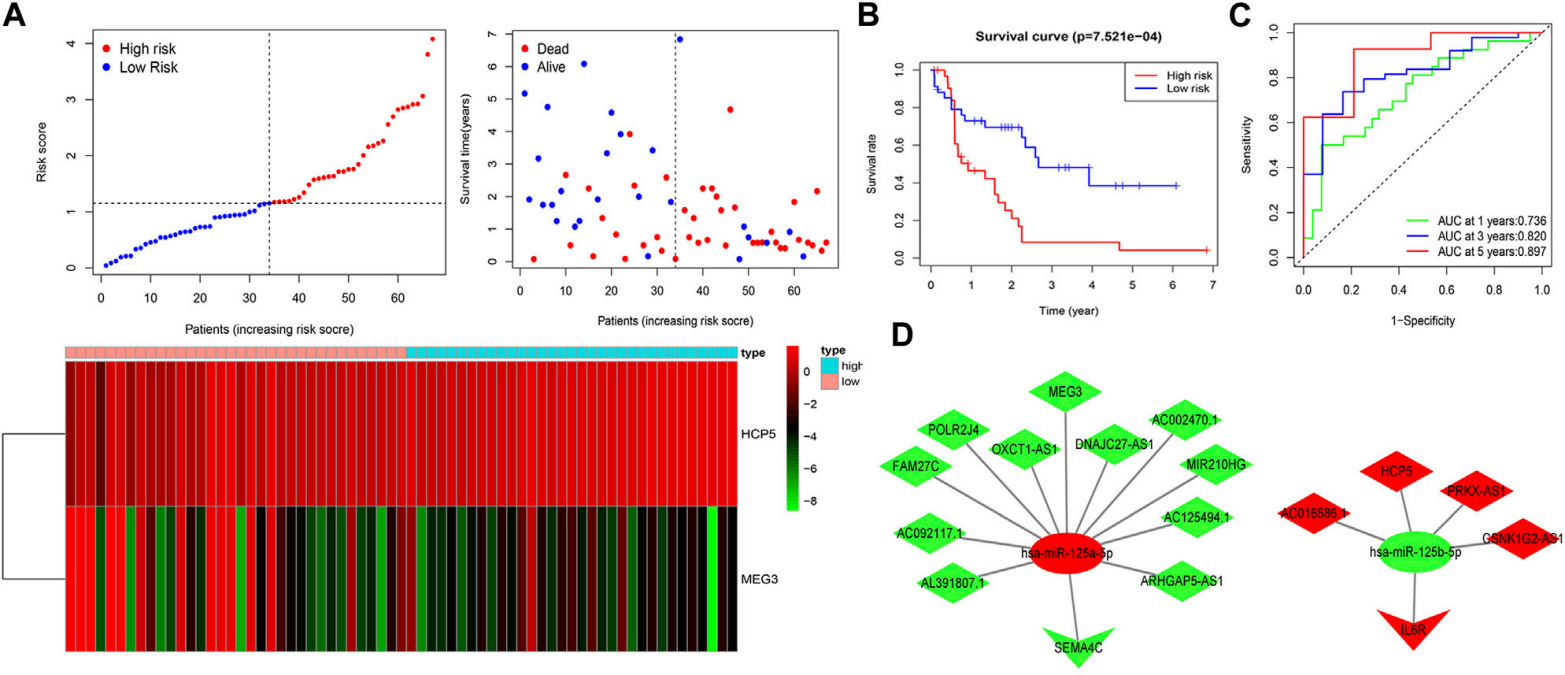
Construction of two IRlncRNAs–based prognostic models in AML. **(A)** AML patients were classified by the increasing risk score (left) and living status (right). The heatmap depicts the two IRlncRNAs–based expression profiles contained in the model for the low- and high-risk groups in the training set. **(B)** Kaplan–Meier analysis of OS for AML patients on the basis of risk stratification in the training set. **(C)** ROC for 1-, 3-, and 5-year OS prediction for AML patients in the training set. **(D)** ceRNA subnetworks based on each IRlncRNA included in the prognostic model. Triangles represent IR-DEmRNAs, ovals represent IR-DEmiRNAs; and rhombuses represent IR-DElncRNAs. Red represents upregulated expression, and green represents downregulated expression.

To estimate the ability of the lncRNA-based prognostic model to predict survival in patients, we assigned a risk score to each AML patient based on the formula risk score = (1.62 × HCP5 expression value) + (−0.72 × MEG3 expression value). Then, we found that when compared with the low-risk group, the mortality rate of the high-risk group was higher based on the median risk score in the training set. Accordingly, individual survival status curves indicated that the risk of death increased with increased risk score. Risk scores, survival time plots, and a heatmap depicting gene expression values for the prognostic model’s lncRNAs in the two patient cohorts are shown in Figure 4A. It can be seen that in the low-risk group, the lncRNA MEG3 is highly expressed, while the lncRNA HCP5 is highly expressed in the high-risk group. Supporting the model’s strength, consistent findings were obtained in the validation set (Supplementary Figure S1A). Survival analysis indicated that in the training set, the OS of patients in the high-risk group was lower than in the low-risk group (*p* < 0.001) (Figure 4B); meanwhile, similar results were obtained in the validation set (*p* < 0.05) (Supplementary Figure S1B). We then plotted the ROC curves and the area under the curve (AUC) of the prognostic model to predict 1-, 3-, and 5-year OS that was 0.736, 0.820, and 0.897 in the training set (Figure 4C) and 0.708, 0.710, and 0.750 in the validation set (Supplementary Figure S1C). The PCA was further used to verify the differences in the OS between the low- and high-risk groups in the training and validation sets (Supplementary Figures S1D, E). Based on the predictive model, two ceRNA regulatory subnetworks, namely, MEG3/miR-125a-5p/SEMA4C and HCP5/miR-125b-5p/IL6R, involved in the immune regulation of AML prognosis were identified (Figure 4D).

### 3.4 Correlations between AML prognostic model and tumor microenvironment components

**FIGURE 5:**
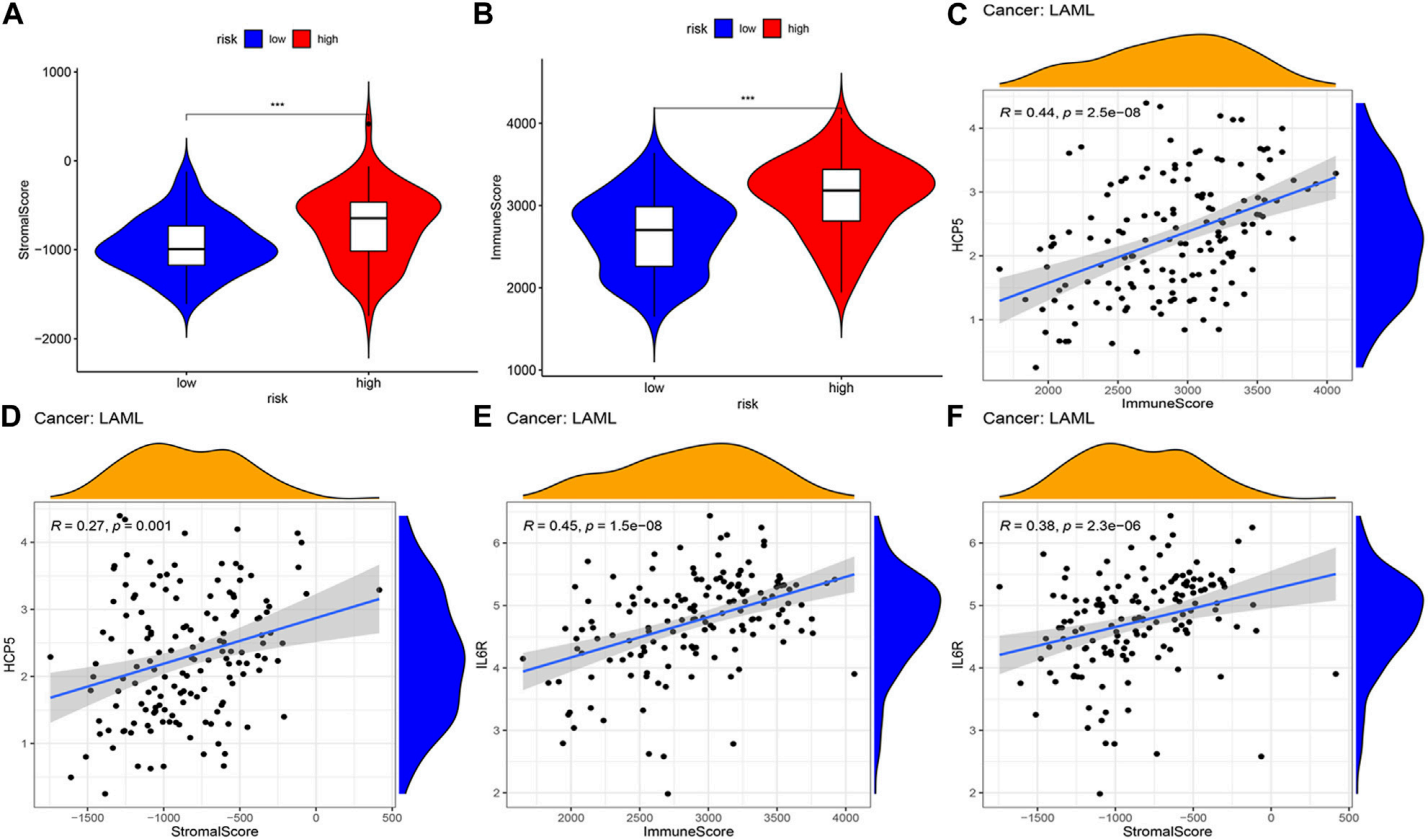
Correlation analysis of the two IRlncRNAs–based prognostic model and tumor microenvironment scores in AML. **(A)** Distribution of stromal scores in different risk groups. **(B)** Distribution of immune scores between the two risk groups. **(C, D)** Regression plots depicting the relationship between expression levels of HCP5 and immune scores **(C)**, and the stromal scores **(D)**. **(E, F)** Regression plots depicting the relationship between expression levels of IL6R and immune **(E)** and stromal scores **(F)**.

The correlations between the AML prognostic model and immunity-related cells and pathways were explored by assessing the differences in immune cell infiltration between the two AML patient groups. Using ESTIMATE, the stromal and immune scores of each AML sample were first calculated. Patients having higher stromal scores (−724.9 vs. −952.6, respectively, and *p* < 0.001) and higher immune scores (2,663.7 vs. 3,103.6, respectively, and *p* < 0.001) in the high-risk group were compared to that of the low-risk group (Figures 5A, B), showing more extensive immune cell infiltrations in the high-risk group. The analysis of the correlation between the expression level (log2 transformation) of members of the two ceRNA subnetworks and the immune and stromal scores revealed a positive correlation between the lncRNA HCP5 expression level and the immune score (R = 0.44 and *p =* 2.5e−08) and the stromal score (R = 0.27 and *p =* 0.001) (Figures 5C, D). Similarly, the mRNA IL-6 receptor (IL6R) expression level, the immune score (R = 0.45 and *p =* 1.5e−08), and the stromal score (R = 0.38, *p =* 2.3e−06) presented a positive correlation (Figures 5E, F). By contrast, no significant correlations with the immune and stromal scores were detected for SEMA4C mRNA and MEG3 lncRNA (Supplementary Figures S2A–D). These results suggest that in AML, the HCP5/miR-125b-5p/IL6R axis influences the composition of stromal and immune cells.

### 3.5 Correlation between AML prognostic model and tumor immune infiltration

**FIGURE 6:**
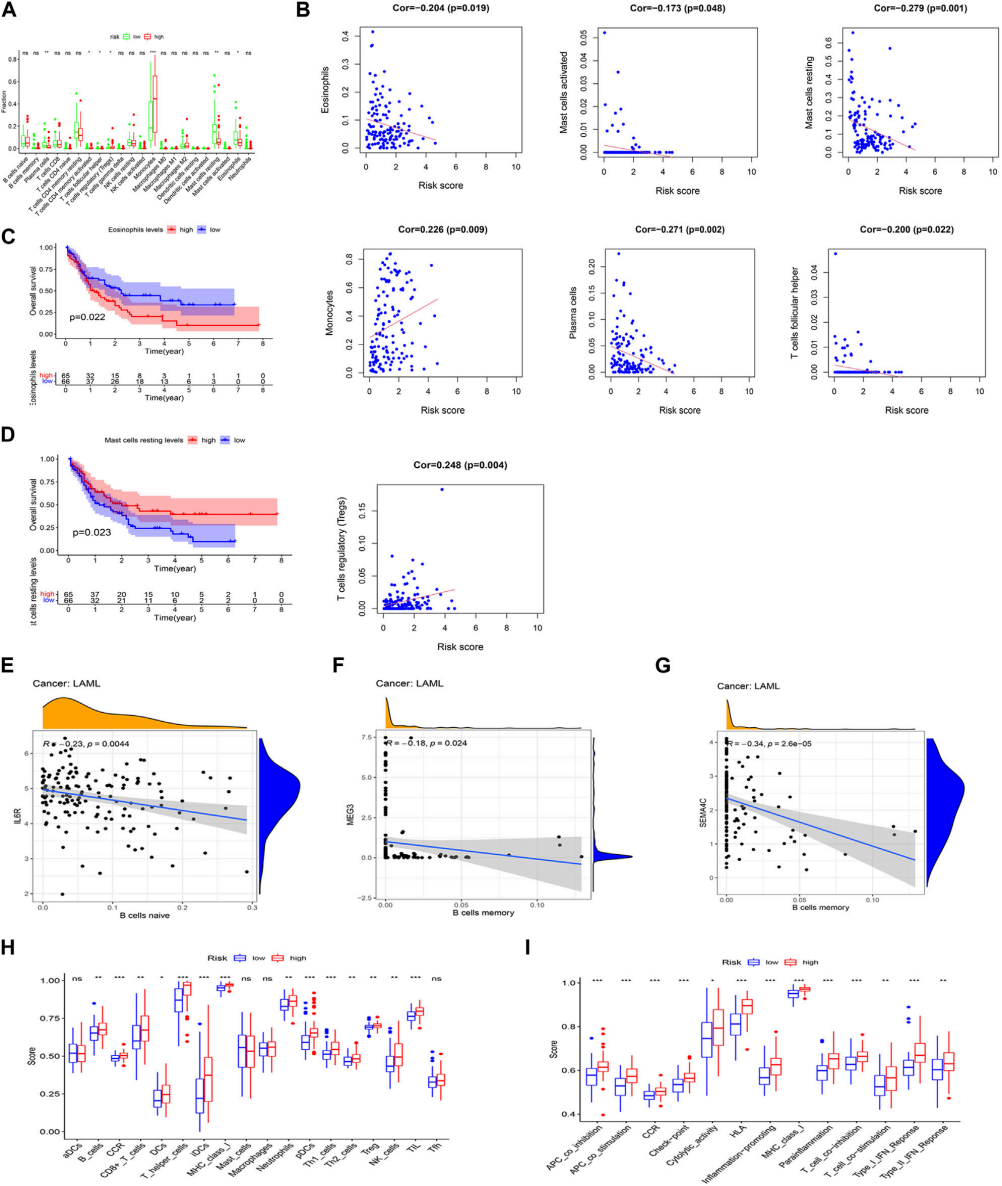
Association between the two IRlncRNAs–based prognostic model and immune signatures. **(A)** Immune score enrichment analysis for 22 tumor-infiltrating immune cell types for different risk groups. **(B)** Relationships between the model’s risk score and infiltrating immune cell levels (*p* < 0.05). **(C,D)** Survival analysis based on eosinophil levels **(C)** and resting mast cell levels **(D)**. **(E)** Correlation between IL-6R expression and infiltration levels of naive B cells. **(F)** Relationship between the expression level of SEMA4C and memory B cell infiltration. **(G)** Relationship between the expression level of lncRNA MEG3 and abundance of memory B cells. **(H)** Score distributions for 18 tumor-infiltrating immune cell types for different risk groups. **(I)** Score distributions for 13 immune functions between the two risk groups.

The CIBERSORT was applied to analyze the relationship between the prognostic model and 22 tumor-infiltrating immune cell types. The finding showed that the plasma cell, follicular helper T cell, resting mast cell, and eosinophil levels were significantly decreased, whereas the CD4 memory-activated T cell, regulatory T cell (Treg), and monocyte levels were significantly increased, in the high-risk group (Figure 6A). In turn, the finding showed a positive correlation between monocytes and Treg levels and risk scores, whereas a negative correlation was found among the eosinophil, activated mast cell, resting mast cell, plasma cell, and follicular helper T cell levels and risk scores (*p* < 0.05) (Figure 6B). Interestingly, significant correlations with lower OS were detected for high eosinophil infiltration levels (*p =* 0.022) and low resting mast cell infiltration levels (*p =* 0.023) (Figures 6C, D). Meanwhile, we found a negative correlation between the expression level of IL-6R and naive B cell infiltrations (*p* < 0.05), but a negative correlation between the expression levels of SEMA4C and MEG3 and memory B cell infiltrations (*p* < 0.05) (Figures 6E–G) analyzed by using the Spearman’s test.

The correlations between the risk score of the prognostic model and infiltrating immune cells (Figure 6H) and immune functions (Figure 6I) for the two risk groups in the TCGA-LAML data set were compared using the ssGSEA. When compared with the low-risk group, the high-risk group had higher representations of B cells, CD8^+^ T cells, DCs, helper T cells, iDCs, neutrophils, pDCs, Th1 cells, Th2 cells, Tregs, NK cells, and TILs. In turn, the high-risk group had higher scores for several immune functions, such as APC co-inhibition, APC co-stimulation, CCR, check-point, cytological activity, HLA, inflammation-promoting activity, MHC class I, para-inflammation, T-cell co-inhibition, T-cell co-stimulation, type I IFN response, and type II IFN response when compared with the low-risk group. Further analysis of the identified ceRNA subnetworks indicated that the SEMA4C expression was positively correlated with aDCs, and IL6R and lncRNA HCP5 were both positively correlated with APC co-inhibition, whereas SEMA4C and lncRNA MEG3 were both negatively correlated with APC co-inhibition. These findings, therefore, suggest that our prognostic model will be useful to infer immune cell distribution and relevant immune pathways influencing the prognostic of AML patients.

### 3.6 Enrichment analysis based on prognostic model

**FIGURE 7:**
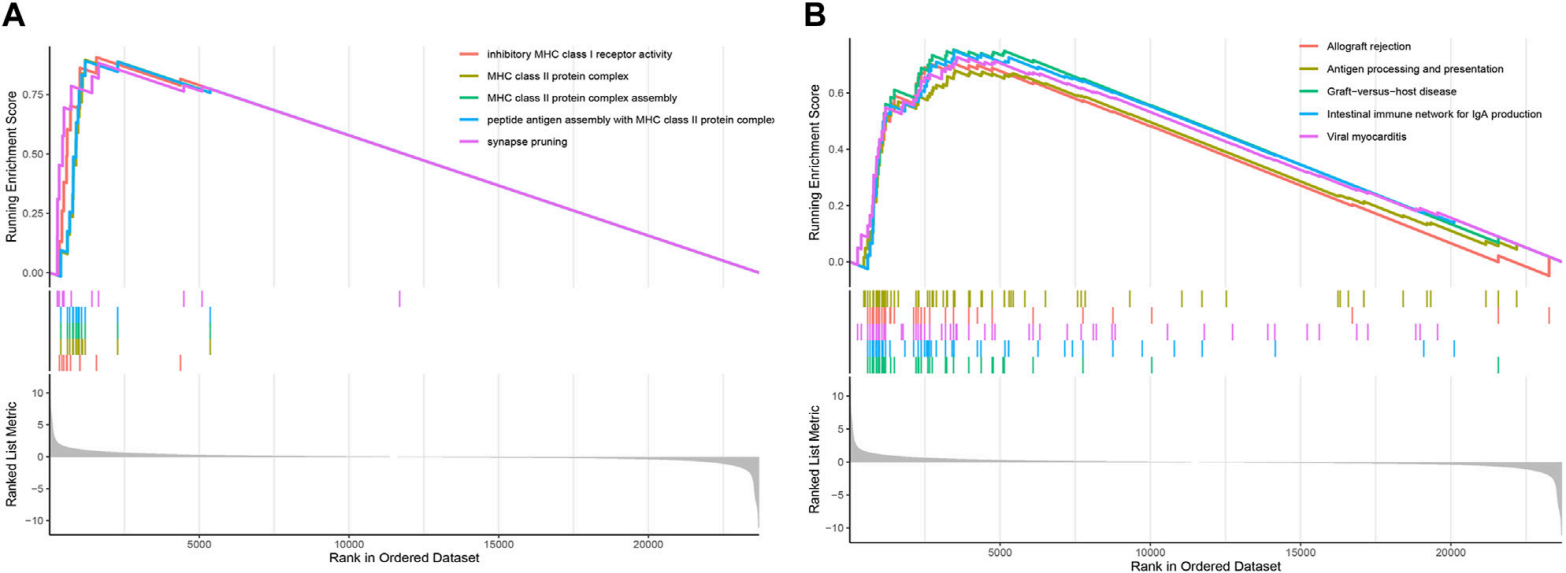
GSEA based on the AML prognostic model. **(A)** GSEA enrichment plot displaying the top five GO terms. **(B)** GSEA enrichment plot showing the top five KEGG pathways.

To determine the relevant signaling pathways associated with our two lncRNAs signature model, the gene set enrichment analysis (GSEA) was carried out on the transcriptional profiles of the low- and high-risk groups identified in the TCGA-LAML cohort. The GO analysis showed that the high-risk group was mainly enriched in inhibitory MHC class I receptor activity, MHC class II protein complex, MHC class II protein complex assembly, peptide antigen assembly with MHC class II protein complex, and synapse pruning (Figure 7A). The KEGG enrichment analysis showed in turn that the high-risk group was predominantly enriched in pathways related to graft-versus-host disease, internal immune network for IgA production, viral myocarditis, allograft rejection, and antigen processing and presentation (Figure 7B).

## 4 Discussion

AML is an aggressive hematological malignant tumor with high incidence and mortality rates that is featured by myeloid progenitor cells’ abnormal proliferation and differentiation (Shallis et al., 2019). The accurate prediction of AML patients’ OS is of great significance for selecting treatment and improving prognosis. There are so far no reliable and effective biomarkers and prognostic models to accurately predict the survival rate of AML patients. All kinds of non-coding RNAs that impact the pathogenesis in AML has previously been reported (Estey, 2020). Salmena et al. (2011) had put forward the ceRNA hypothesis in 2011, which hypothesized that different RNA species (lncRNAs, mRNAs, circRNAs, and transcribed pseudogenes) can competitively combine with miRNAs to induce miRNA-mediated gene expression silencing. Subsequently, several studies have addressed the potential influence of ceRNA networks in the development and treatment of AML (Sanchez-Mejias and Tay, 2015; Thomson and Dinger, 2016). For example, lncRNAs CCAT1, SBF2-AS1, and UCA1 were shown to upregulate the expressions of c-Myc, ZFP91, and HK2 by binding to miR-155, miR-188-5p, and miR-125a, respectively, and thus promoting AML cell proliferation (Chen et al., 2016; Zhang et al., 2018; Tian et al., 2019). However, studies on the regulatory effects of lncRNA-miRNA-mRNA interactions in AML, especially those involving immunity-related ceRNA networks, are still limited. Recent research has focused on building prediction models according to non-coding RNA expression data to evaluate the prognosis of AML patients. These include miRNA-based models (Tian et al., 2018; Zhu et al., 2019), clinical feature–based models (Wang et al., 2020), lncRNA-based prognostic models (Garzon et al., 2014; Li and Sun, 2018), and ceRNA network–based prognostic models (Wang et al., 2019). However, the potential of immunity-related lncRNAs and ceRNA networks in predicting AML prognosis remains uncertain.

This study examined the TCGA, GEO, ImmReg databases to collect, respectively, AML-related gene expression data, AML-related miRNA expression data, and gene sets associated with immunity-related pathways. As a result, 424 IR-DEmRNAs, 191 IR-DElncRNAs, and 69 IR-DEmiRNAs were screened, and a ceRNA network which included 20 IR-DElncRNAs, 6 IR-DEmRNAs, and 3 IR-DEmiRNAs was finally established. After evaluating the impact of each of these 20 IR-DElncRNAs on AML survival, a prognostic model containing two IR-DElncRNAs (MEG3 and HCP5) was performed according to LASSO and multivariate Cox regression analyses. Interaction analyses on lncRNAs included in the prognostic model revealed two regulatory ceRNA axes potentially involved in the immune regulation of AML prognosis, namely, lncRNA MEG3/miR-125a-5p/SEMA4C and lncRNA HCP5/miR-125b-5p/IL6R.

The study of tumor-regulating lncRNAs has aroused wide concern and increased the use of high-throughput sequencing techniques. Numerous non-coding RNAs, such as the three lncRNA transcripts, TUG1 (Wang X. et al., 2018a; Luo et al., 2018), LINC00899 (Wang Y. et al., 2018b), and PANDAR (Yang et al., 2018), have been found to be closely related to cell cycle dynamics and apoptosis in AML. These studies, along with the present one, suggest that lncRNAs may be useful biomarkers for diagnosis, prognosis, and therapy of AML. lncRNA HCP5, included in our AML prognostic model, is mainly expressed in immune cells and participates in innate and adaptive immune reactions. By acting as a ceRNA, its potential contribution to the onset, development, and/or drug resistance of thyroid carcinoma, colorectal cancer, and pancreatic cancer has been reported (Liu et al., 2019). In addition, several research studies have revealed abnormal HCP5 expression that correlates with the prognosis of many cancers, making it a potential prognostic biomarker (Liu et al., 2019; Yang et al., 2019; Gao et al., 2021). Hu et al. (2021) used GEPIA2 to evaluate HCP5 expression levels and survival associations in different cancers, and the findings showed that HCP5 was upregulated in cholangiocarcinoma, esophageal carcinoma, AML, and pancreatic adenocarcinoma, and both OS and disease-free survival were lower in patients with high HCP5 expression. In line with the present findings, this evidence supports a deleterious influence of HCP5 overexpression on OS in various types of cancer.

The second biomarker included in our AML prognostic model, namely, MEG3, is a recently found lncRNA with tumor-suppressive function that is very critical at the onset and development of several cancers. MEG3 is significantly downregulated in several human tumors and tumor cells. In different solid tumors and in diverse tumor cell lines, MEG3 overexpression inhibits proliferation, hence it is used as a tumor suppressor gene (Zhou et al., 2007; Zhang et al., 2010; Balik et al., 2013). Several mechanisms, such as P53-mediated transcriptional regulation (Benetatos et al., 2011) and promoter region CpG island hypermethylation (Braconi et al., 2011; Lu et al., 2013), have been reported to repress MEG3 expression in different tumor types and tumor cell lines. In a series of 42 AML cases, a MEG3 promoter hypermethylation rate of 47.6% was reported in association with significantly reduced OS (Benetatos et al., 2010). Although the aforementioned studies have suggested promoter hypermethylation as the main cause of MEG3 downregulation in tumor cells, it remains unclear whether MEG3 suppression affects the growth of AML cells.

Abnormal miRNA expression was shown to disrupt important cellular processes, contributing to the initiation and progression of various diseases (Mehta and Baltimore, 2016). Studies have proposed distinct roles of miR-125 family members (miR-125a and miR-125b) in the inhibition and promotion of AML (Emamdoost et al., 2017; Hu et al., 2017; Liu et al., 2017). Our present study thus expands our understanding of the contribution of miR-125a and miR-125b to AML by demonstrating their prognostic significance as part of immunity-related ceRNA networks that include HCP5 and MEG3 as direct upstream regulators and IL6R and SEMA4C as target genes.

We also evaluated the association between our lncRNA-based prognostic model and the tumor microenvironment, which is very important in AML development and treatment (van Galen et al., 2019). The analysis of tumor-infiltrating immune cells in the TCGA-LAML patient cohort showed higher tumor infiltration levels of memory-activated CD4 T cells, Tregs, and monocytes in the high-risk group. However, monocyte and Treg levels were positively correlated with the risk score of the prognostic model. Our study further showed that the degree of infiltration of several immune cell types and the activity of various immune pathways were different among the high- and low-risk AML groups and that HCP5 expression level, but not that of MEG3, had a positive correlation between both immune and stromal scores. These data indicate that our two lncRNAs signature may be used as a predictor of differential immune cell infiltration in AML and highlight a probable role of lncRNAs HCP5 as an important determinant of the immune status in AML. Nevertheless, the molecular mechanisms linking dysregulated lncRNA expression and immune status, as well as the therapeutic impact of immunotherapies targeting the ceRNA networks identified herein, have to be explored.

## 5 Conclusion

Through the analysis of the gene expression data from AML patients, this study identified two OS-related IRlncRNAs and constructed a prognostic model on this basis that efficiently discriminates high-risk from low-risk AML cases. Based on this two IRlncRNAs prognostic signature, we also defined two corresponding immunity-related ceRNA networks with the potential impact on the stromal and immune composition of the AML microenvironment. Specifically, our analyses suggest that the lncRNAs HCP5 and MEG3 may act as key ceRNAs to modulate immune responses by regulating miR-125 species and their target genes. Our findings thus help to address the research gap on the role of IRlncRNAs in AML and provide a novel tool to predict prognosis and plan immunotherapy interventions. There are some limitations in this study. First, our analyses were based on a limited number of AML cases recorded on a public database. Hence, the current results have to be subjected to large-scale, multi-center external verification. Second, the biological functions of the two prognostic IRlncRNAs, i.e., their interactions with specific miRNAs and their post-transcriptional regulatory functions, have to be validated through molecular assays.

## Data Availability

The original contributions presented in the study are included in the article/Supplementary Material; further inquiries can be directed to the corresponding authors.
