# Supplementary material for: Identification of immunity-related lncRNAs and construction of a ceRNA network of potential prognostic biomarkers in acute myeloid leukemia
Source: Front Genet. 2023 Jun 14;14:1203345. doi: 10.3389/fgene.2023.1203345 (PMC10301753; doi:10.3389/fgene.2023.1203345)
Supplement: Supplementary file 1 [file DataSheet1.zip › Supplementary Material/Raw data.docx]

1.Raw data01

<https://www.jianguoyun.com/p/DVz-9RgQlqLHCxiP0oAFIAA>

2.Raw data02-08

<https://www.jianguoyun.com/p/Df4SRsAQk6fHCxjL0oAFIAA>
