# Supplementary material for: Identification of immunity-related lncRNAs and construction of a ceRNA network of potential prognostic biomarkers in acute myeloid leukemia
Source: Front Genet. 2023 Jun 14;14:1203345. doi: 10.3389/fgene.2023.1203345 (PMC10301753; doi:10.3389/fgene.2023.1203345)
Supplement: Supplementary file 1 [file DataSheet1.zip › Supplementary Material/Supplementary Figure Material.docx]

***Supplementary Material***

**Identification of immunity-related lncRNAs and construction of a ceRNA network of potential prognostic biomarkers in acute myeloid leukemia**

**Jia Xue, Haoran Chen, Jinqi Lu, Haojun Zhang, Peifeng He*, Xuechun Lu***

*** Correspondence:**

Xuechun Lu*, luxuechun@126.com

Peifeng He*, hepeifeng2006@126.com

**1. Supplementary Figures**


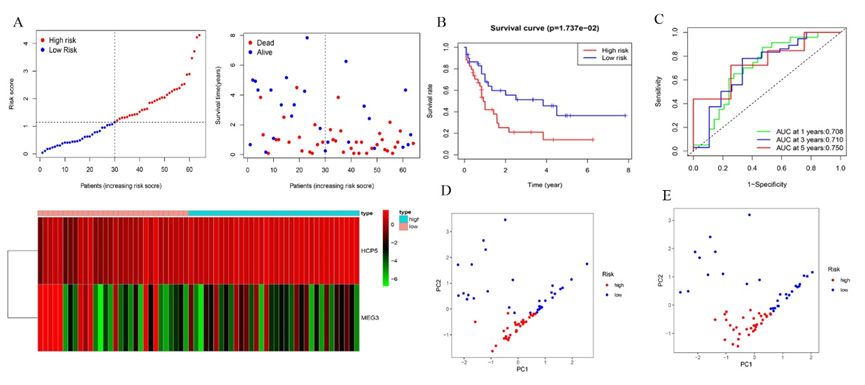


**Supplementary Figure 1** Construction of a 2-IRlncRNA-based prognostic model in AML. (A) AML patients were classified by increasing risk score (left) and living status (right). The heatmap depicts the expression profiles of the 2 IRlncRNAs included in the model for the different risk groups in the validation set. **(B)** Kaplan-Meier analysis of OS for AML patients based on risk stratification in the validation set. **(C)** ROC analysis for 1-, 3-, and 5-year OS prediction for AML patients in the validation set. **(D, E)** PCA analysis demonstrating the ability of the prognostic model to distinguish low-risk and high-risk AML patients in the training set (D) and in the validation set (E).


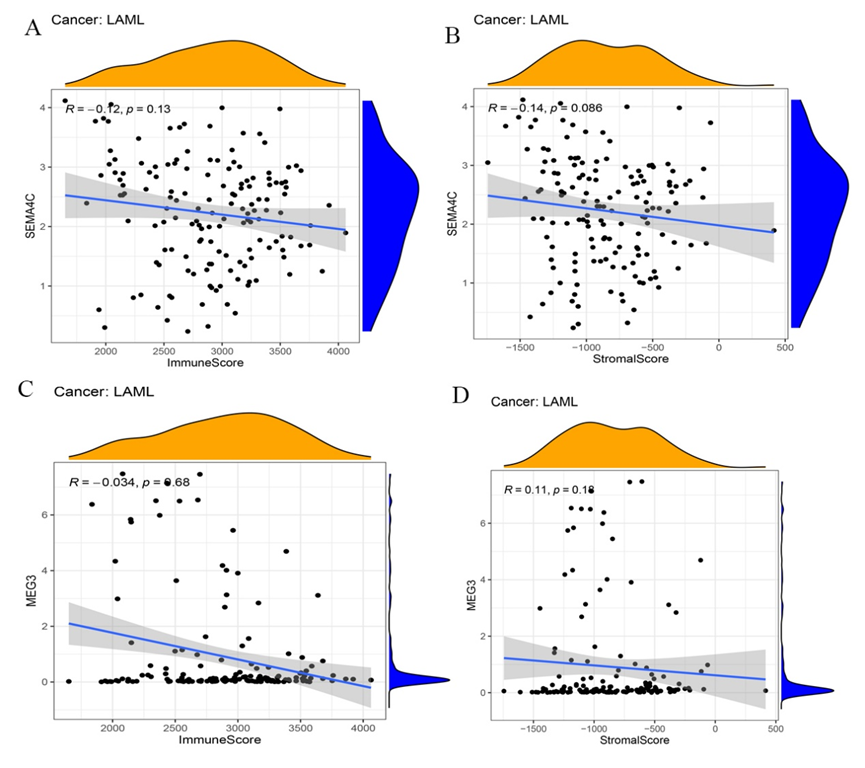


**Supplementary Figure 2** Correlations of the 2-IRlncRNA signature with the tumor microenvironment in AML. **(A, B)** Correlation between SEMA4C expression and immune and stromal scores. **(C, D)** Correlation between lncRNA MEG3 expression and immune and stromal scores.
