# Supplementary figures and images for: Identification of immunity-related lncRNAs and construction of a ceRNA network of potential prognostic biomarkers in acute myeloid leukemia
Source: Front Genet. 2023 Jun 14;14:1203345. doi: 10.3389/fgene.2023.1203345 (PMC10301753; doi:10.3389/fgene.2023.1203345)

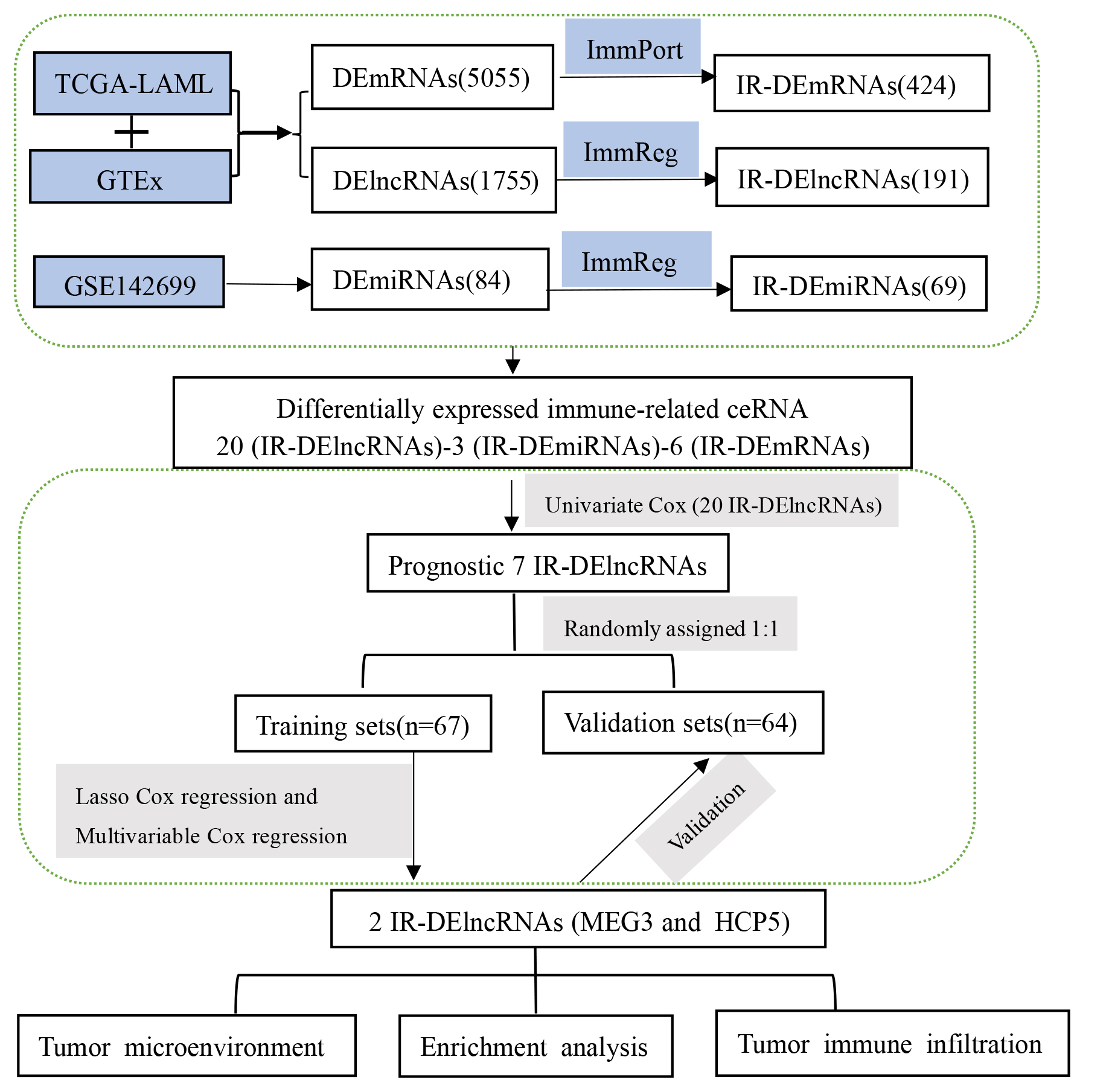

Supplement: Supplementary file 1 [file DataSheet1.zip › Supplementary Material/FIGURE/FIGURE 1.jpg]

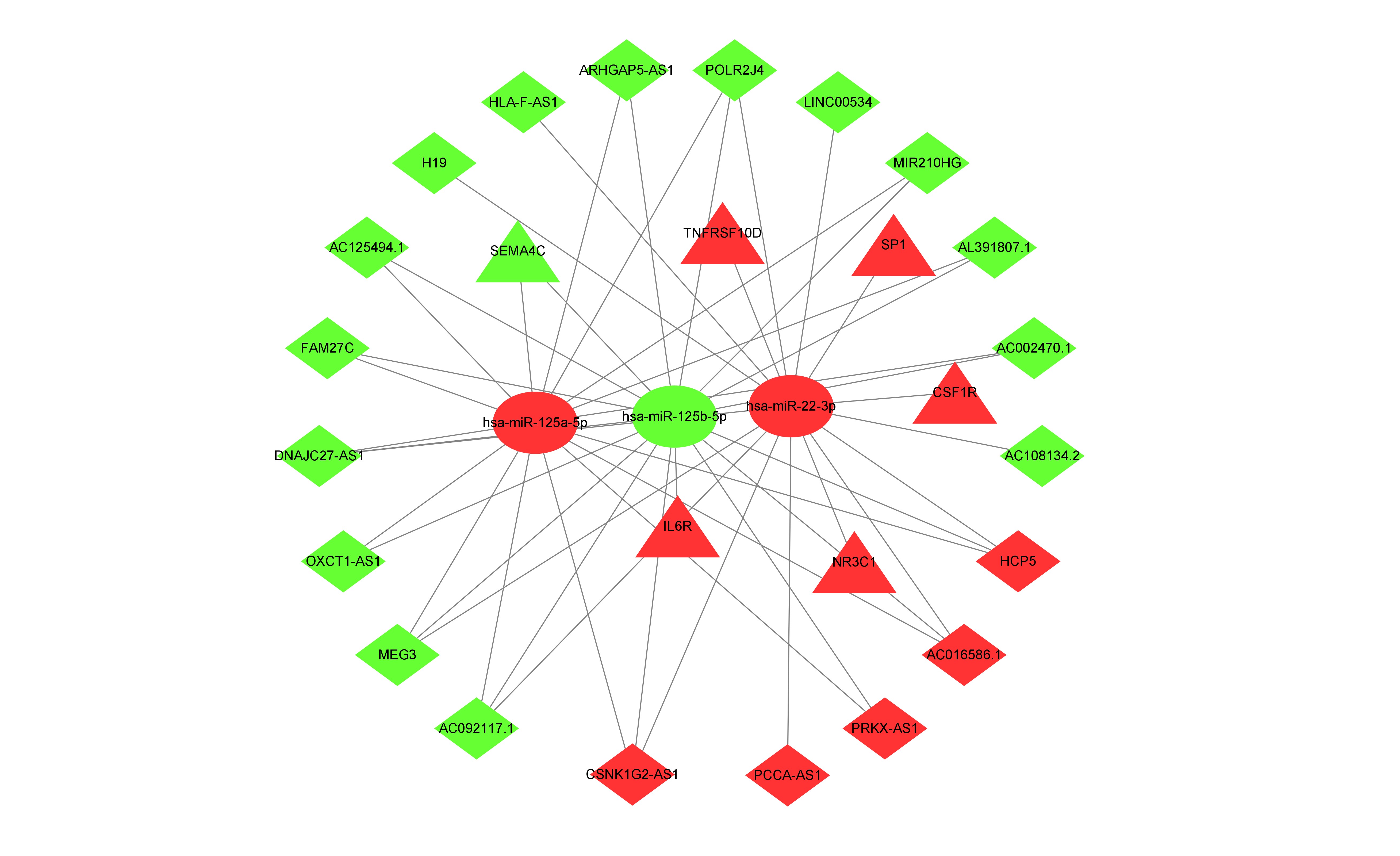

Supplement: Supplementary file 1 [file DataSheet1.zip › Supplementary Material/FIGURE/FIGURE 2.jpg]

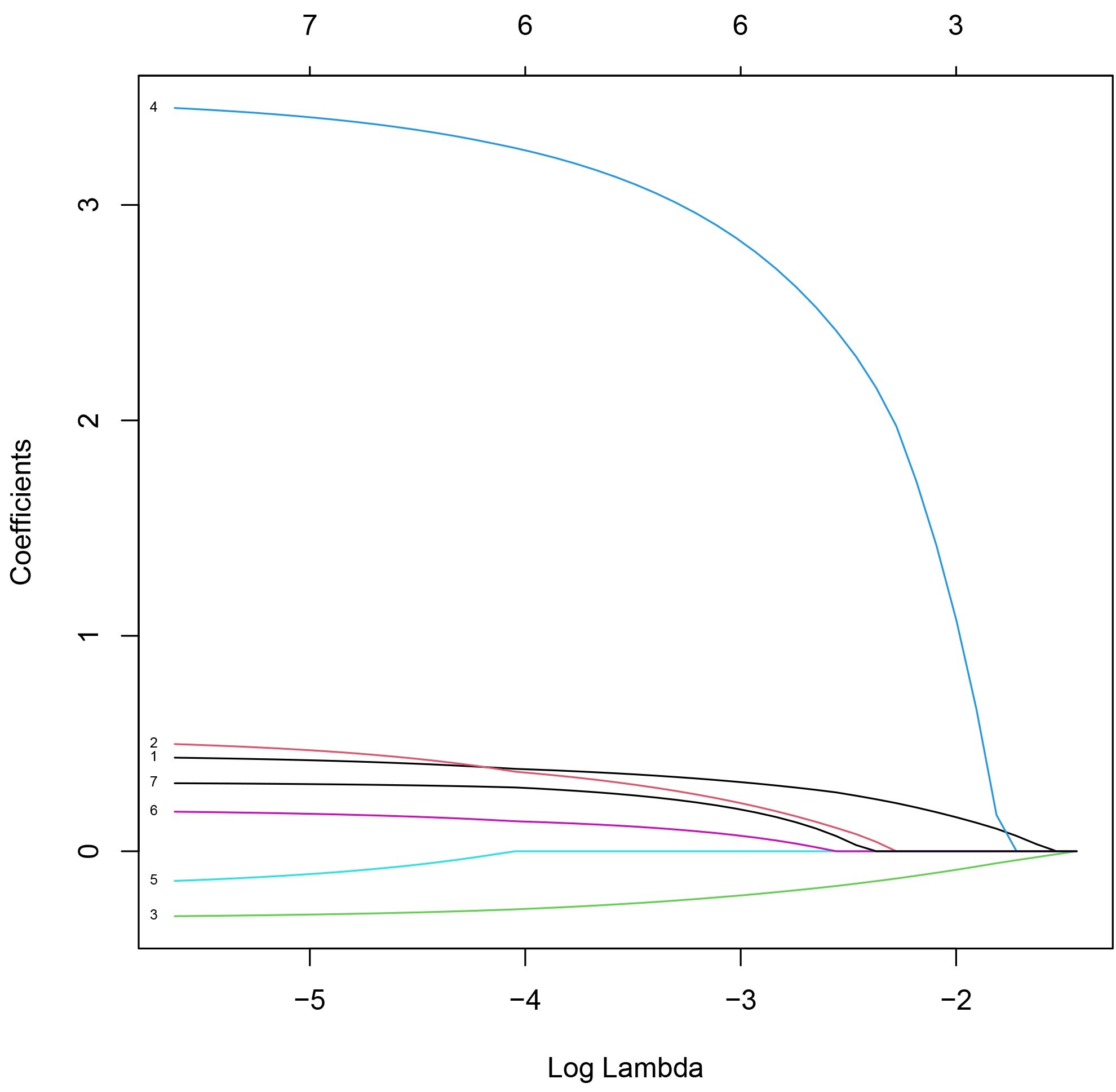

Supplement: Supplementary file 1 [file DataSheet1.zip › Supplementary Material/FIGURE/FIGURE 3/FIGURE 3A_lambda.jpg]

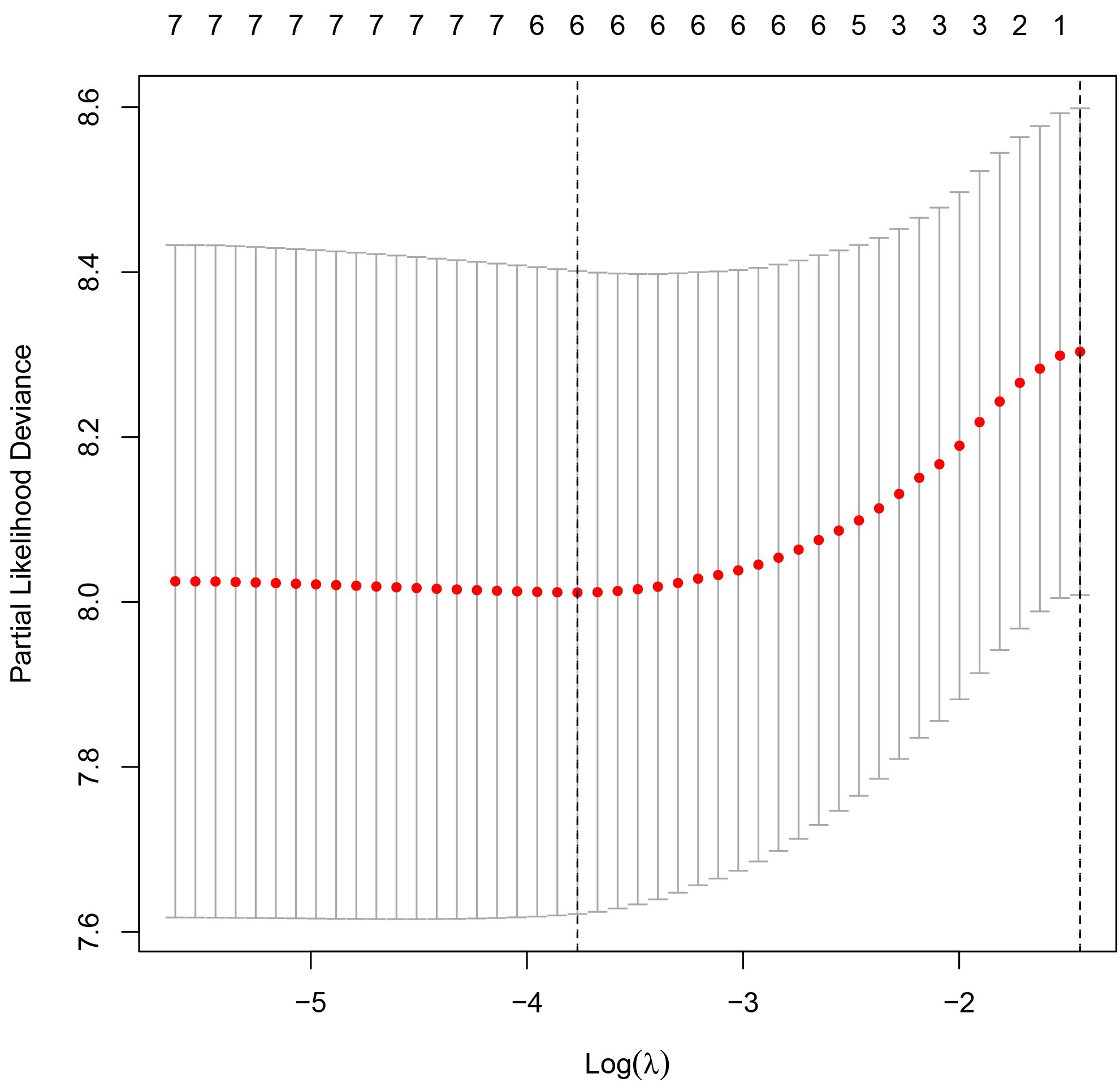

Supplement: Supplementary file 1 [file DataSheet1.zip › Supplementary Material/FIGURE/FIGURE 3/FIGURE 3B_cvfit.jpg]

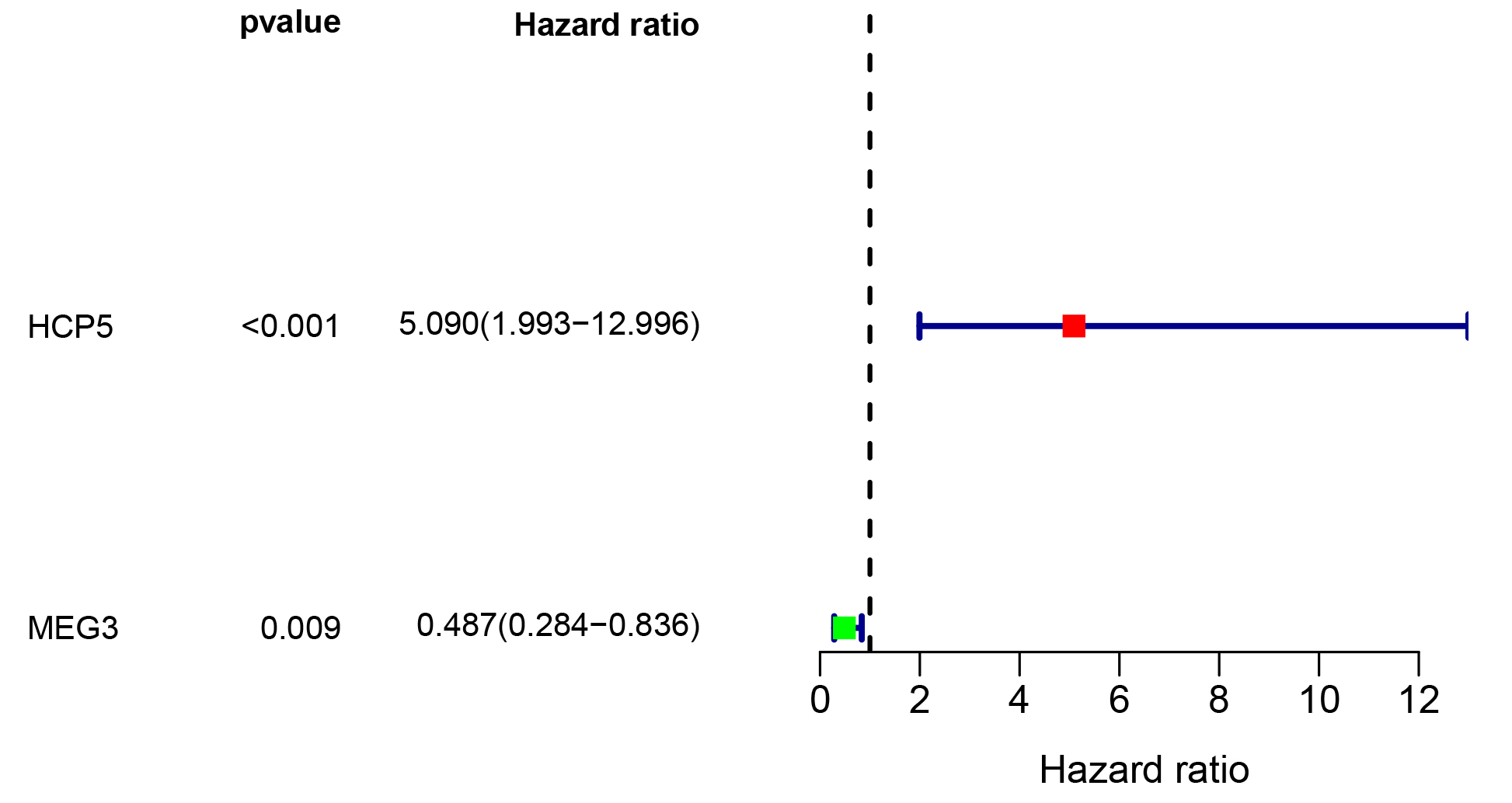

Supplement: Supplementary file 1 [file DataSheet1.zip › Supplementary Material/FIGURE/FIGURE 3/FIGURE 3C_forest.jpg]

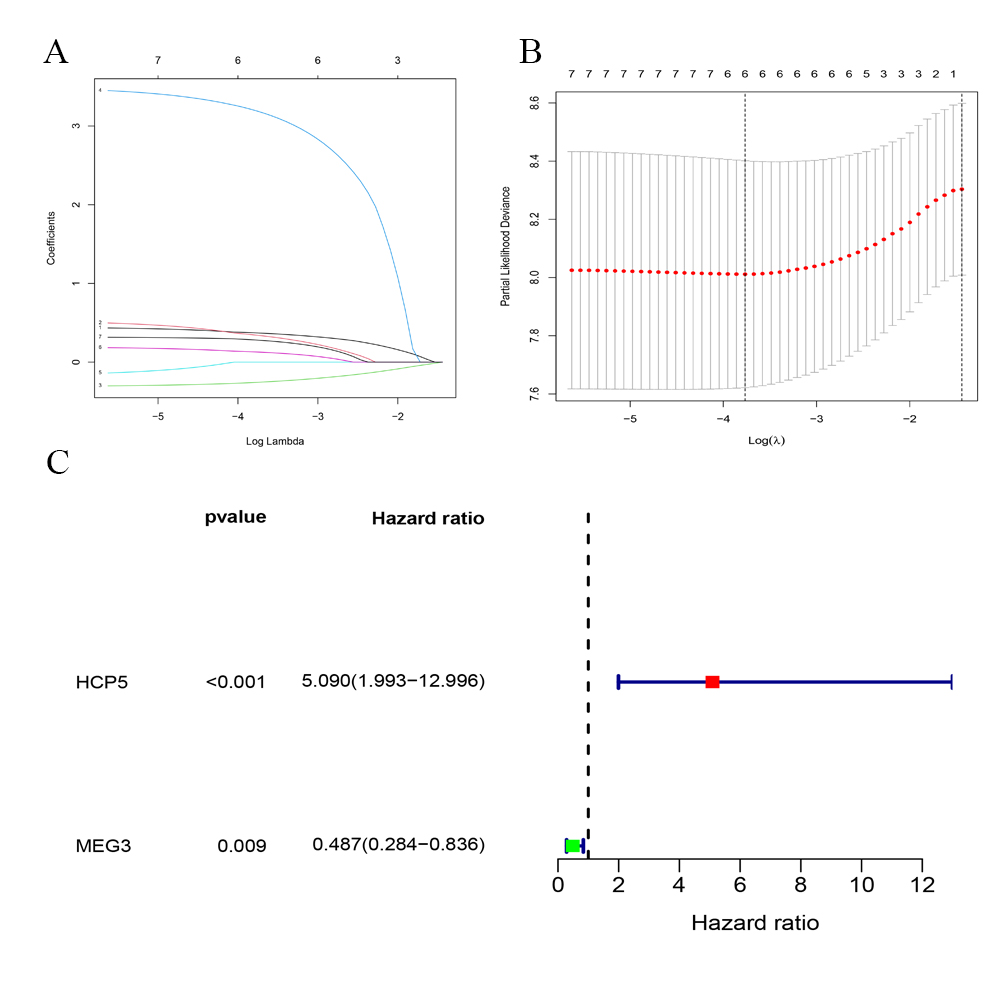

Supplement: Supplementary file 1 [file DataSheet1.zip › Supplementary Material/FIGURE/FIGURE 3.jpg]

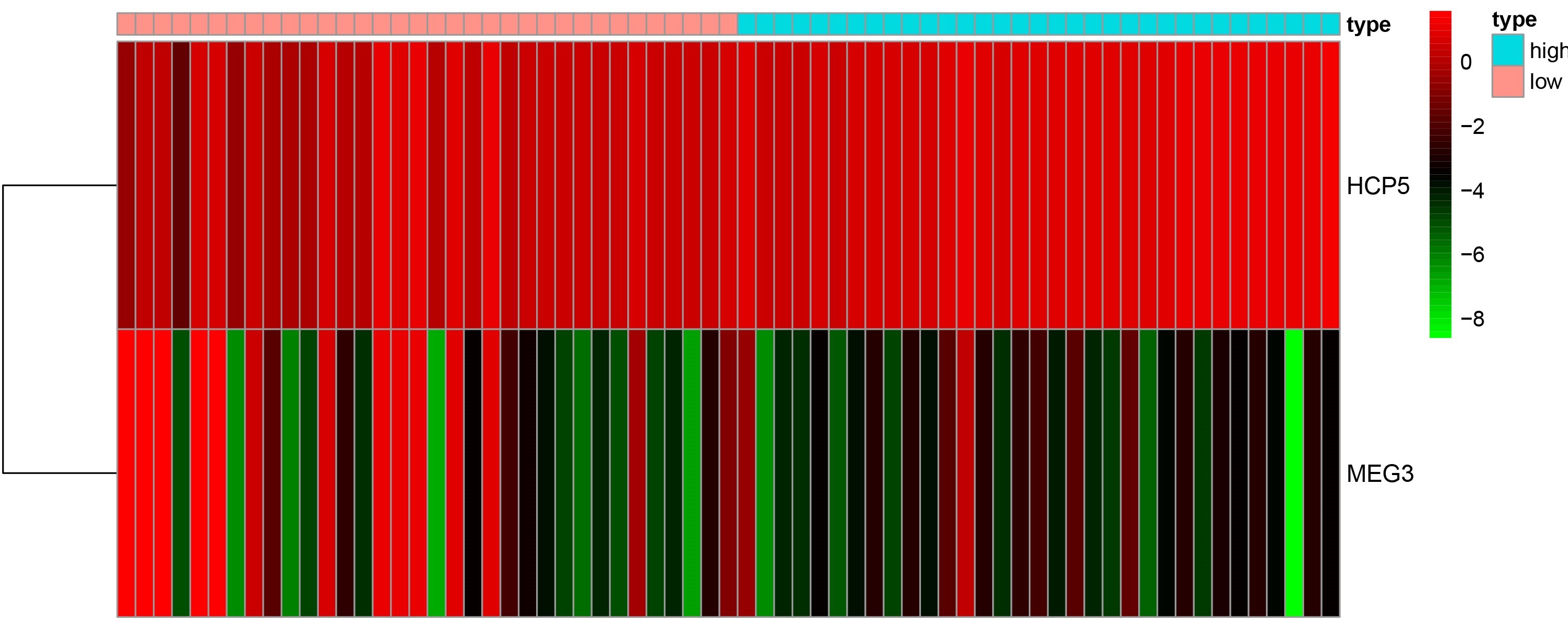

Supplement: Supplementary file 1 [file DataSheet1.zip › Supplementary Material/FIGURE/FIGURE 4/FIGURE 4A_riskTrainheatmap.jpg]

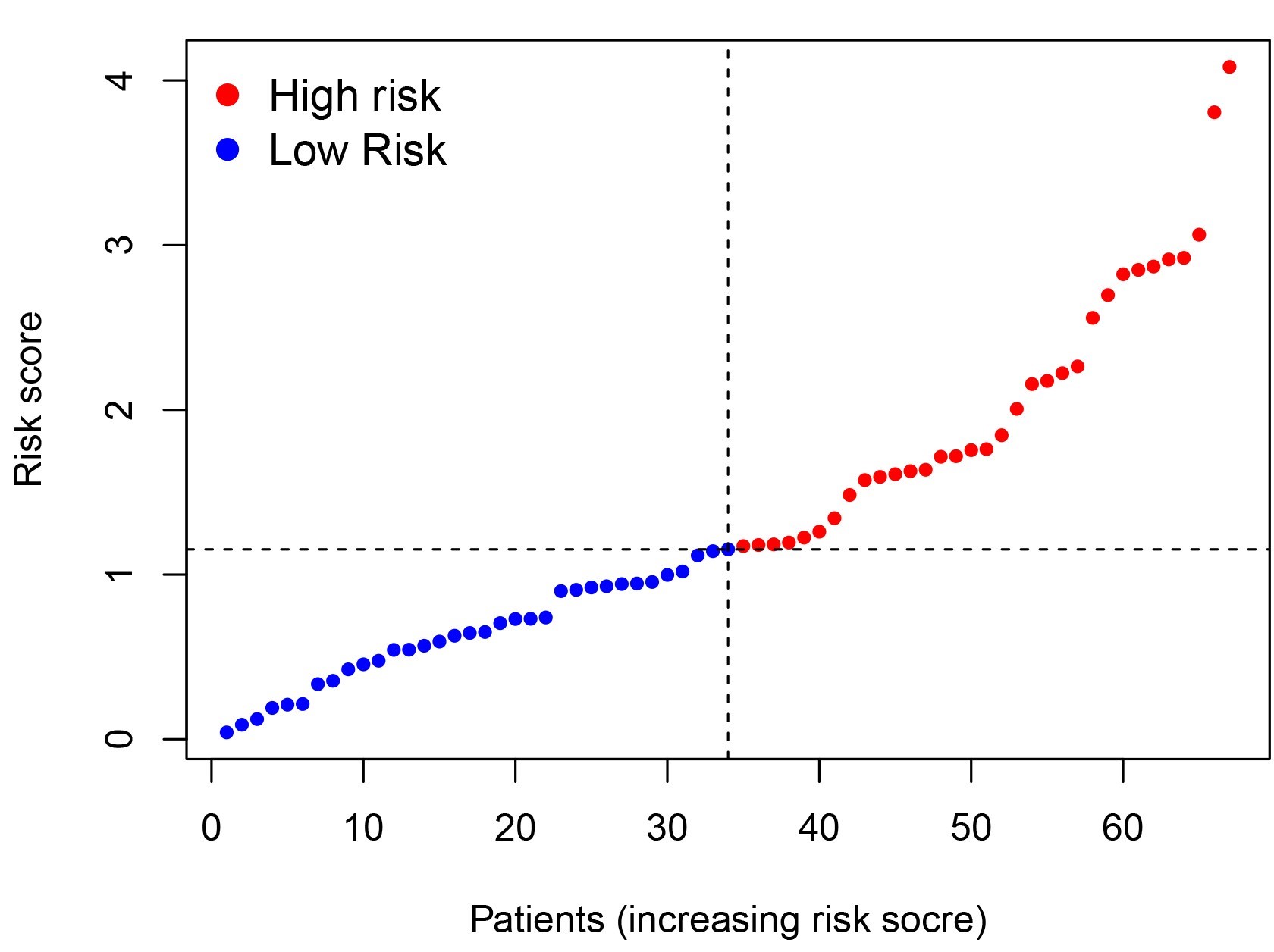

Supplement: Supplementary file 1 [file DataSheet1.zip › Supplementary Material/FIGURE/FIGURE 4/FIGURE 4A_train.riskScore.jpg]

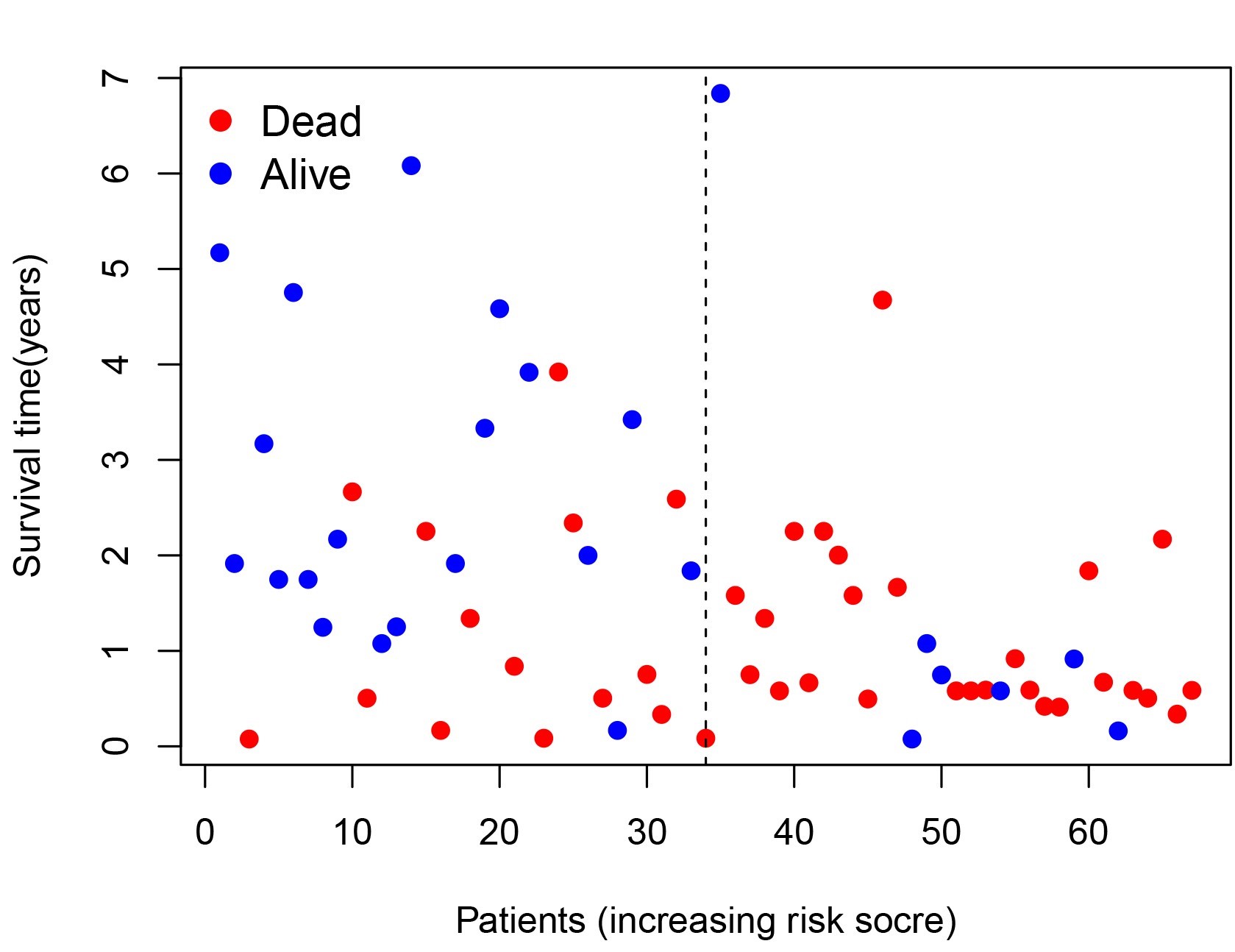

Supplement: Supplementary file 1 [file DataSheet1.zip › Supplementary Material/FIGURE/FIGURE 4/FIGURE 4A_train.survStat.jpg]

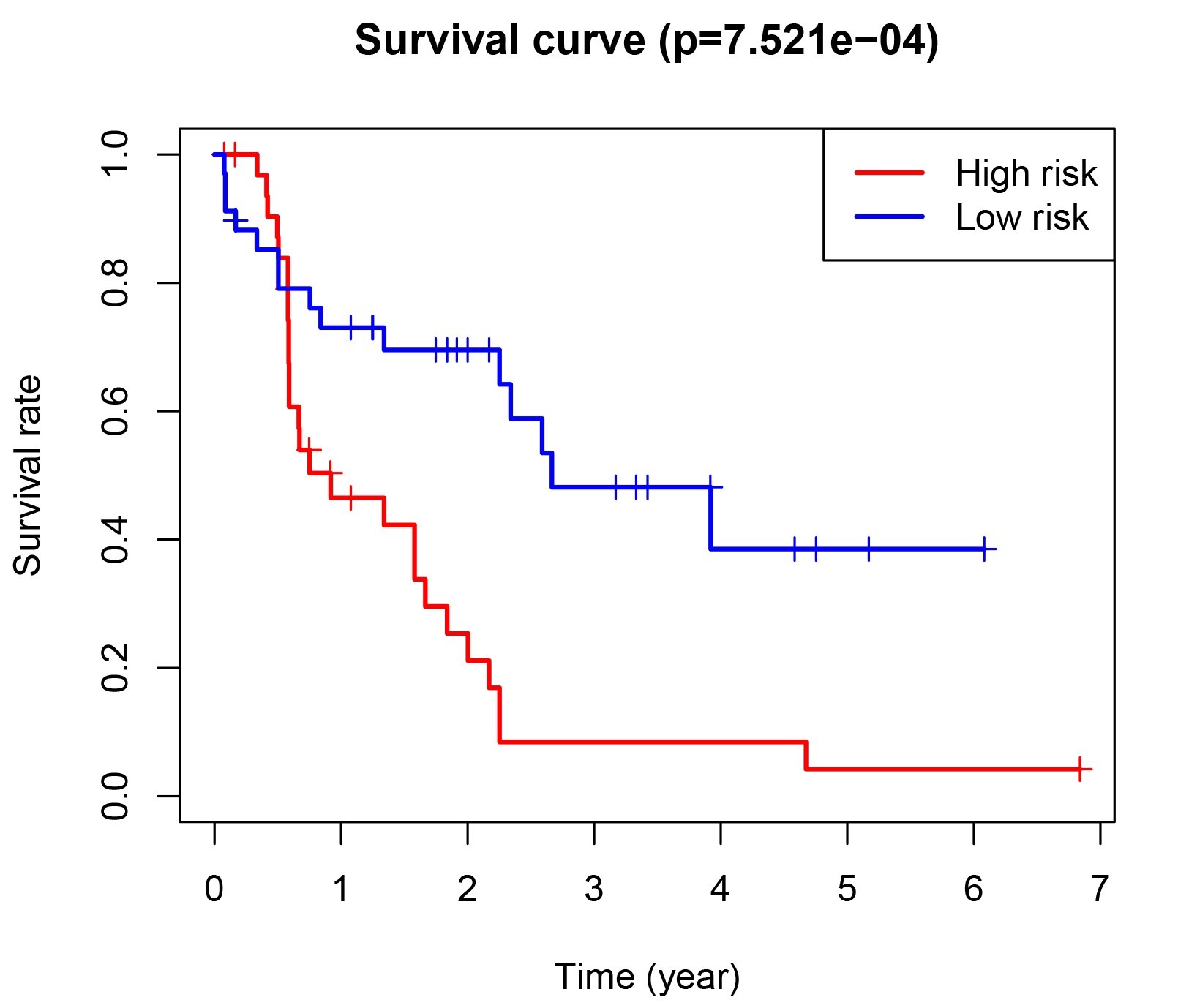

Supplement: Supplementary file 1 [file DataSheet1.zip › Supplementary Material/FIGURE/FIGURE 4/FIGURE 4B_survivalTrain.jpg]

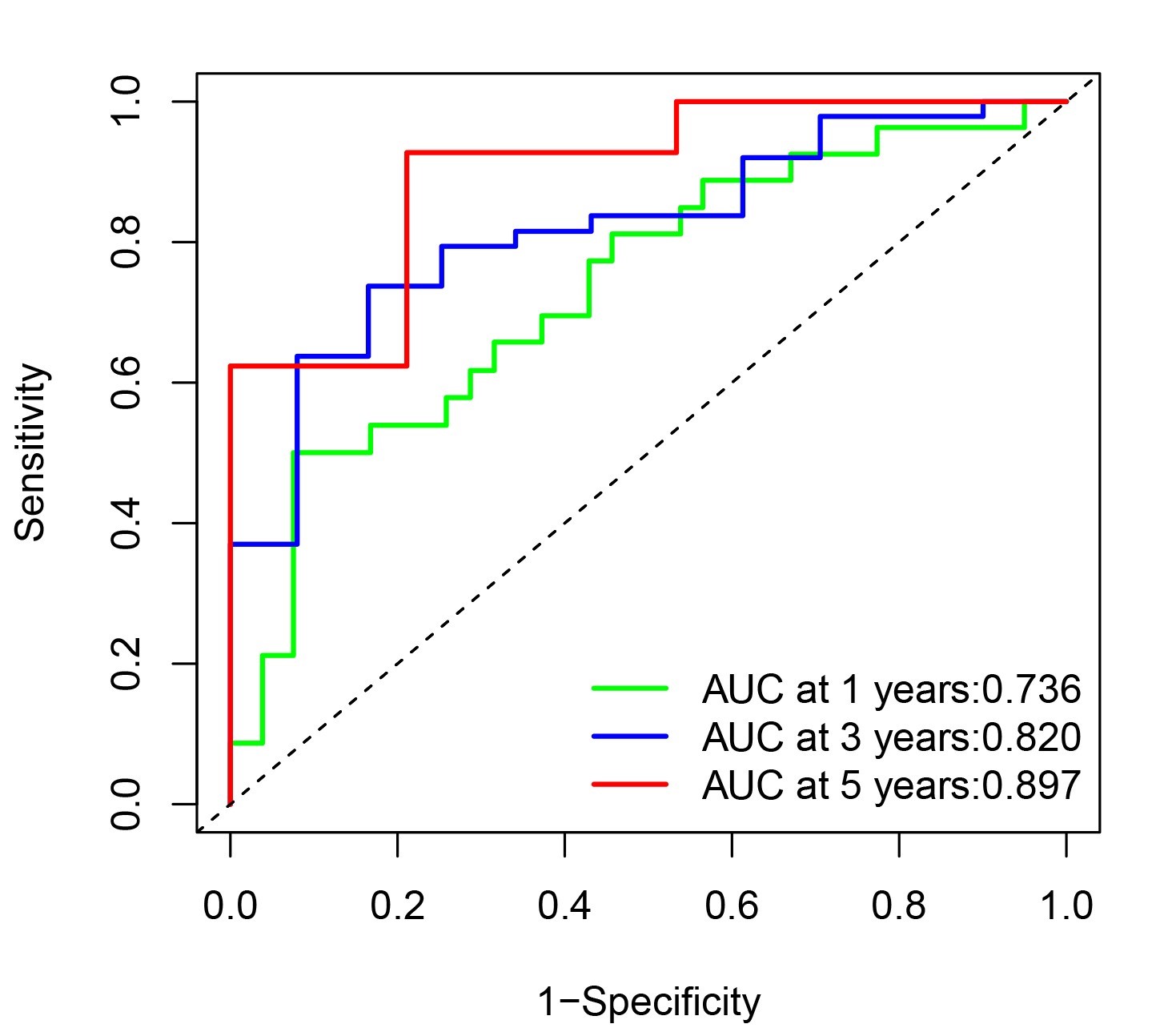

Supplement: Supplementary file 1 [file DataSheet1.zip › Supplementary Material/FIGURE/FIGURE 4/FIGURE 4C_train.ROC.jpg]

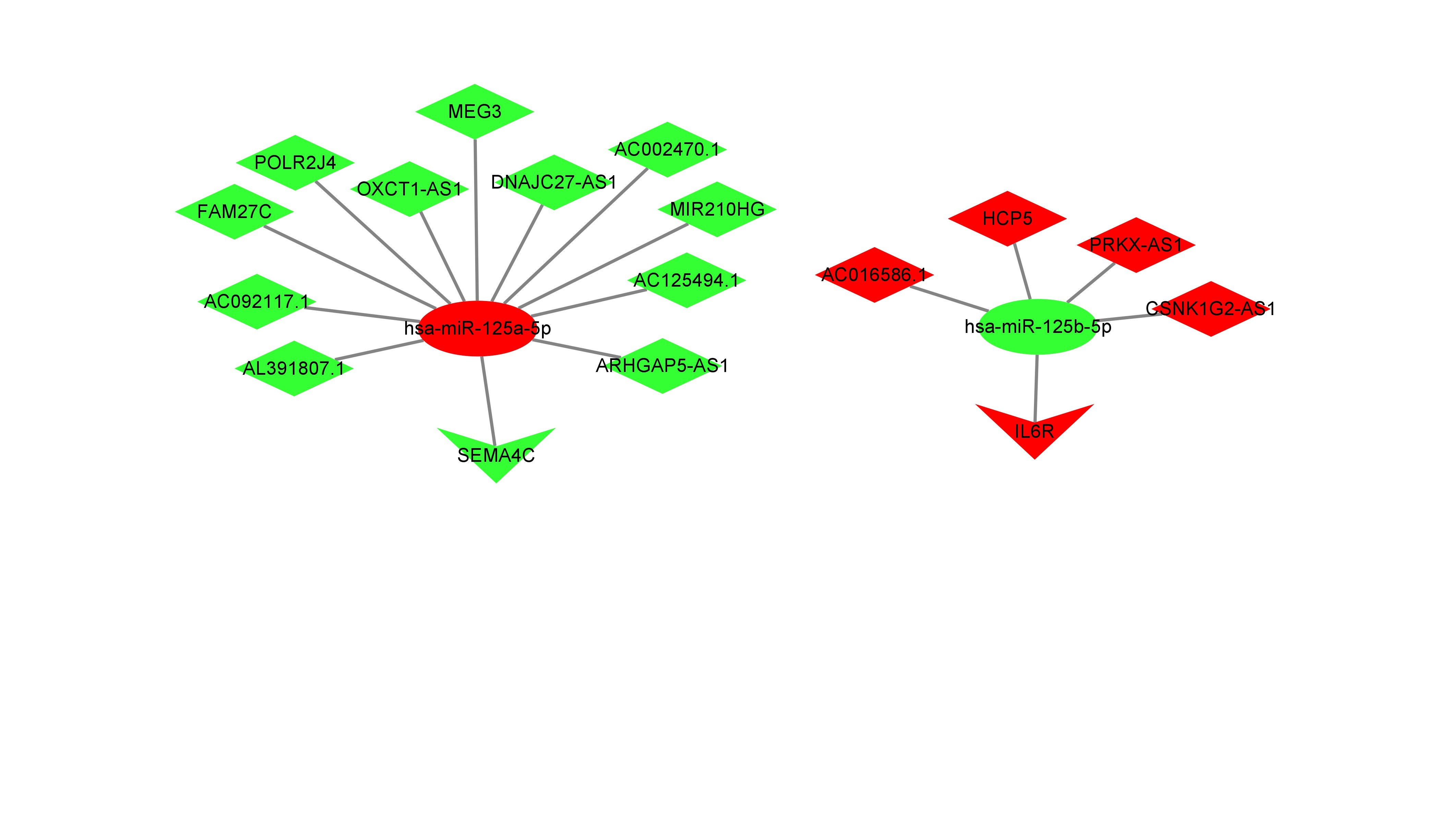

Supplement: Supplementary file 1 [file DataSheet1.zip › Supplementary Material/FIGURE/FIGURE 4/FIGURE 4D.jpg]

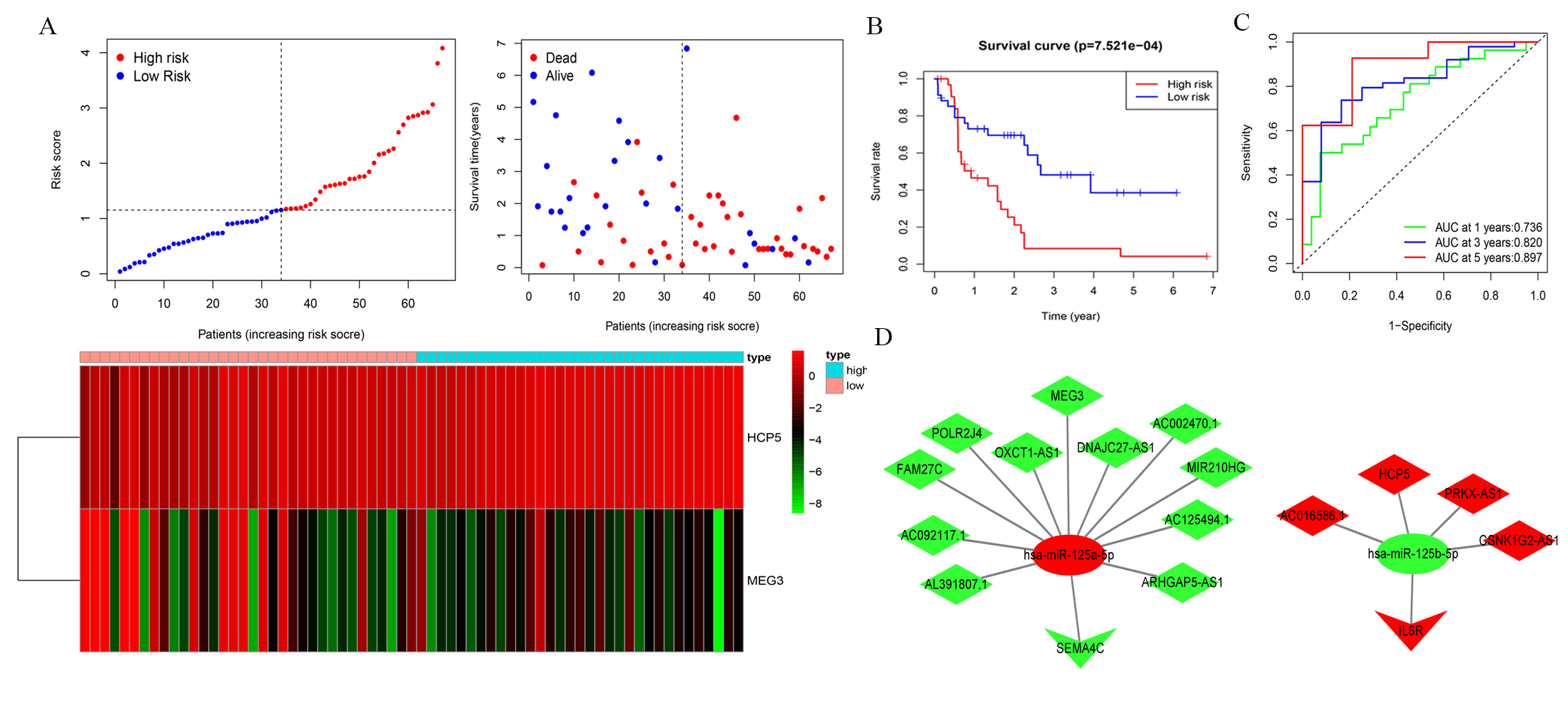

Supplement: Supplementary file 1 [file DataSheet1.zip › Supplementary Material/FIGURE/FIGURE 4.jpg]

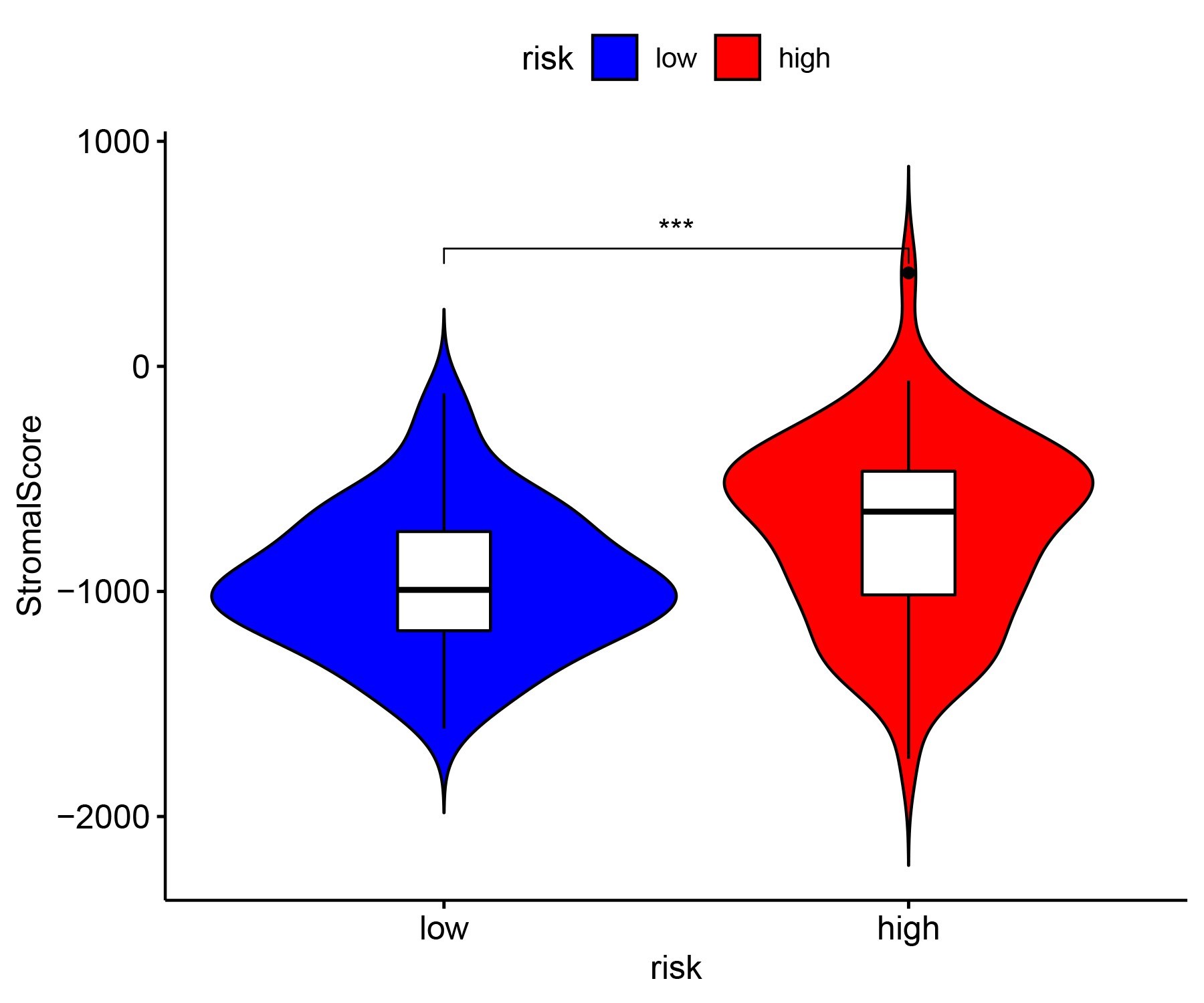

Supplement: Supplementary file 1 [file DataSheet1.zip › Supplementary Material/FIGURE/FIGURE 5/FIGURE 5A_vioplot_StromalScore.jpg]

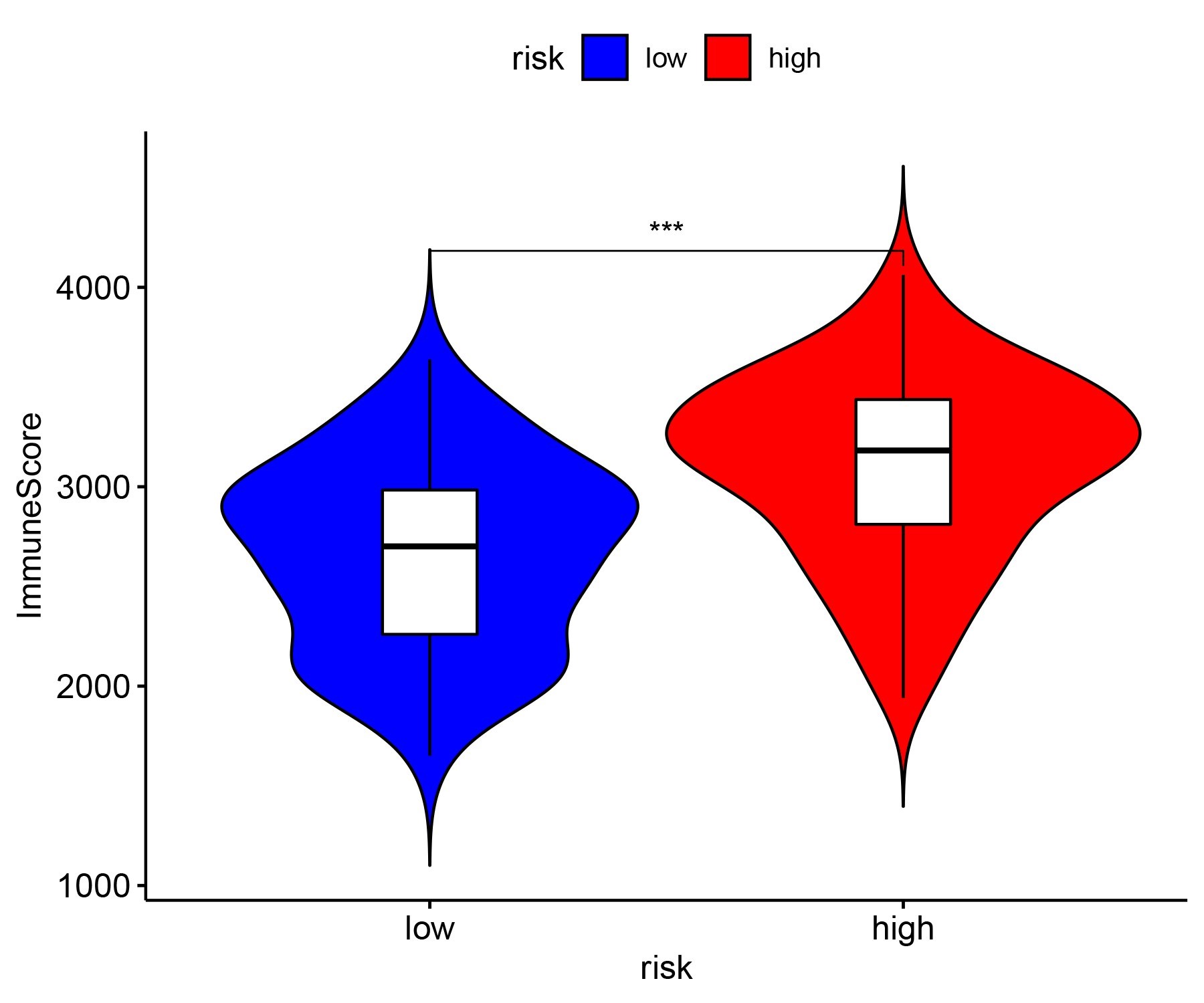

Supplement: Supplementary file 1 [file DataSheet1.zip › Supplementary Material/FIGURE/FIGURE 5/FIGURE 5B_vioplot_ImmuneScore.jpg]

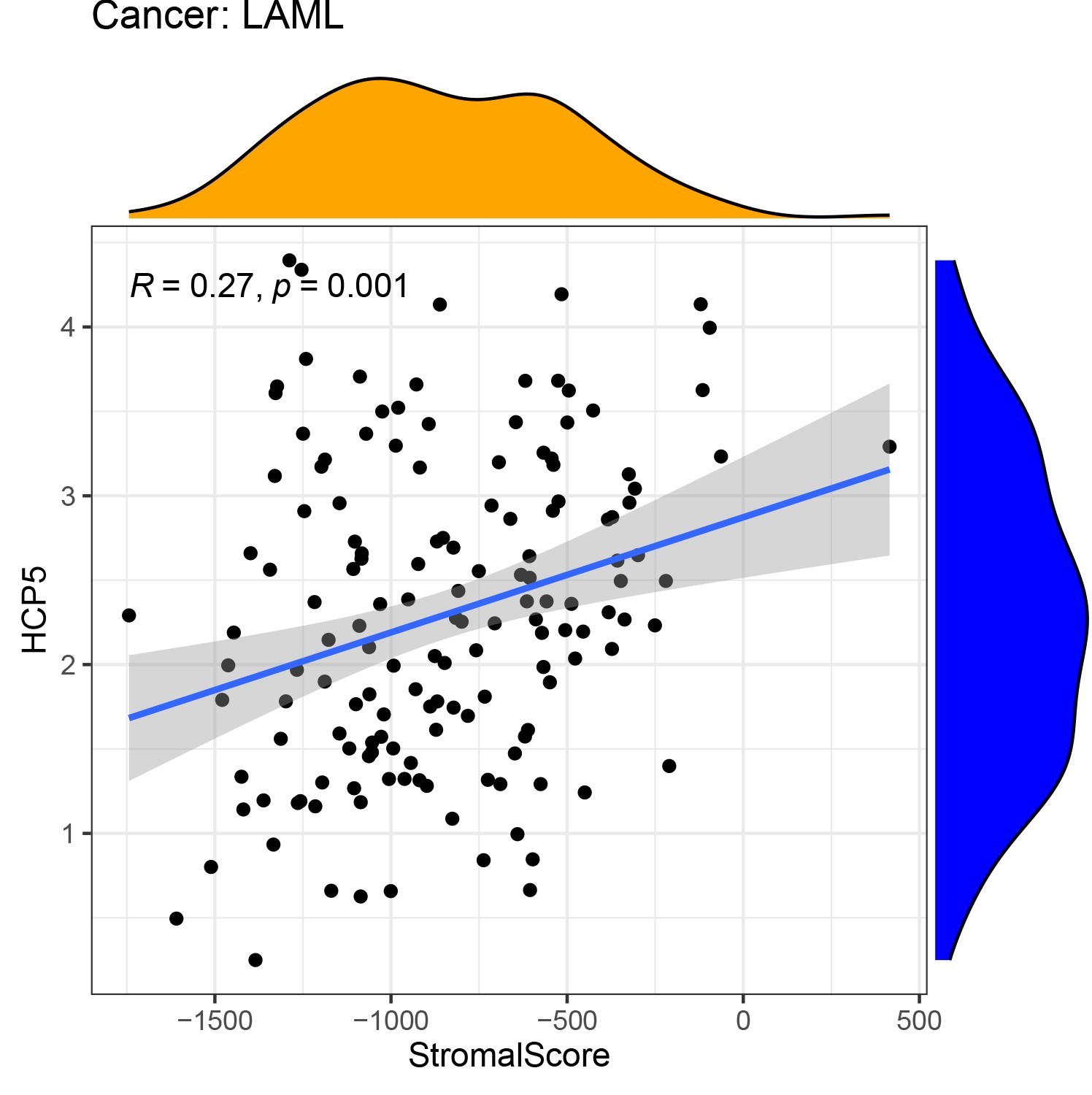

Supplement: Supplementary file 1 [file DataSheet1.zip › Supplementary Material/FIGURE/FIGURE 5/FIGURE 5C_estimateCor.HCP5_StromalScore.jpg]

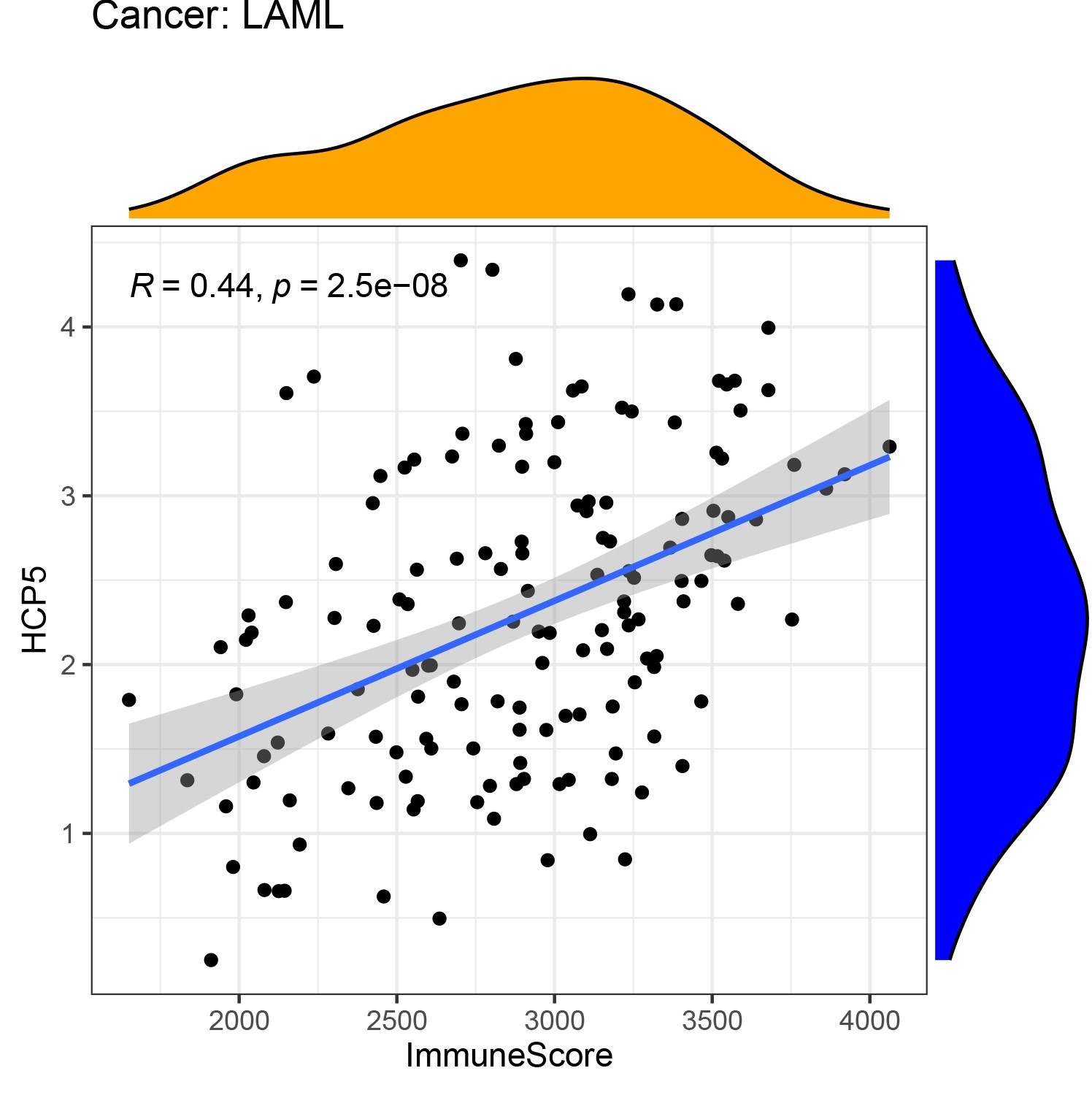

Supplement: Supplementary file 1 [file DataSheet1.zip › Supplementary Material/FIGURE/FIGURE 5/FIGURE 5D_estimateCor.HCP5_ImmuneScore.jpg]

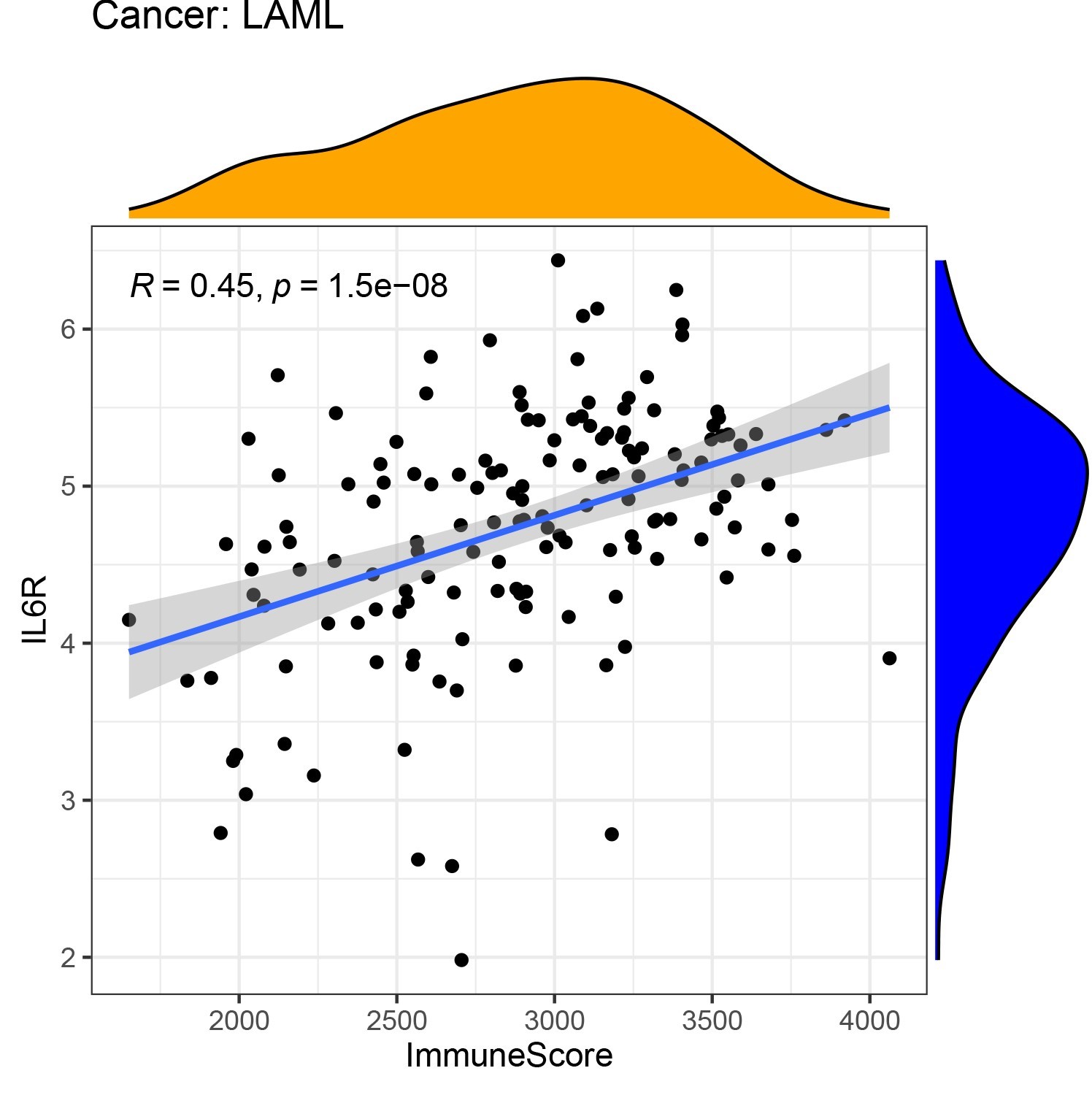

Supplement: Supplementary file 1 [file DataSheet1.zip › Supplementary Material/FIGURE/FIGURE 5/FIGURE 5E_estimateCor.IL6R_ImmuneScore.jpg]

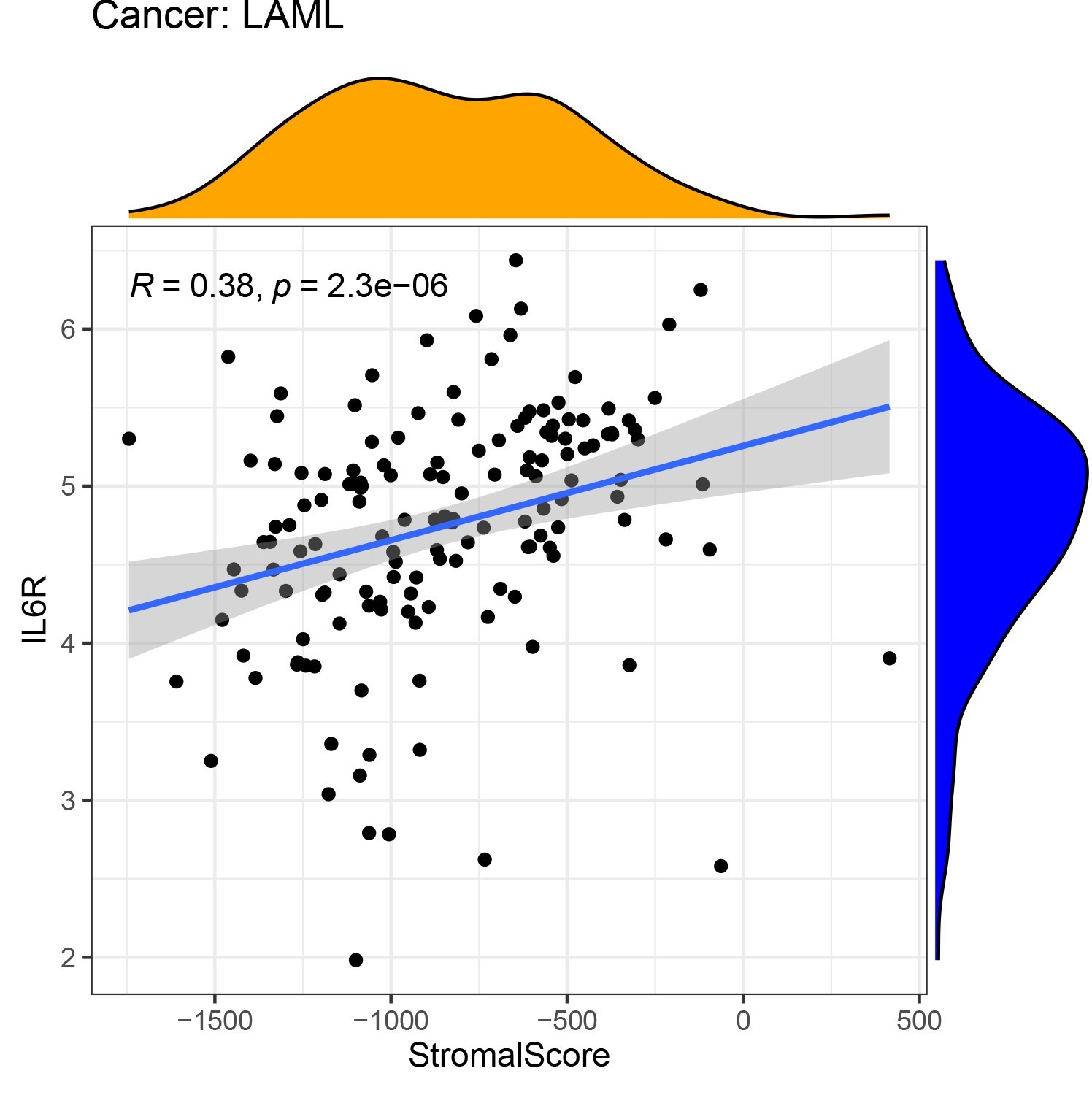

Supplement: Supplementary file 1 [file DataSheet1.zip › Supplementary Material/FIGURE/FIGURE 5/FIGURE 5F_estimateCor.IL6R_StromalScore.jpg]

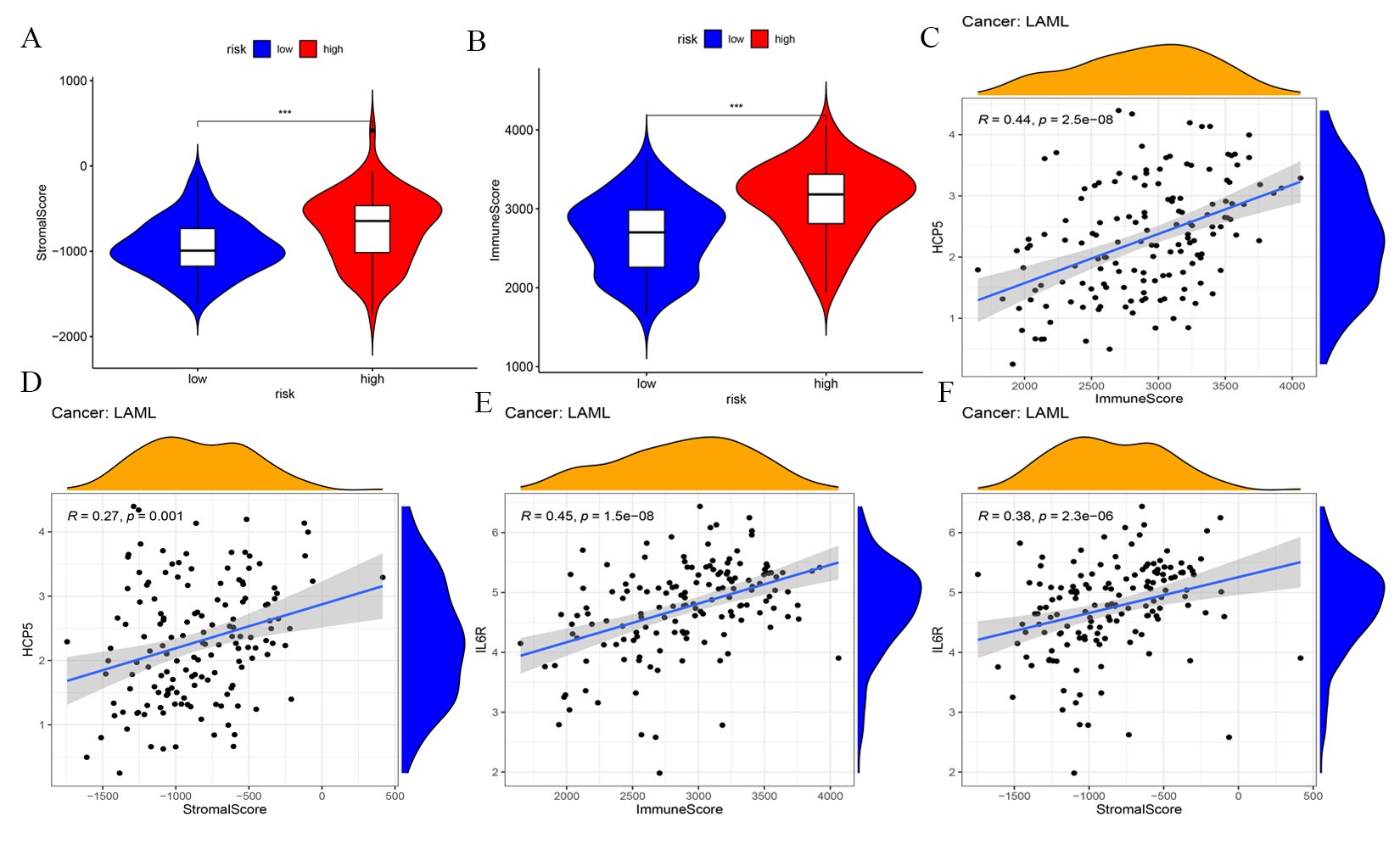

Supplement: Supplementary file 1 [file DataSheet1.zip › Supplementary Material/FIGURE/FIGURE 5.jpg]

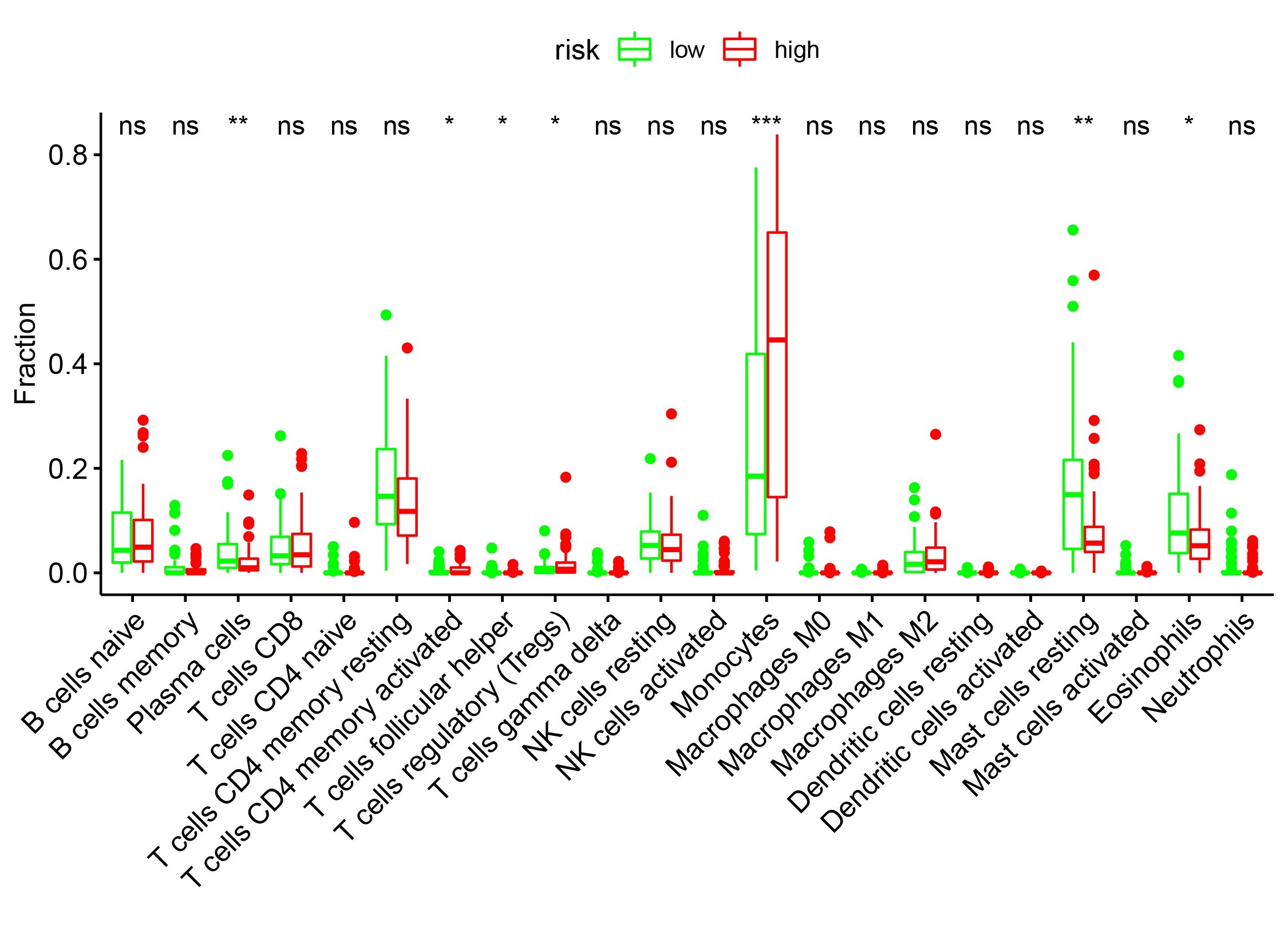

Supplement: Supplementary file 1 [file DataSheet1.zip › Supplementary Material/FIGURE/FIGURE 6/FIGURE 6A_boxplot.jpg]

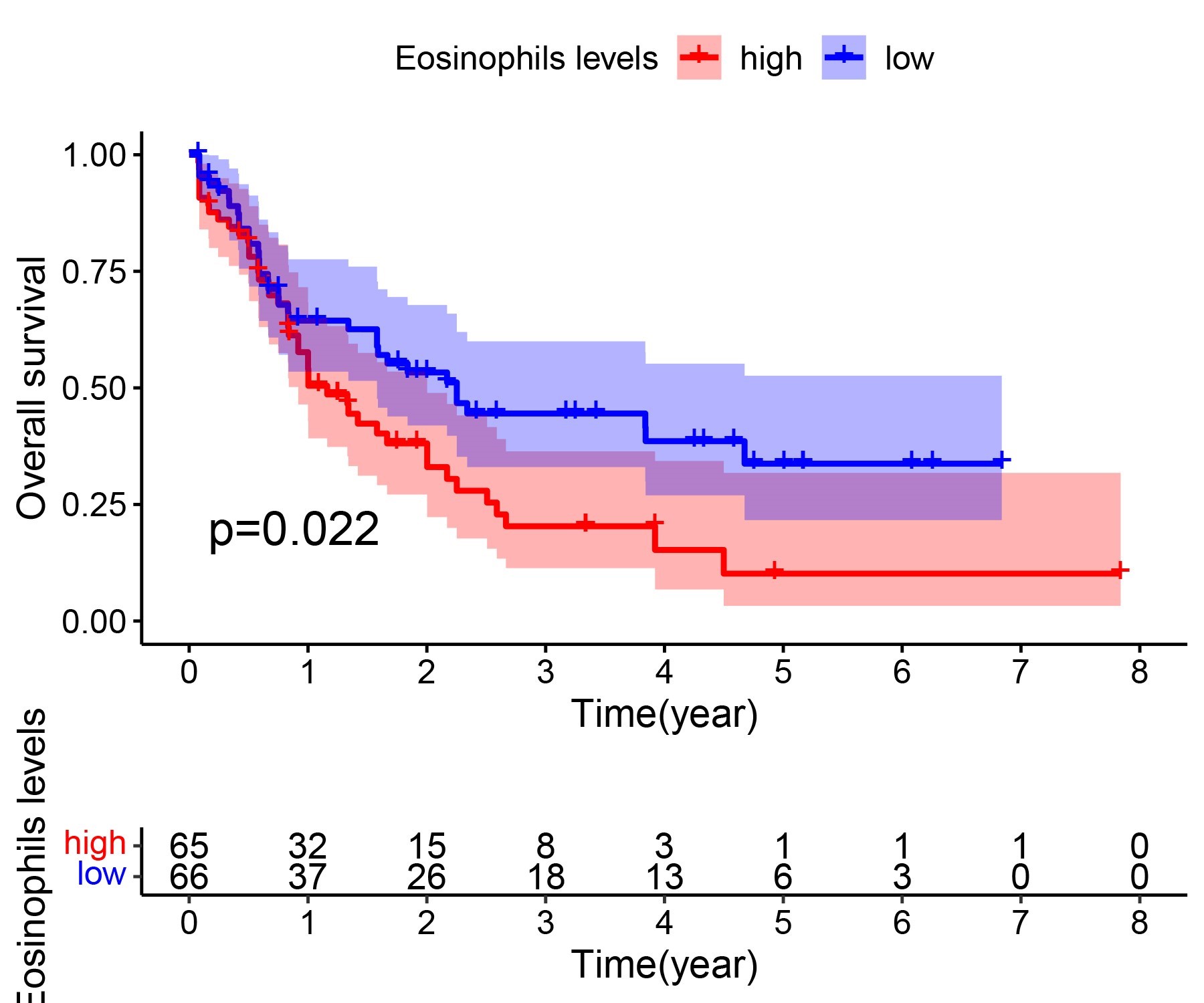

Supplement: Supplementary file 1 [file DataSheet1.zip › Supplementary Material/FIGURE/FIGURE 6/FIGURE 6B_Eosinophils.jpg]

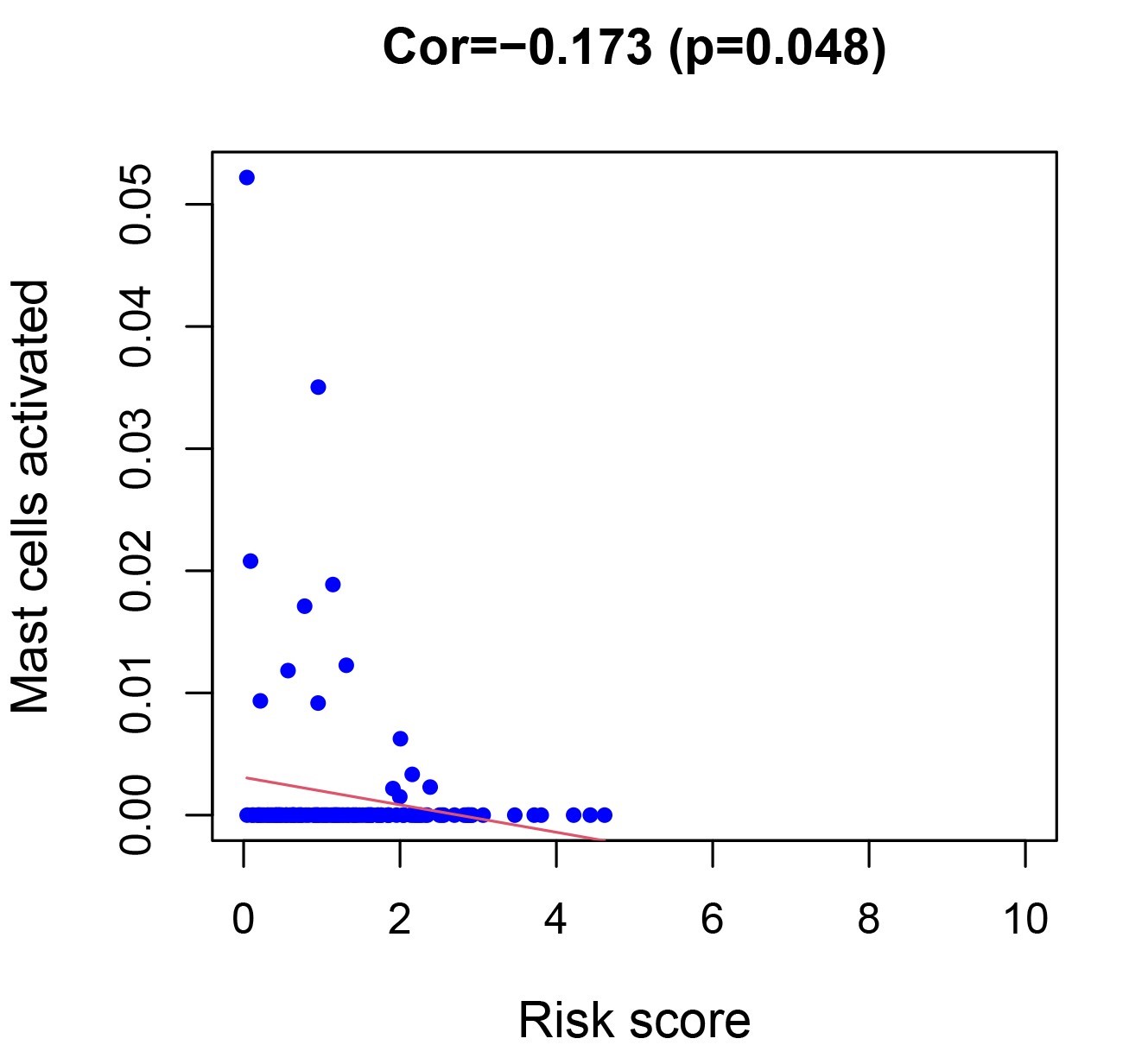

Supplement: Supplementary file 1 [file DataSheet1.zip › Supplementary Material/FIGURE/FIGURE 6/FIGURE 6B_Mast-cells-activated.jpg]

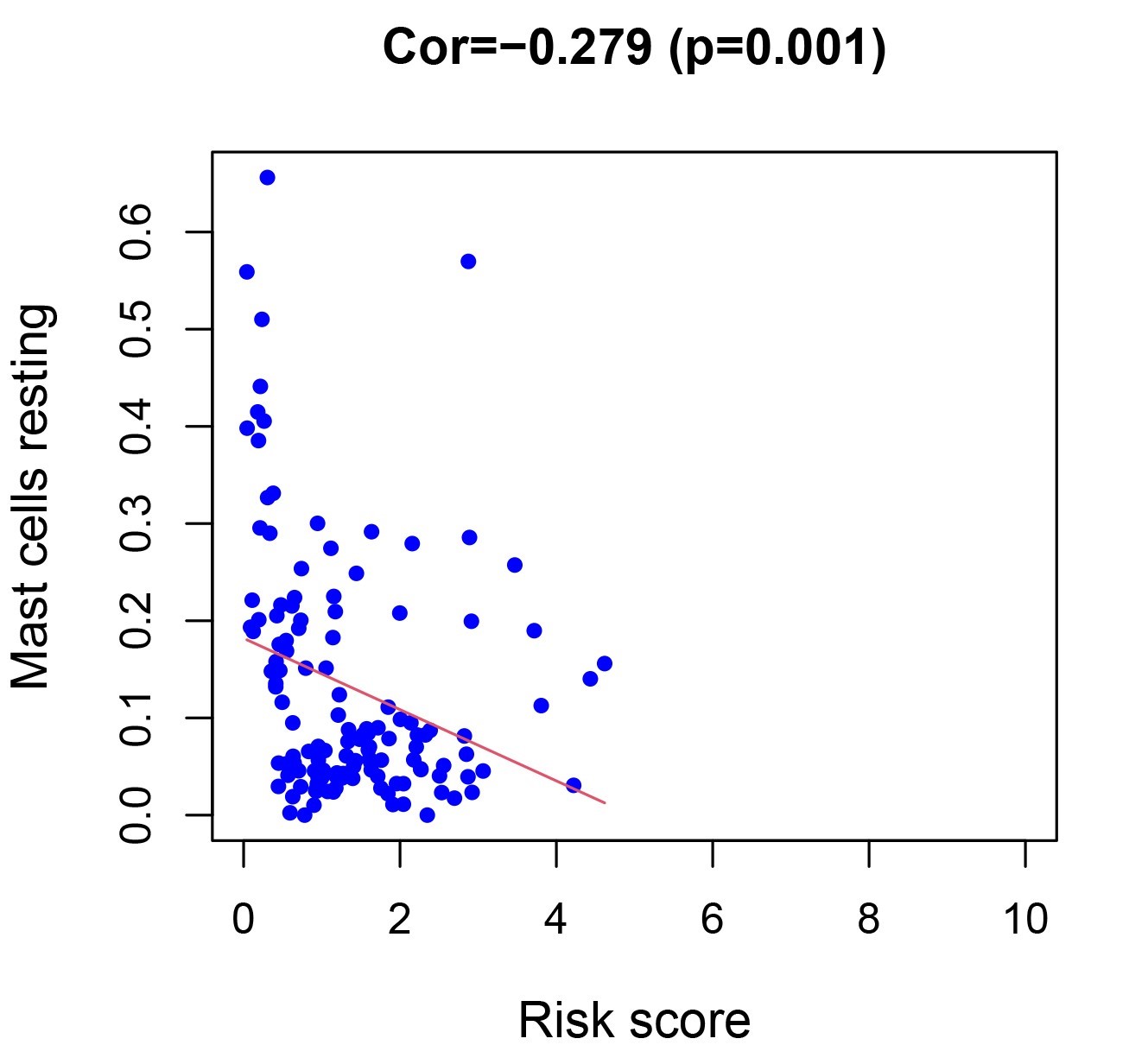

Supplement: Supplementary file 1 [file DataSheet1.zip › Supplementary Material/FIGURE/FIGURE 6/FIGURE 6B_Mast-cells-resting.jpg]

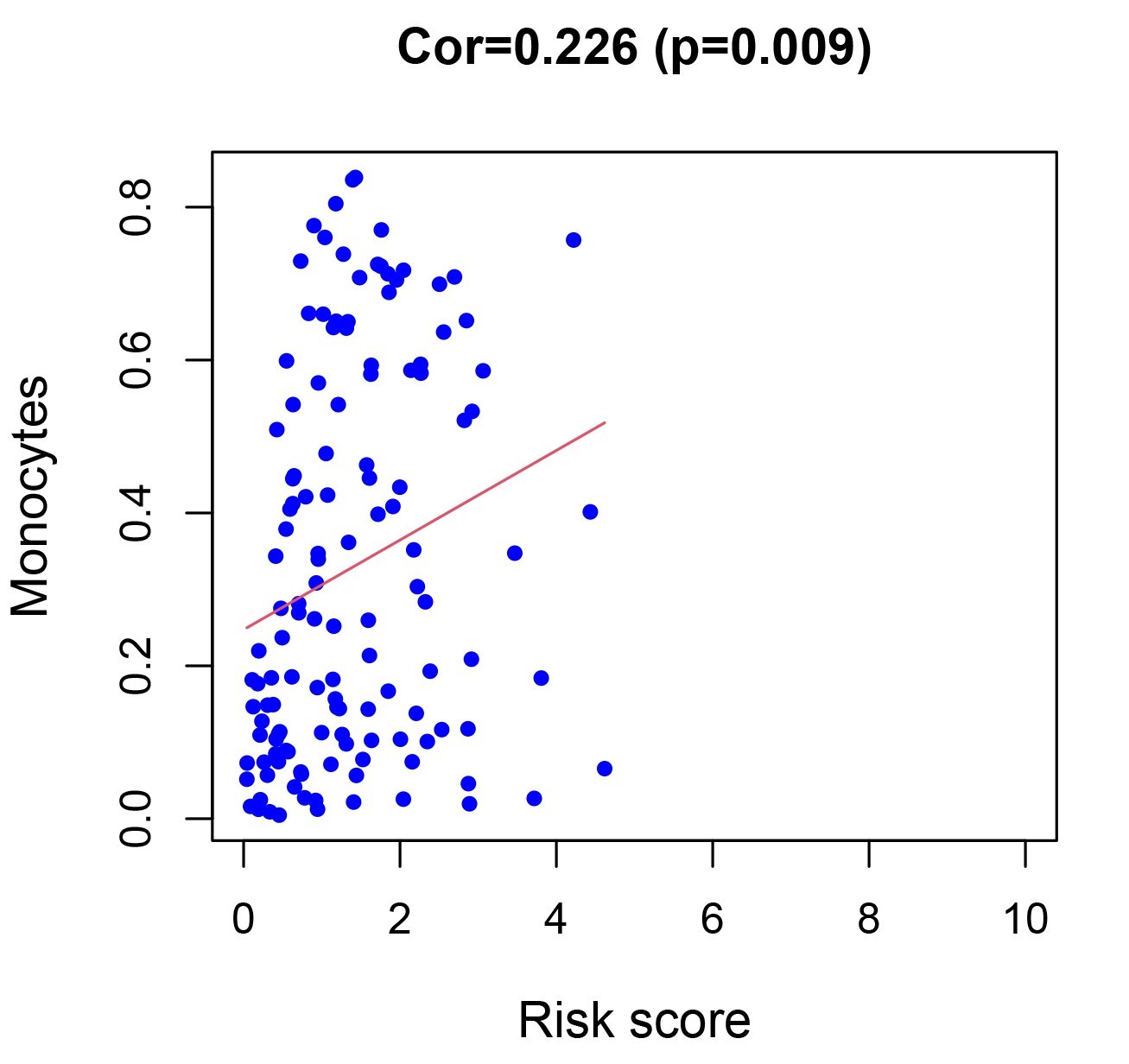

Supplement: Supplementary file 1 [file DataSheet1.zip › Supplementary Material/FIGURE/FIGURE 6/FIGURE 6B_Monocytes.jpg]

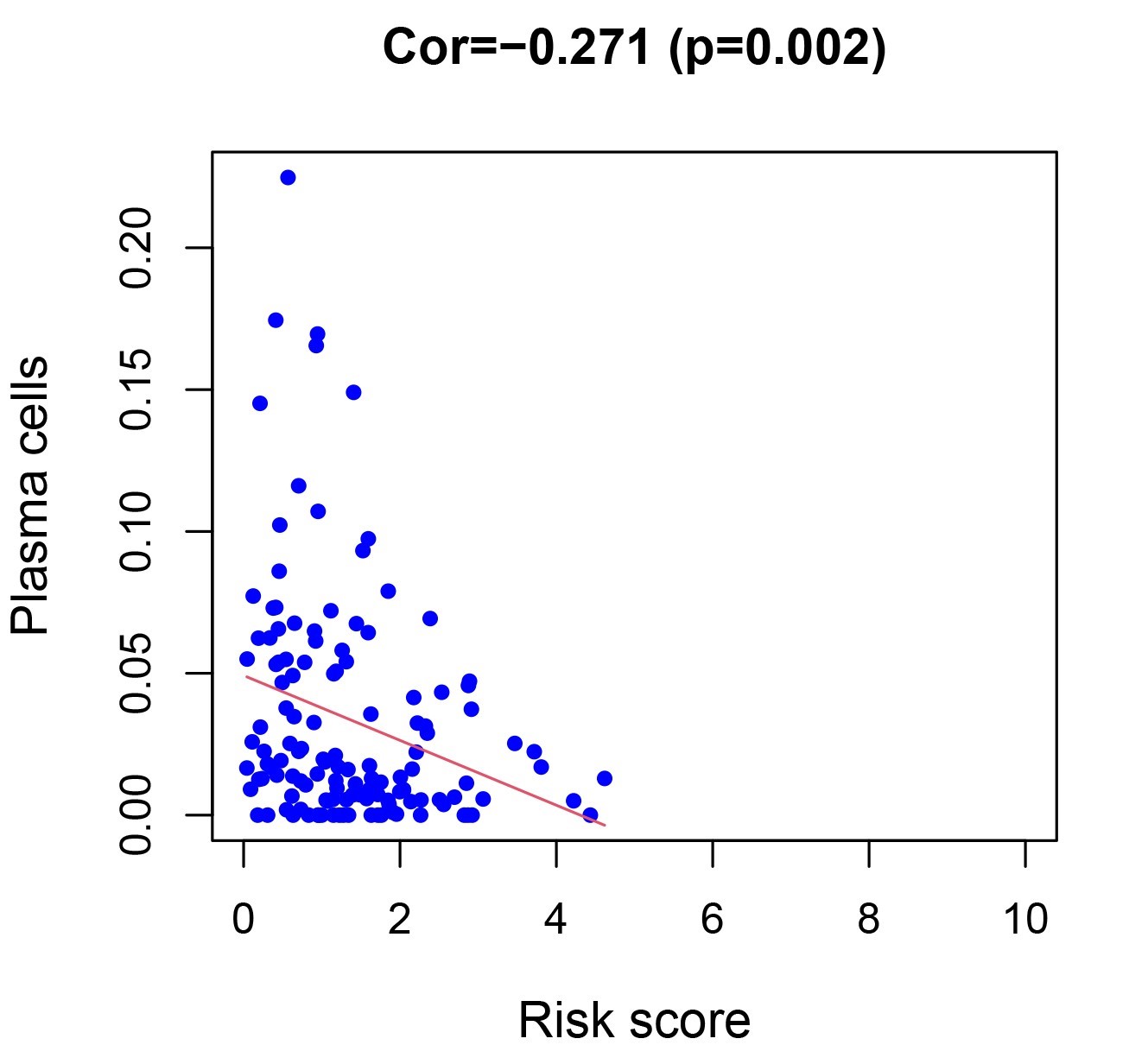

Supplement: Supplementary file 1 [file DataSheet1.zip › Supplementary Material/FIGURE/FIGURE 6/FIGURE 6B_Plasma-cells.jpg]

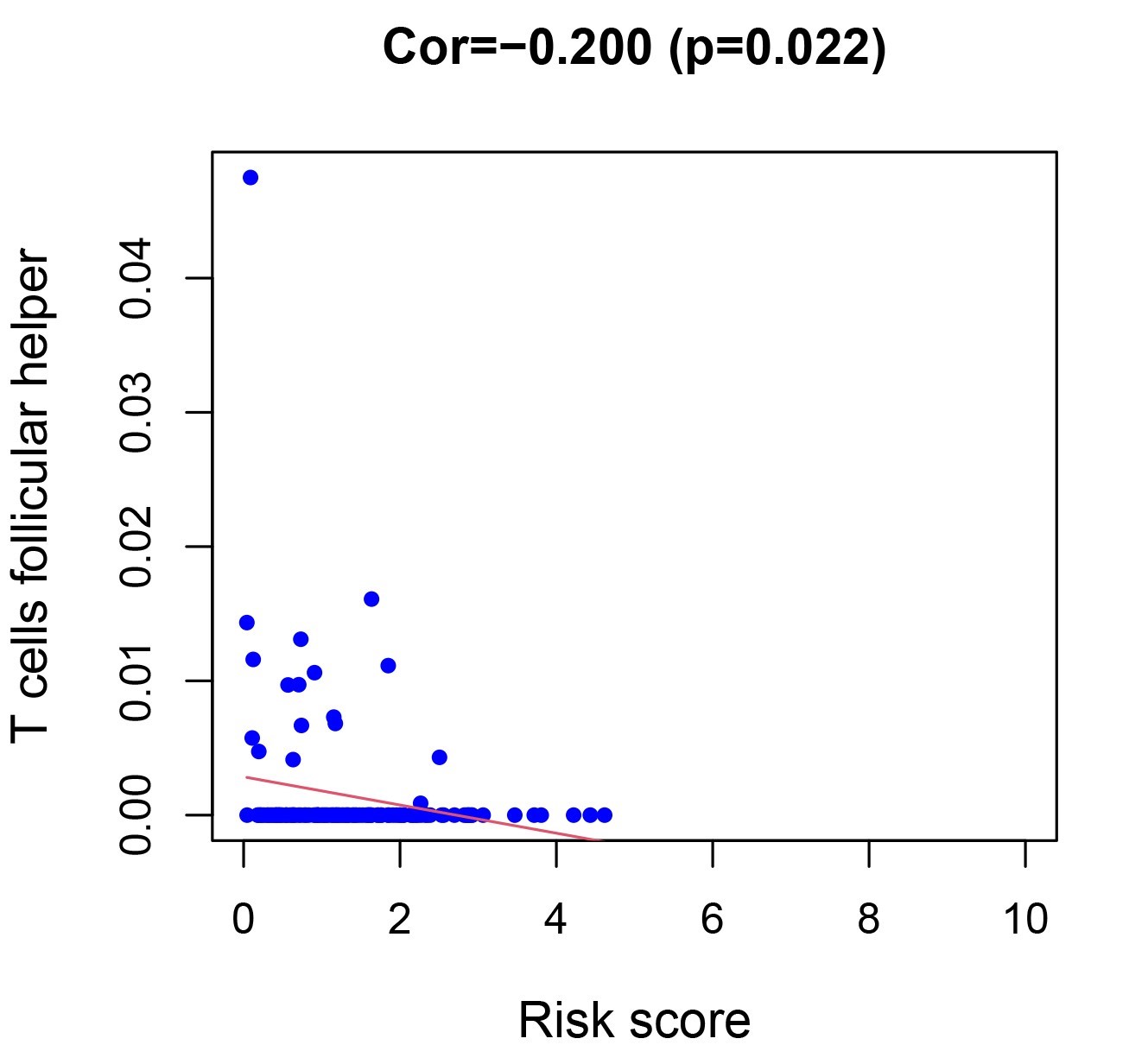

Supplement: Supplementary file 1 [file DataSheet1.zip › Supplementary Material/FIGURE/FIGURE 6/FIGURE 6B_T-cells-follicular-helper.jpg]

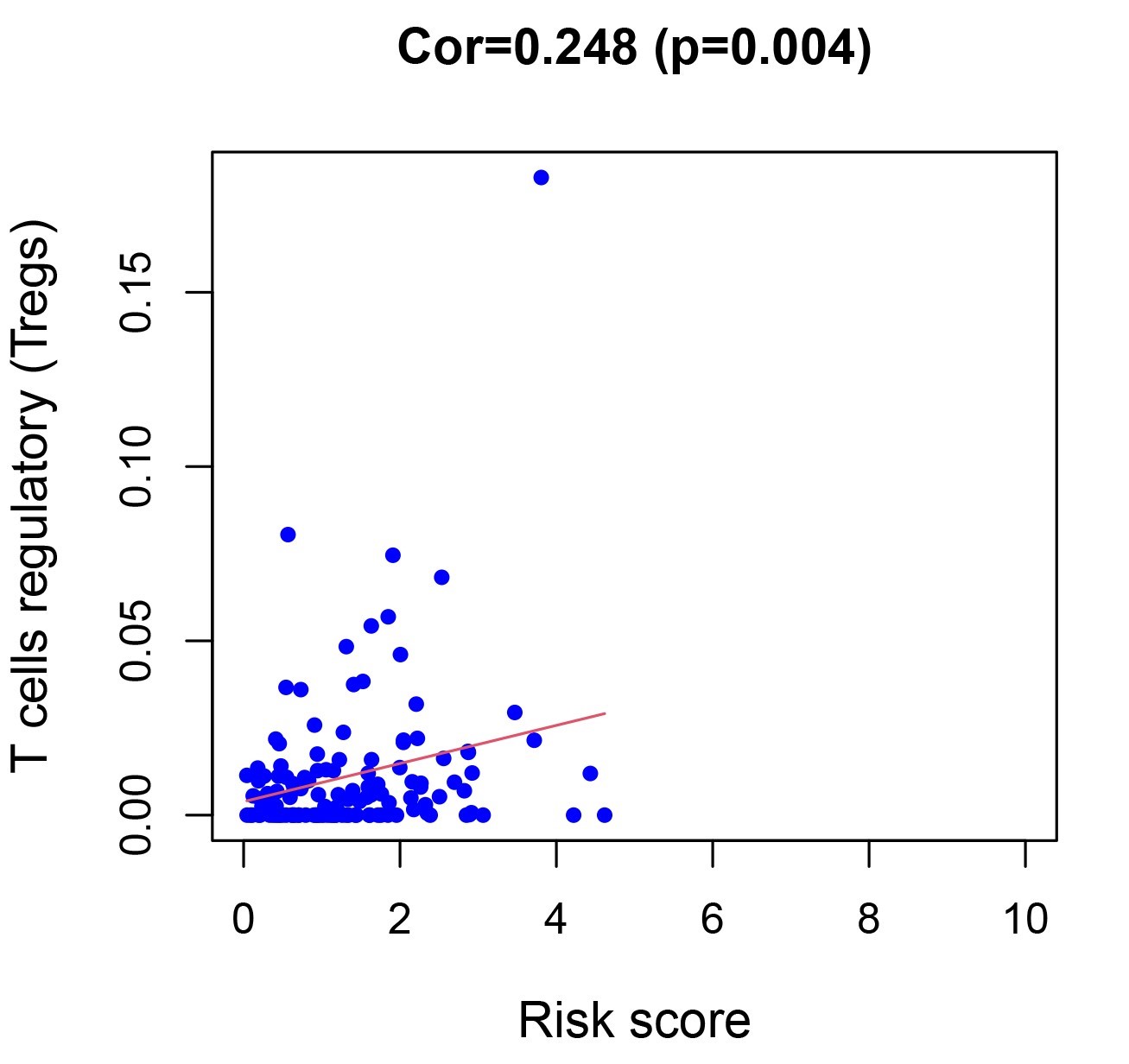

Supplement: Supplementary file 1 [file DataSheet1.zip › Supplementary Material/FIGURE/FIGURE 6/FIGURE 6B_T-cells-regulatory-(Tregs).jpg]

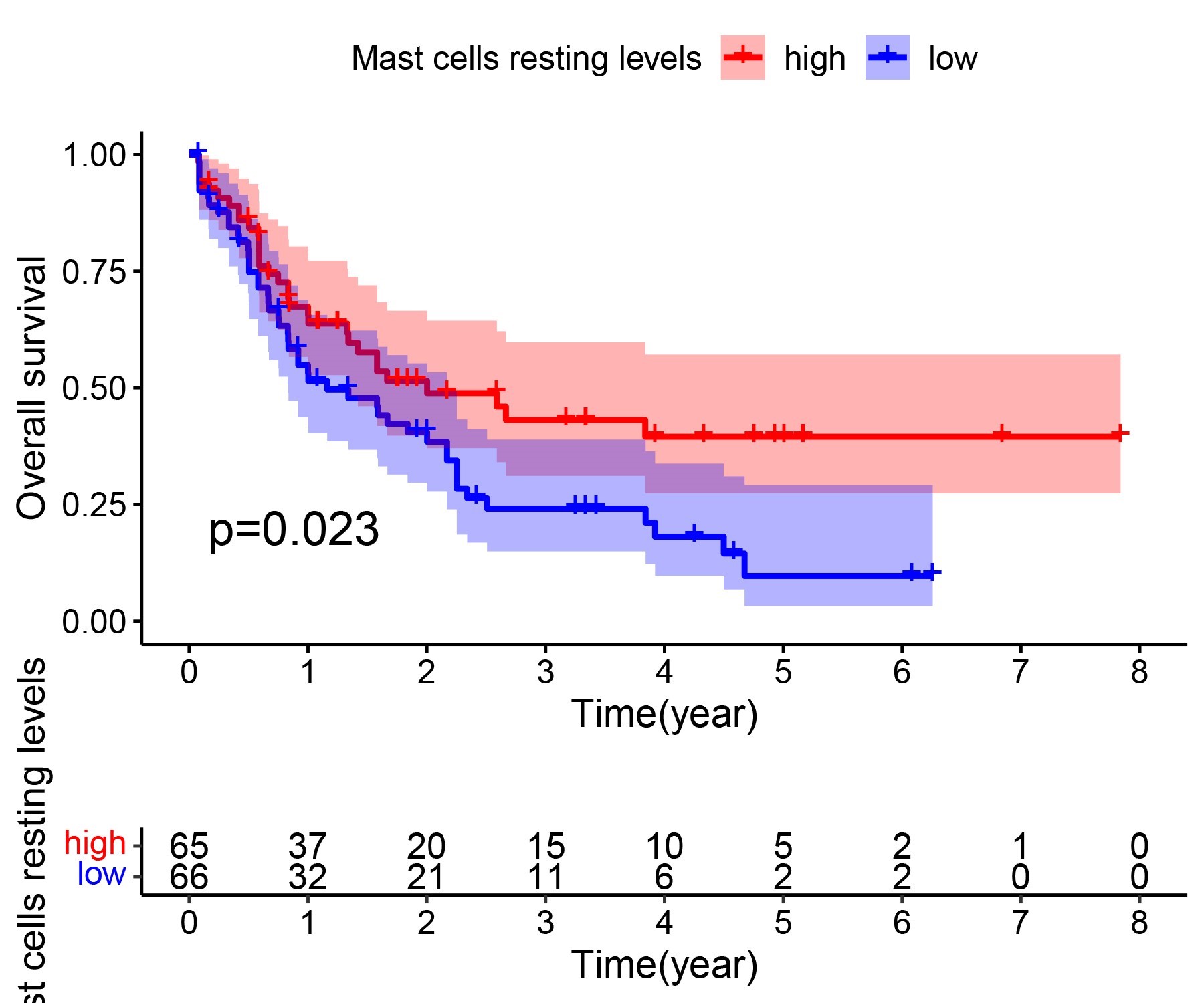

Supplement: Supplementary file 1 [file DataSheet1.zip › Supplementary Material/FIGURE/FIGURE 6/FIGURE 6D_Mast-cells-resting.jpg]

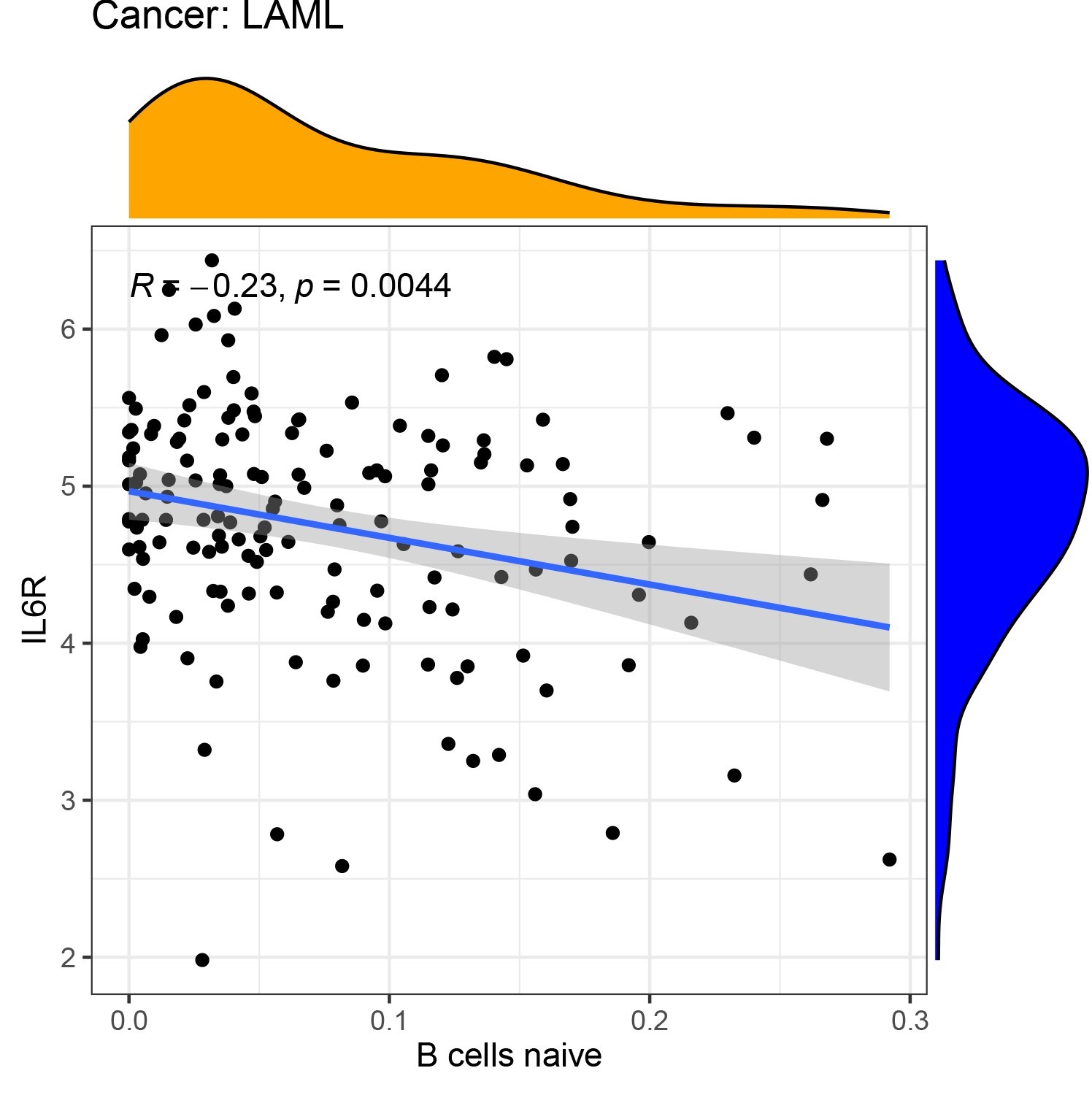

Supplement: Supplementary file 1 [file DataSheet1.zip › Supplementary Material/FIGURE/FIGURE 6/FIGURE 6E_estimateCor.IL6R_B-cells-naive.jpg]

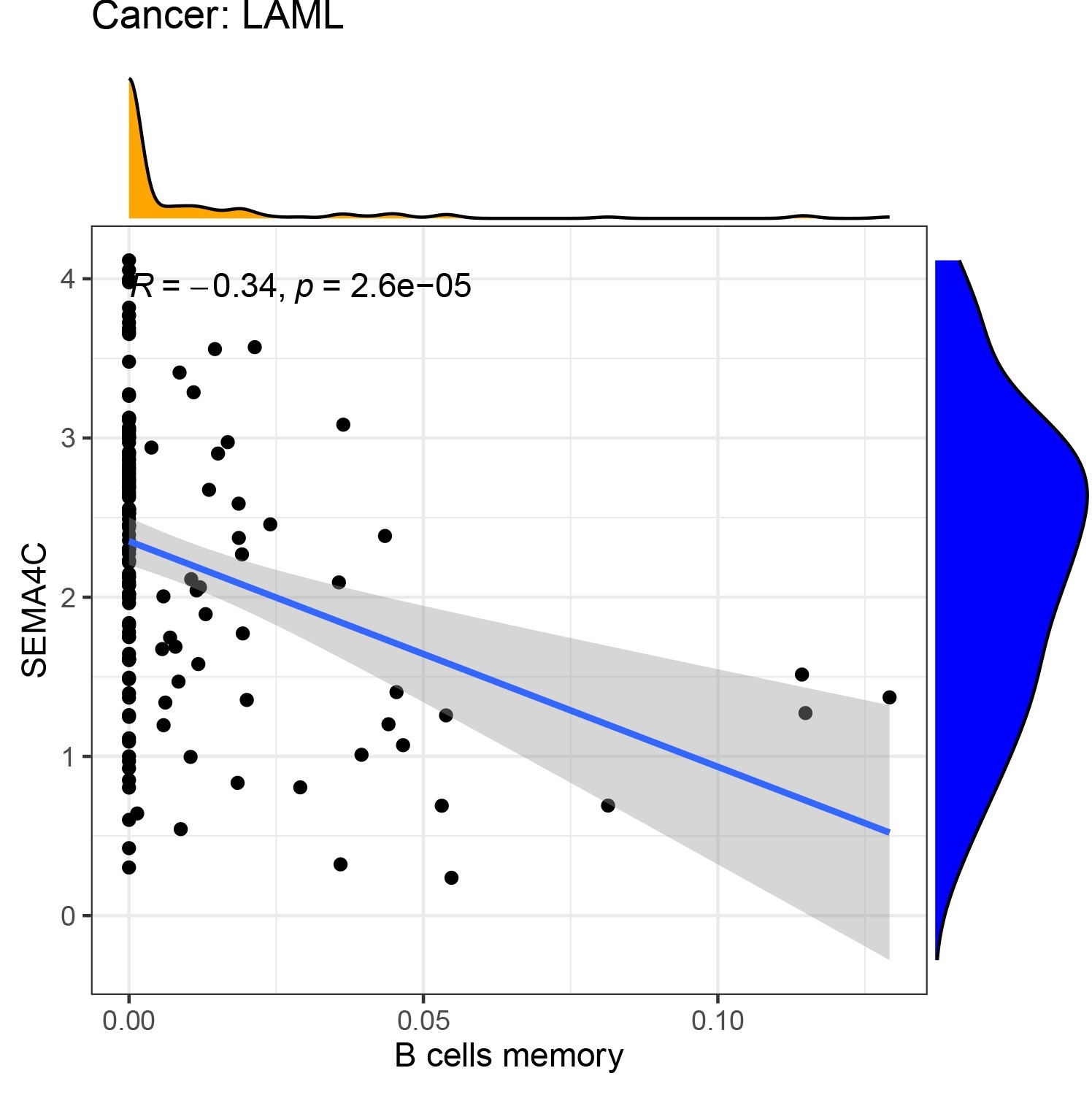

Supplement: Supplementary file 1 [file DataSheet1.zip › Supplementary Material/FIGURE/FIGURE 6/FIGURE 6F_estimateCor.SEMA4C_B-cells-memory.jpg]

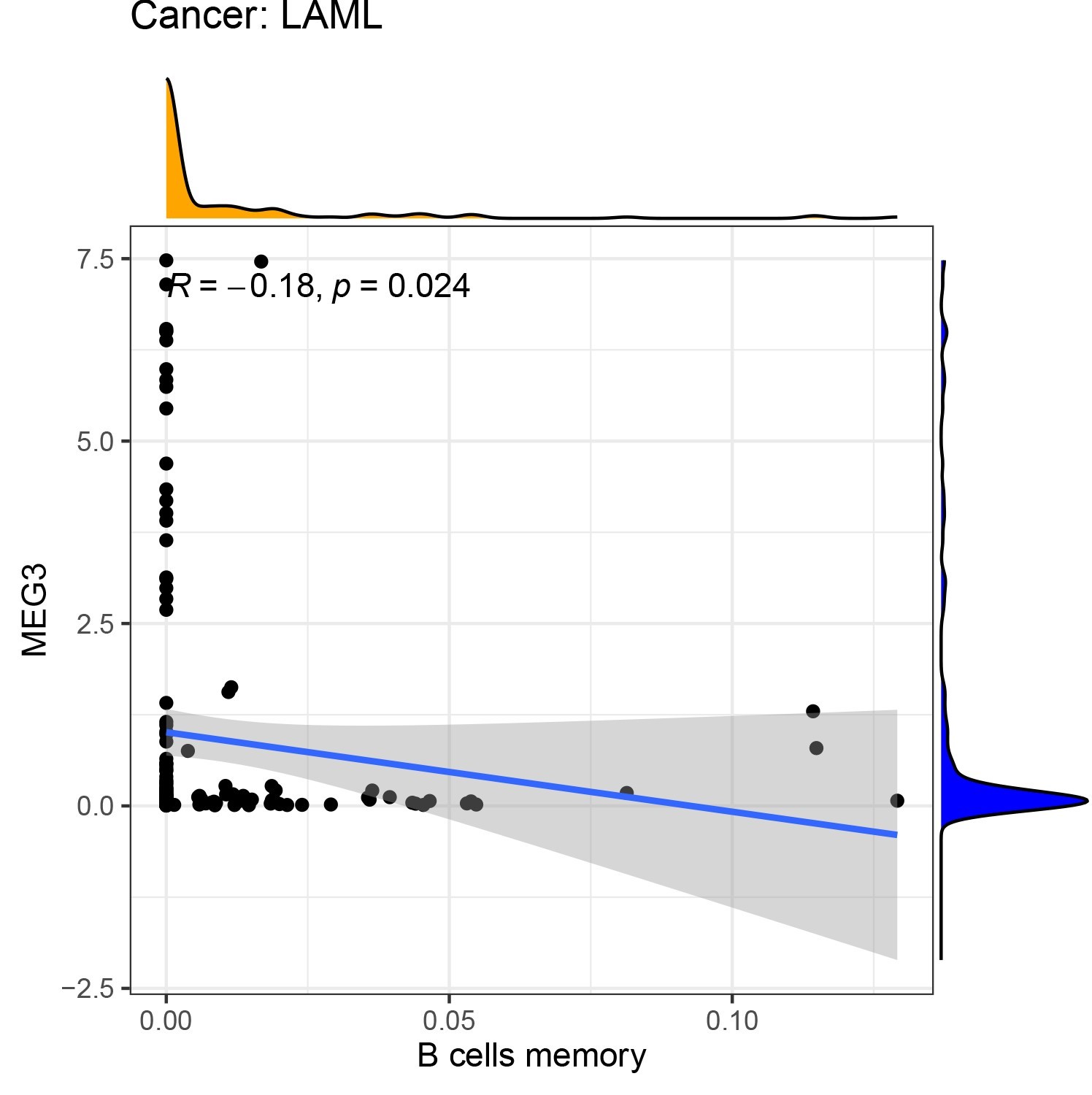

Supplement: Supplementary file 1 [file DataSheet1.zip › Supplementary Material/FIGURE/FIGURE 6/FIGURE 6G_estimateCor.MEG3_B-cells-memory.jpg]

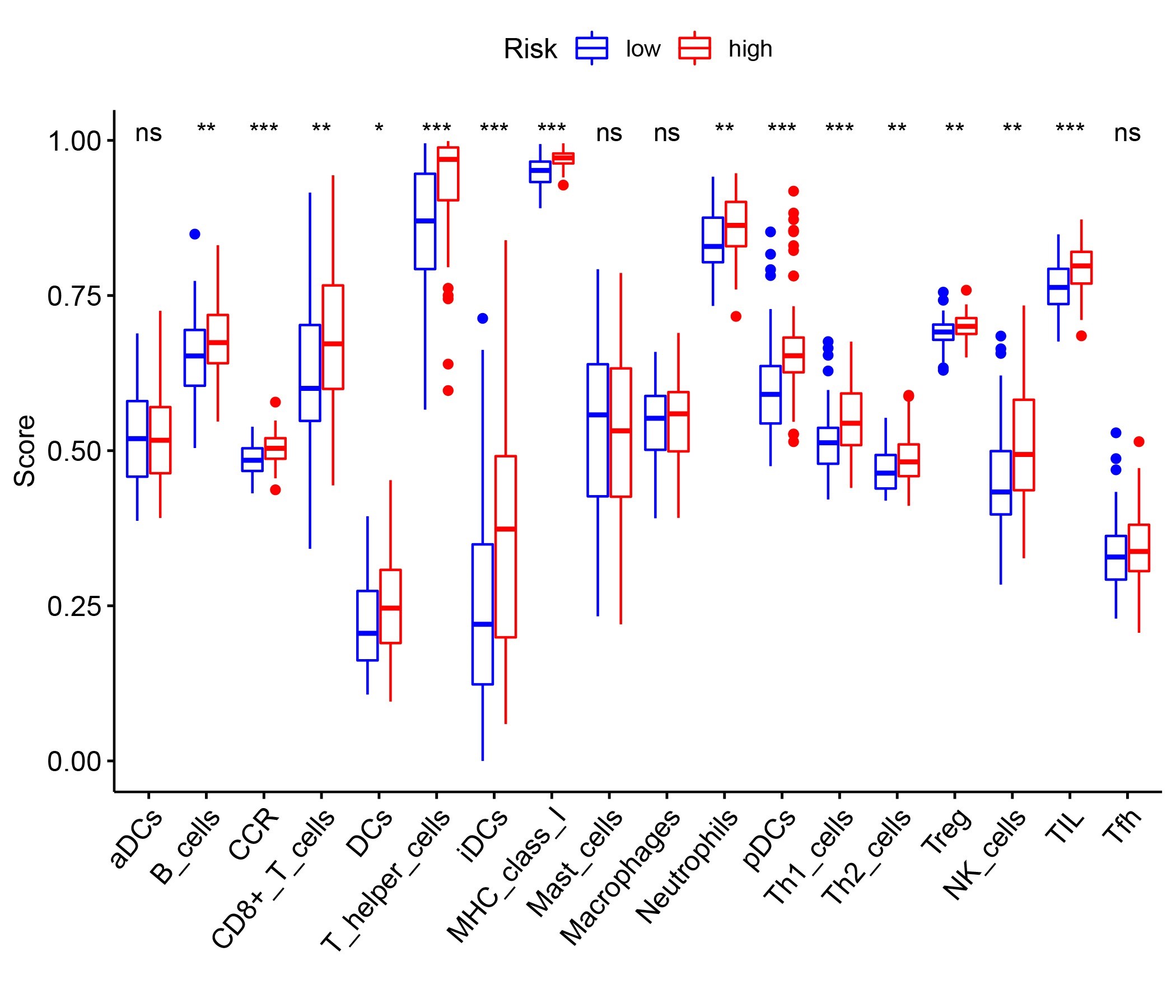

Supplement: Supplementary file 1 [file DataSheet1.zip › Supplementary Material/FIGURE/FIGURE 6/FIGURE 6H_.immCell.jpg]

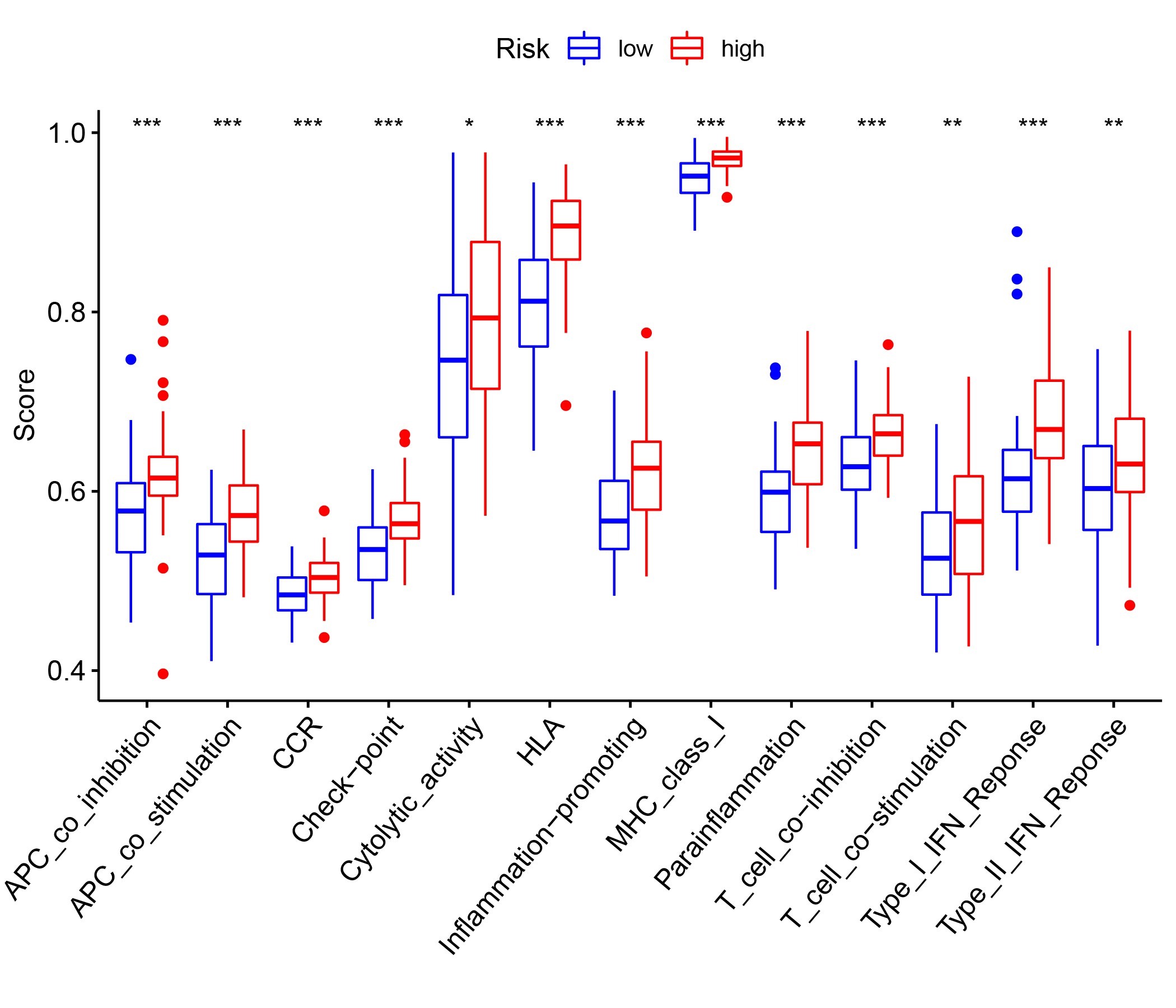

Supplement: Supplementary file 1 [file DataSheet1.zip › Supplementary Material/FIGURE/FIGURE 6/FIGURE 6I_.immFunction.jpg]

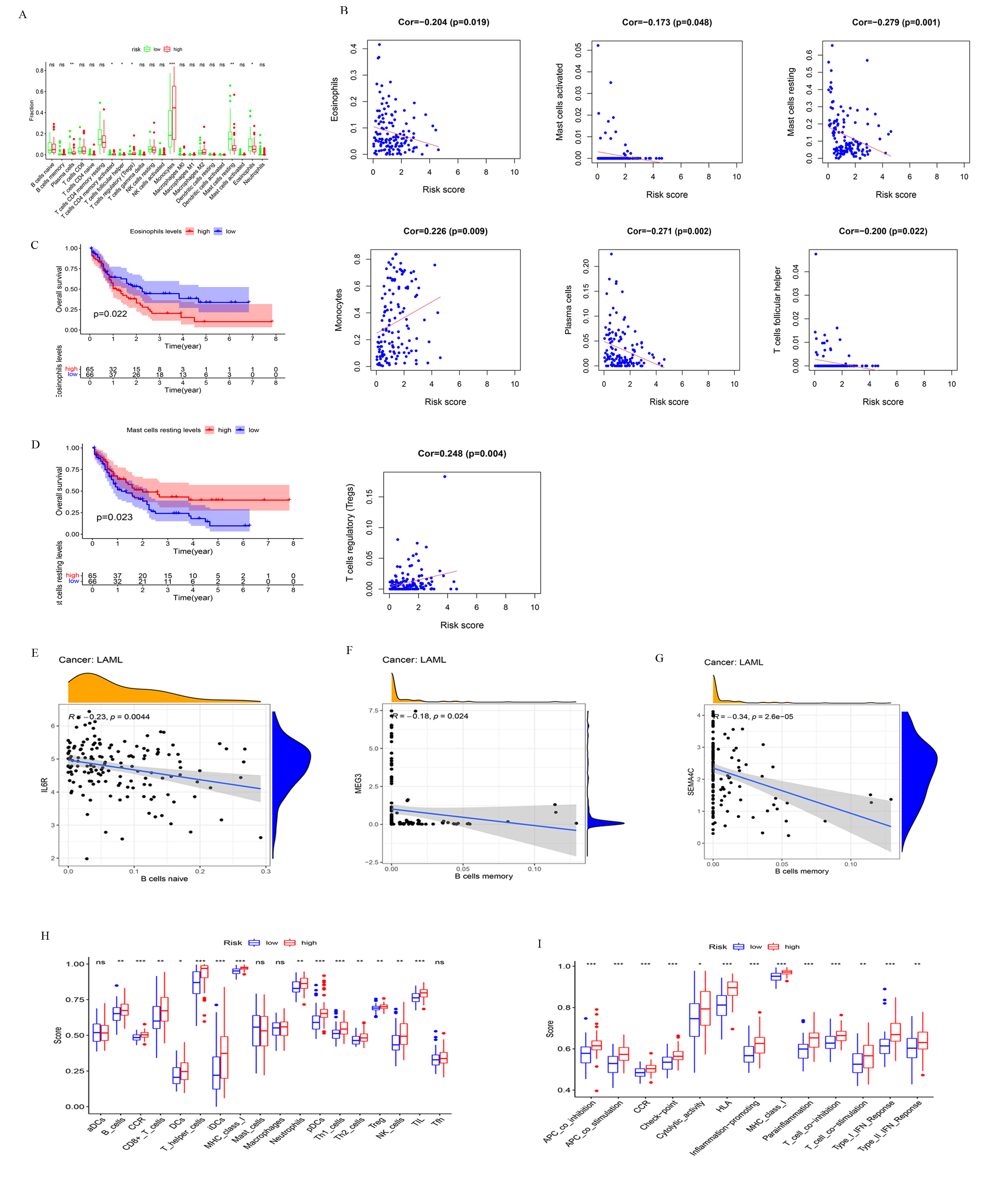

Supplement: Supplementary file 1 [file DataSheet1.zip › Supplementary Material/FIGURE/FIGURE 6.jpg]

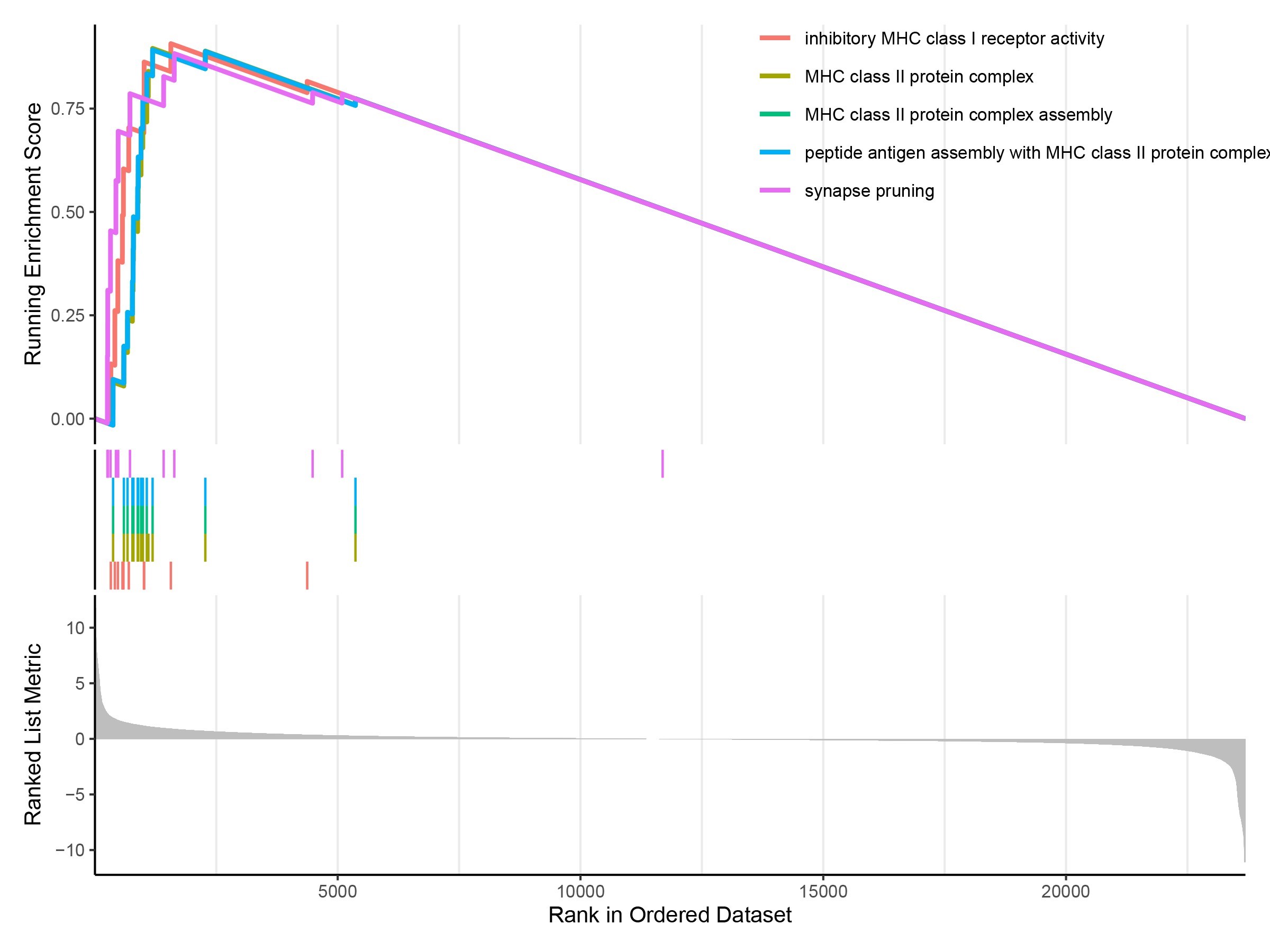

Supplement: Supplementary file 1 [file DataSheet1.zip › Supplementary Material/FIGURE/FIGURE 7/FIGURE 7A_gseaGO.jpg]

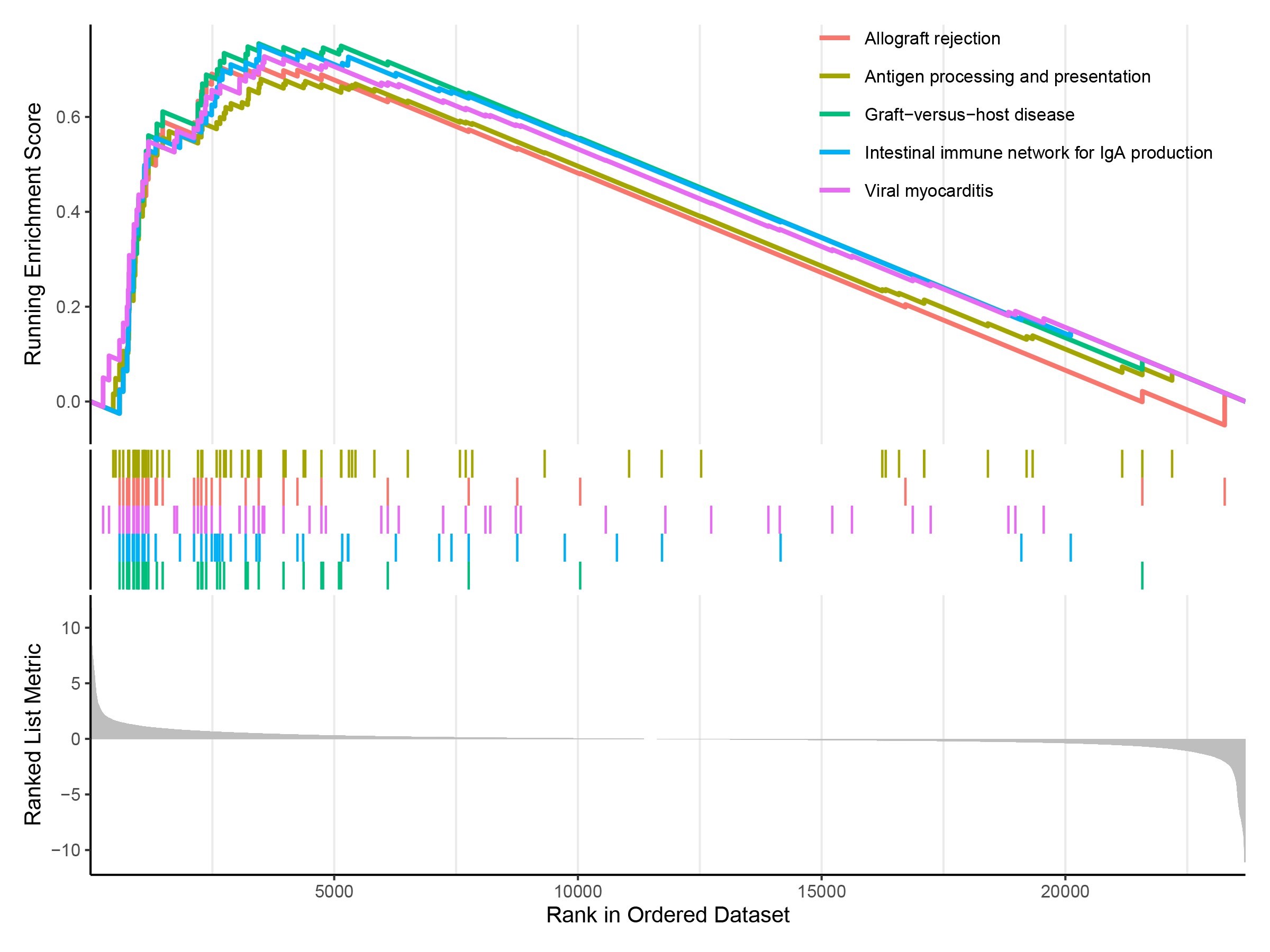

Supplement: Supplementary file 1 [file DataSheet1.zip › Supplementary Material/FIGURE/FIGURE 7/FIGURE 7B_gseaKEGG.jpg]

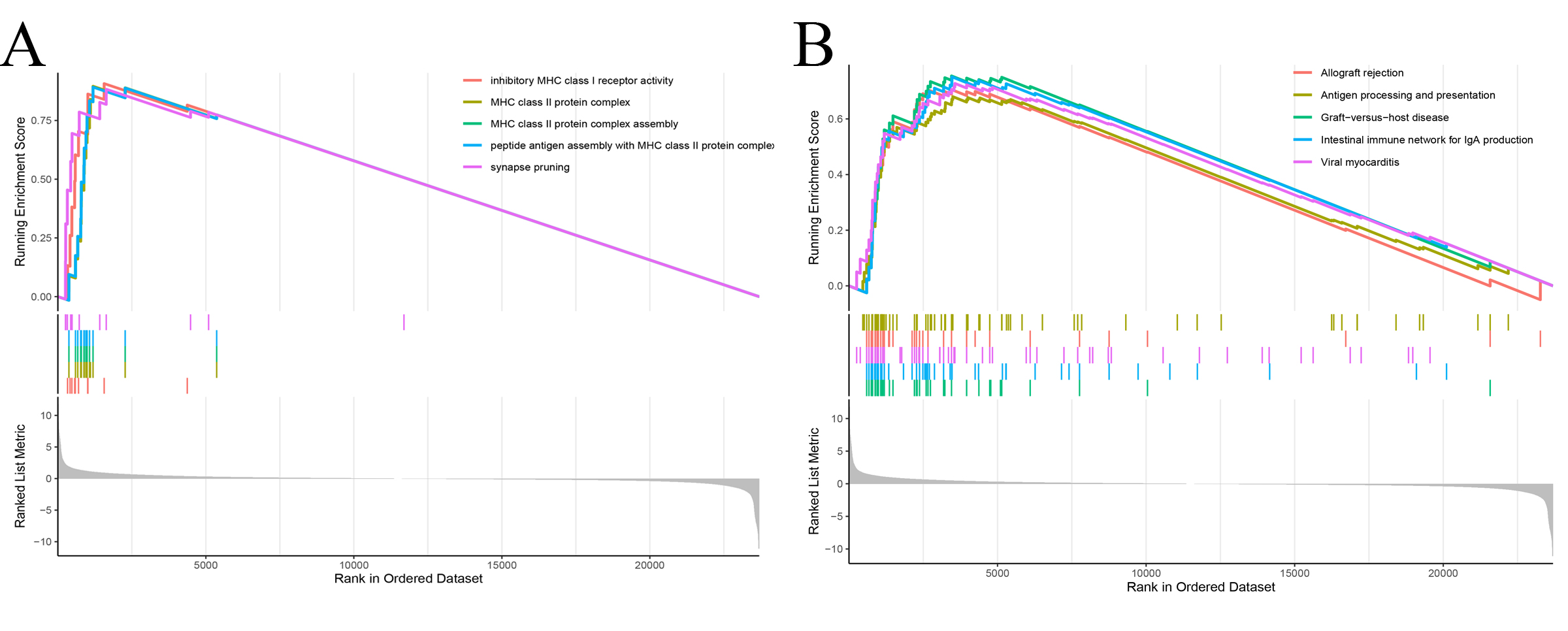

Supplement: Supplementary file 1 [file DataSheet1.zip › Supplementary Material/FIGURE/FIGURE 7.jpg]

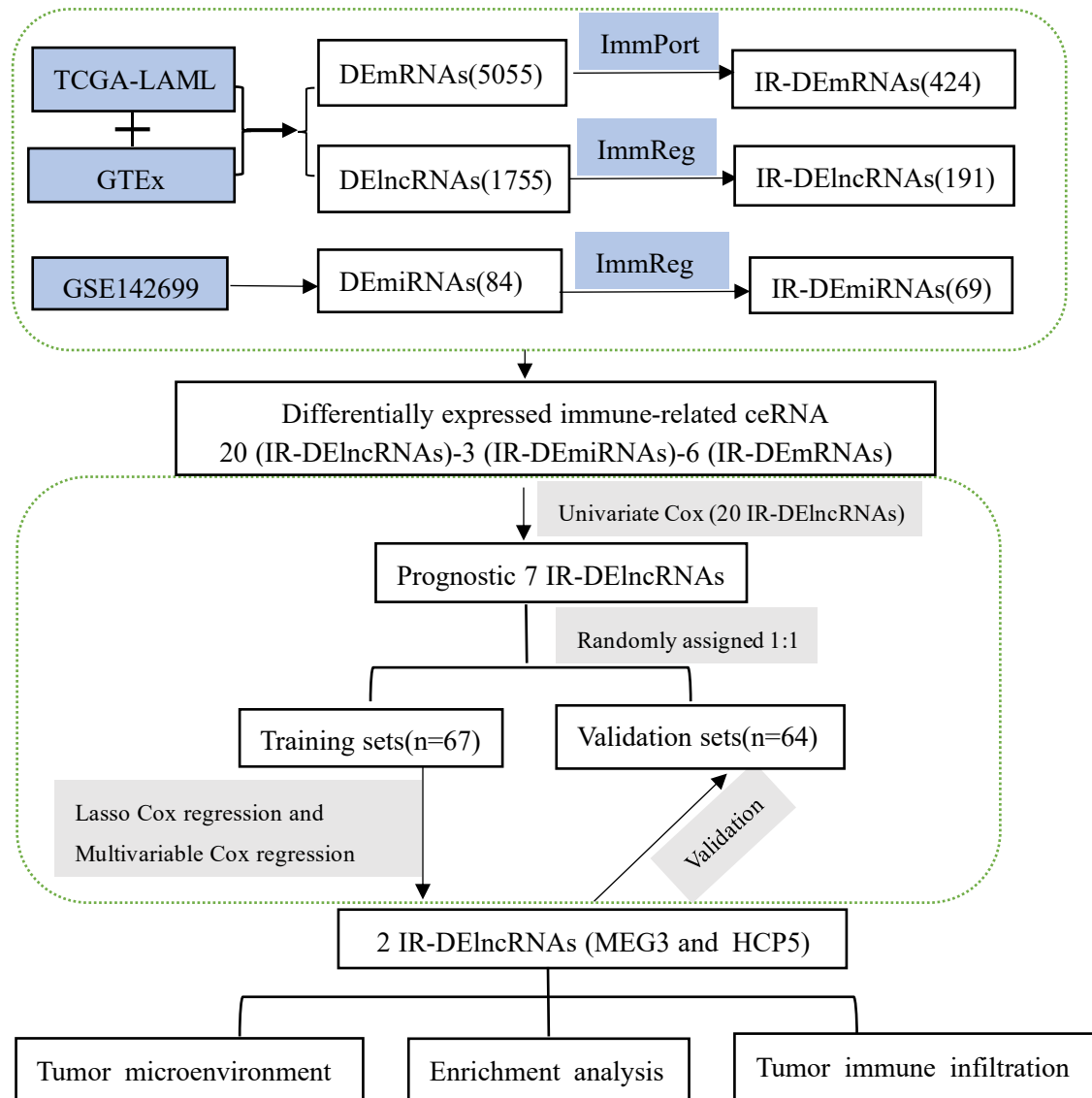

Supplement: Supplementary file 1 [file DataSheet1.zip › Supplementary Material/FIGURE 1.pdf]

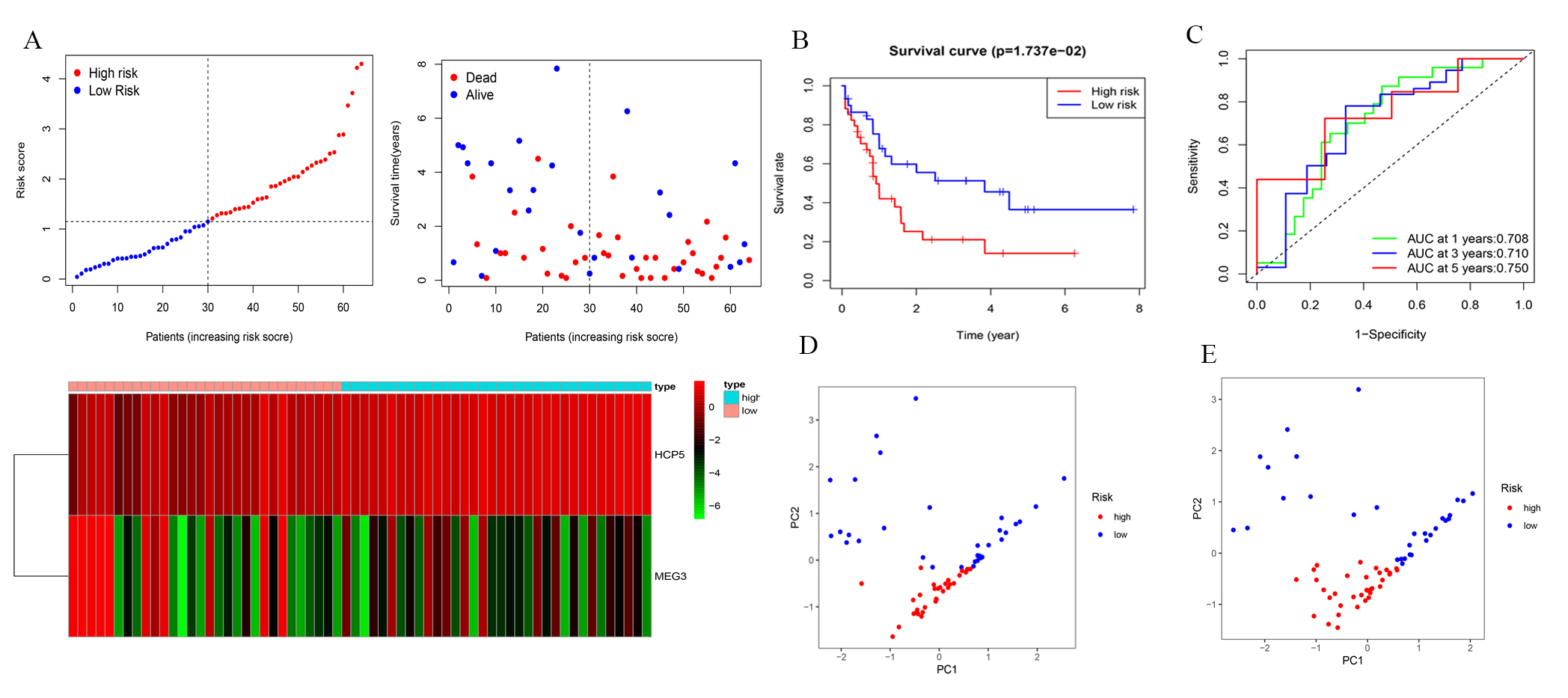

Supplement: Supplementary file 1 [file DataSheet1.zip › Supplementary Material/Supplementary Figure 1/Supplementary Figure 1.jpg]

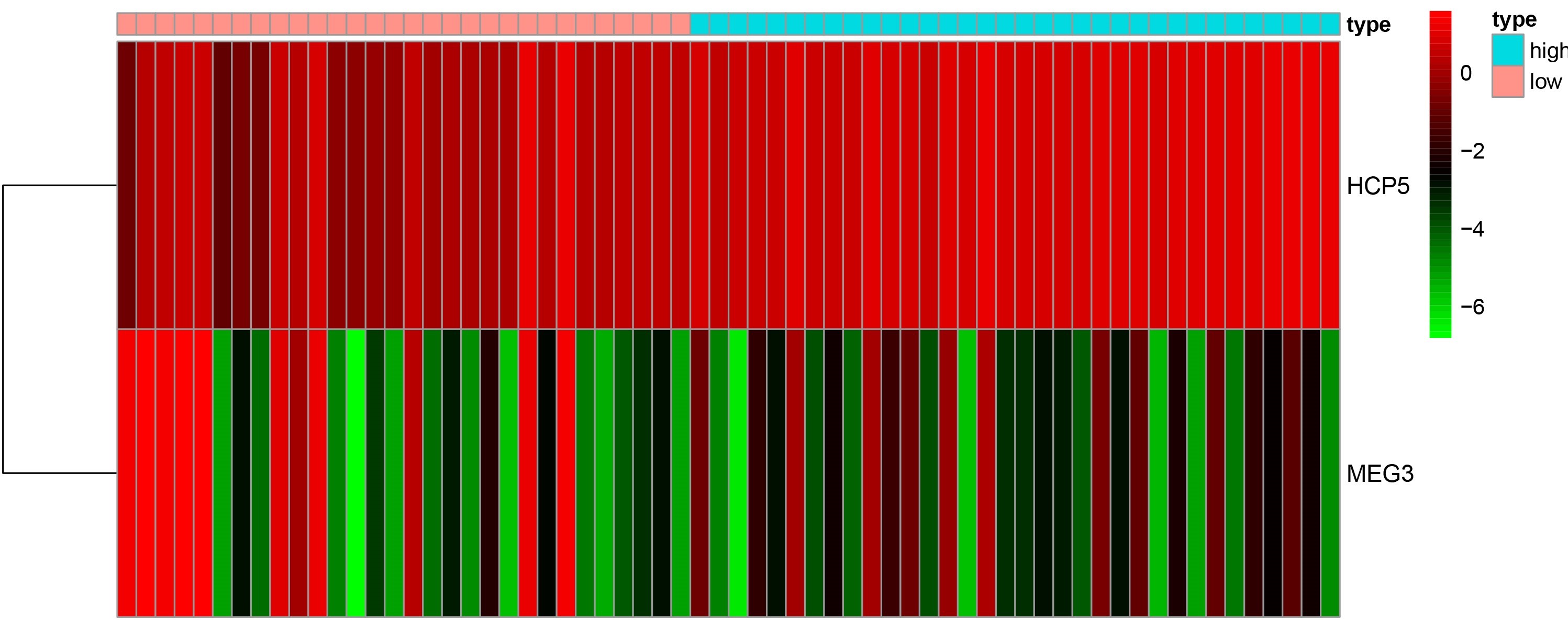

Supplement: Supplementary file 1 [file DataSheet1.zip › Supplementary Material/Supplementary Figure 1/Supplementary Figure 1A_riskTestheatmap.jpg]

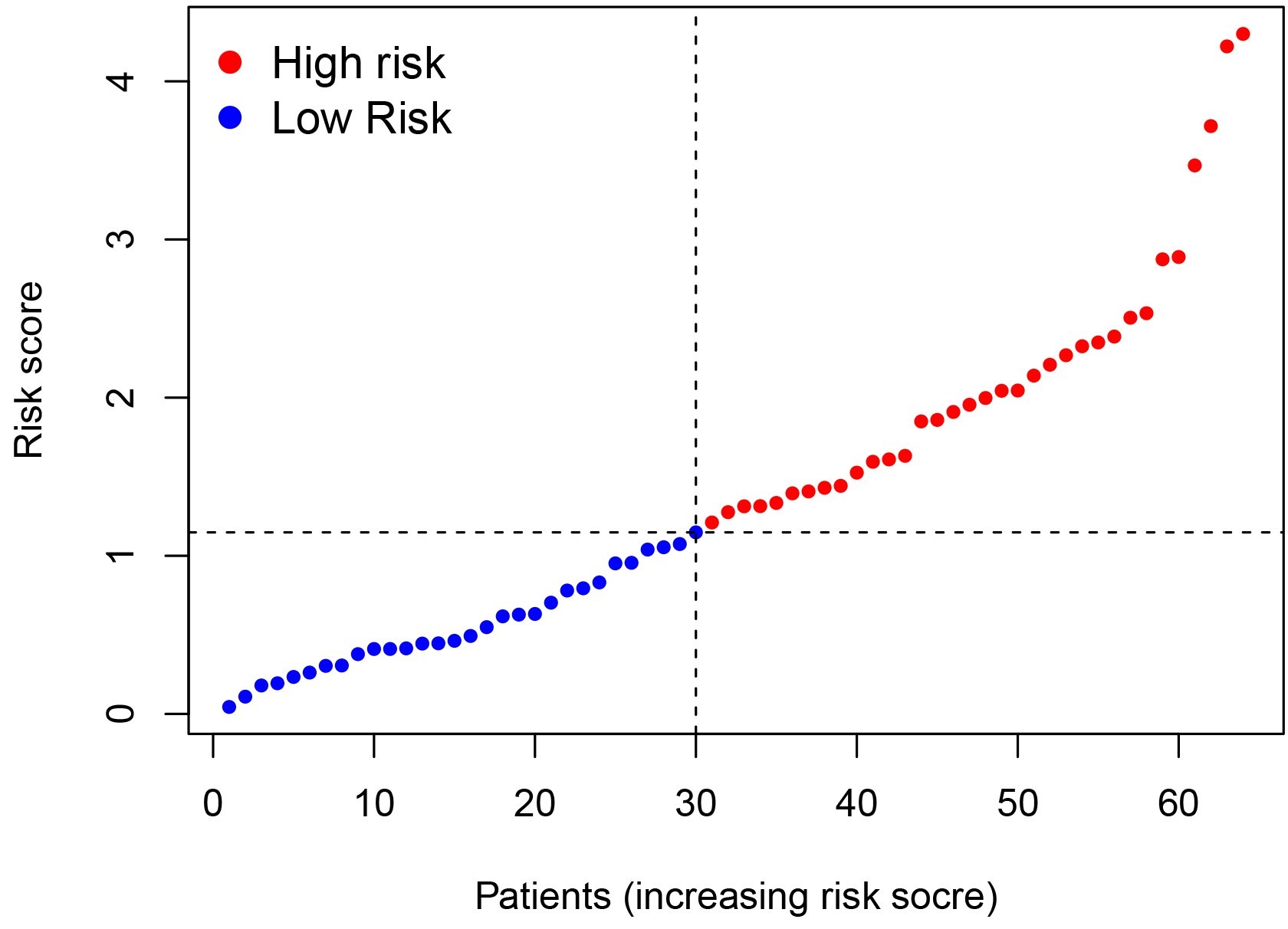

Supplement: Supplementary file 1 [file DataSheet1.zip › Supplementary Material/Supplementary Figure 1/Supplementary Figure 1A_test.riskScore.jpg]

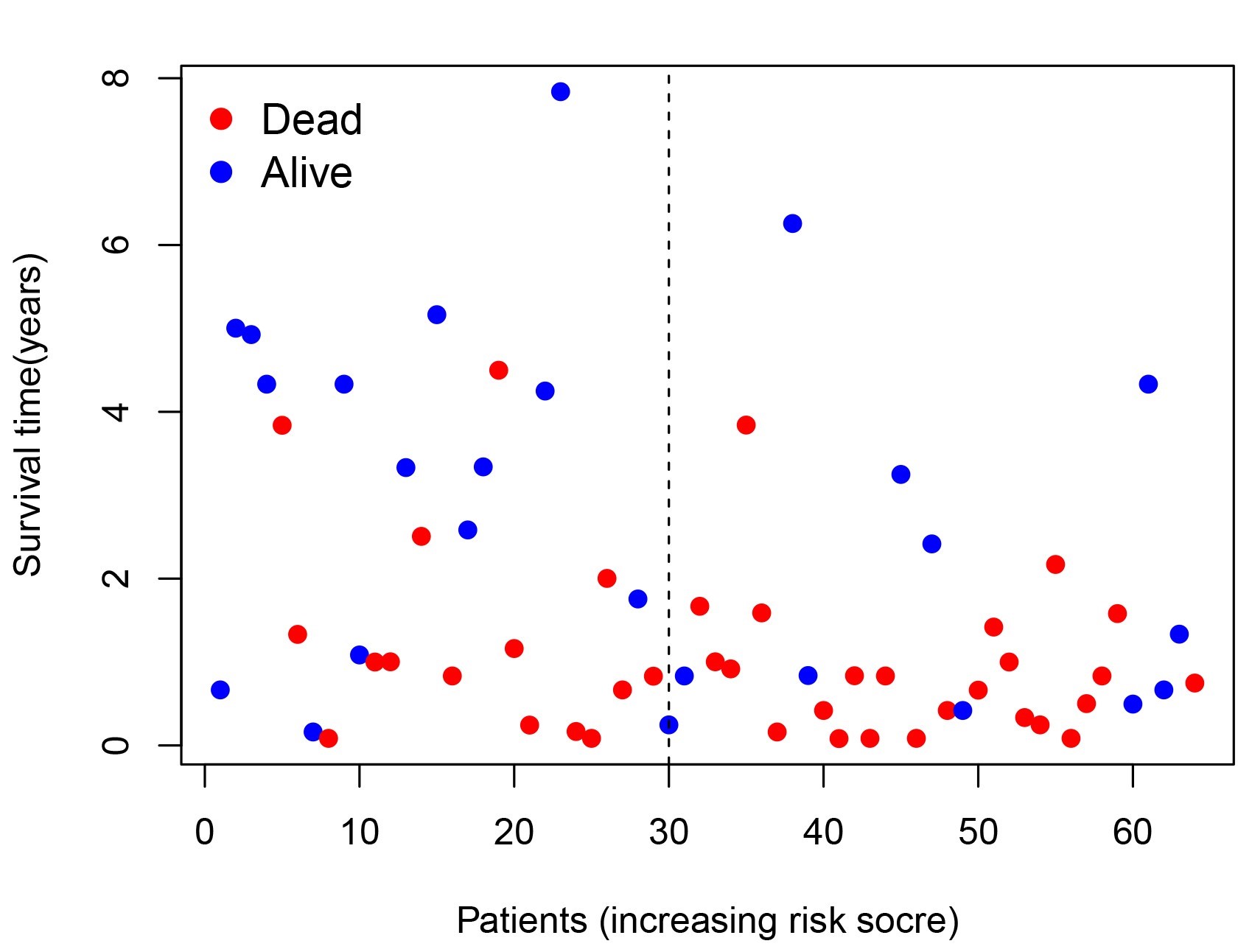

Supplement: Supplementary file 1 [file DataSheet1.zip › Supplementary Material/Supplementary Figure 1/Supplementary Figure 1A_test.survStat.jpg]

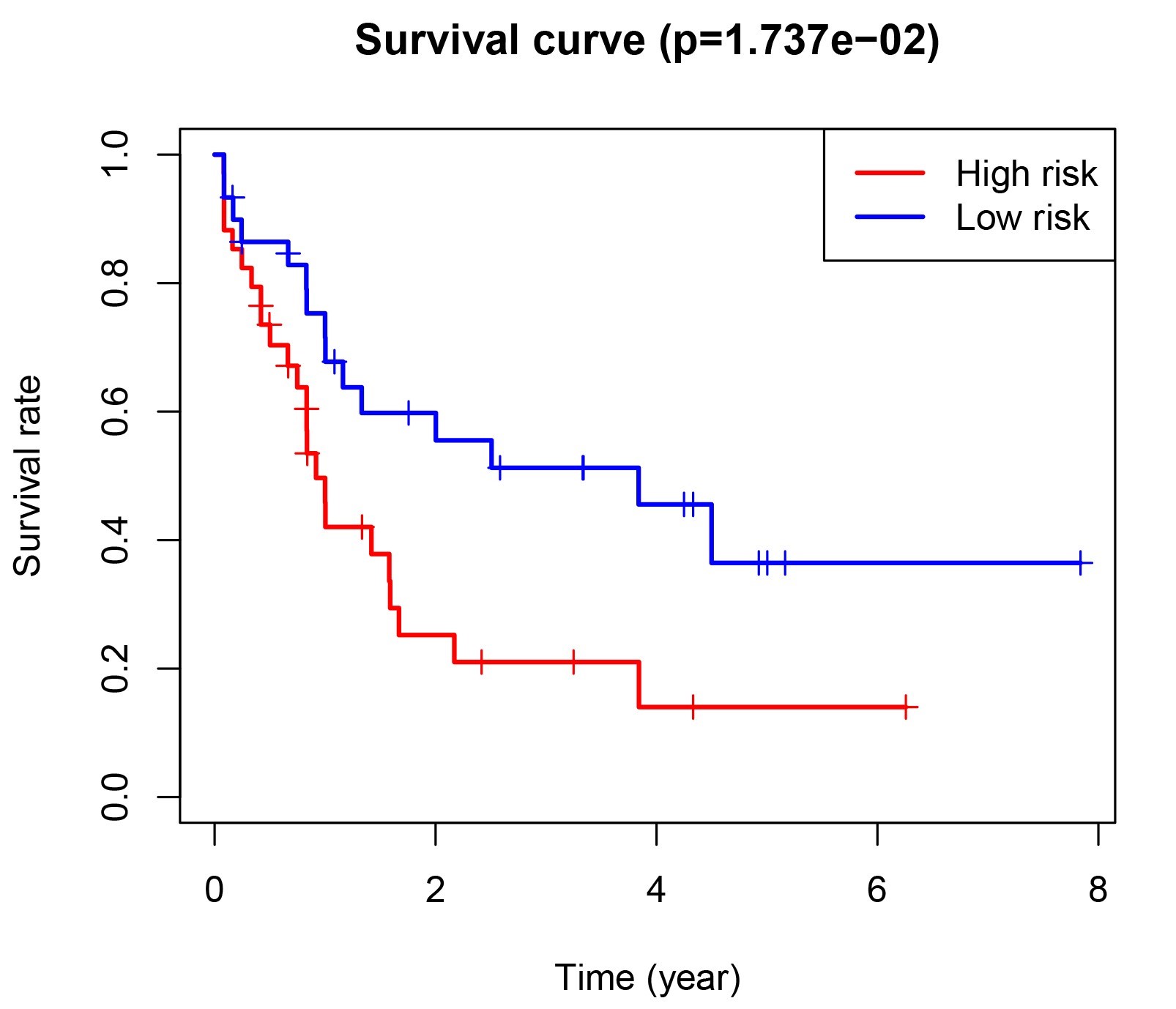

Supplement: Supplementary file 1 [file DataSheet1.zip › Supplementary Material/Supplementary Figure 1/Supplementary Figure 1B_survivalTest.jpg]

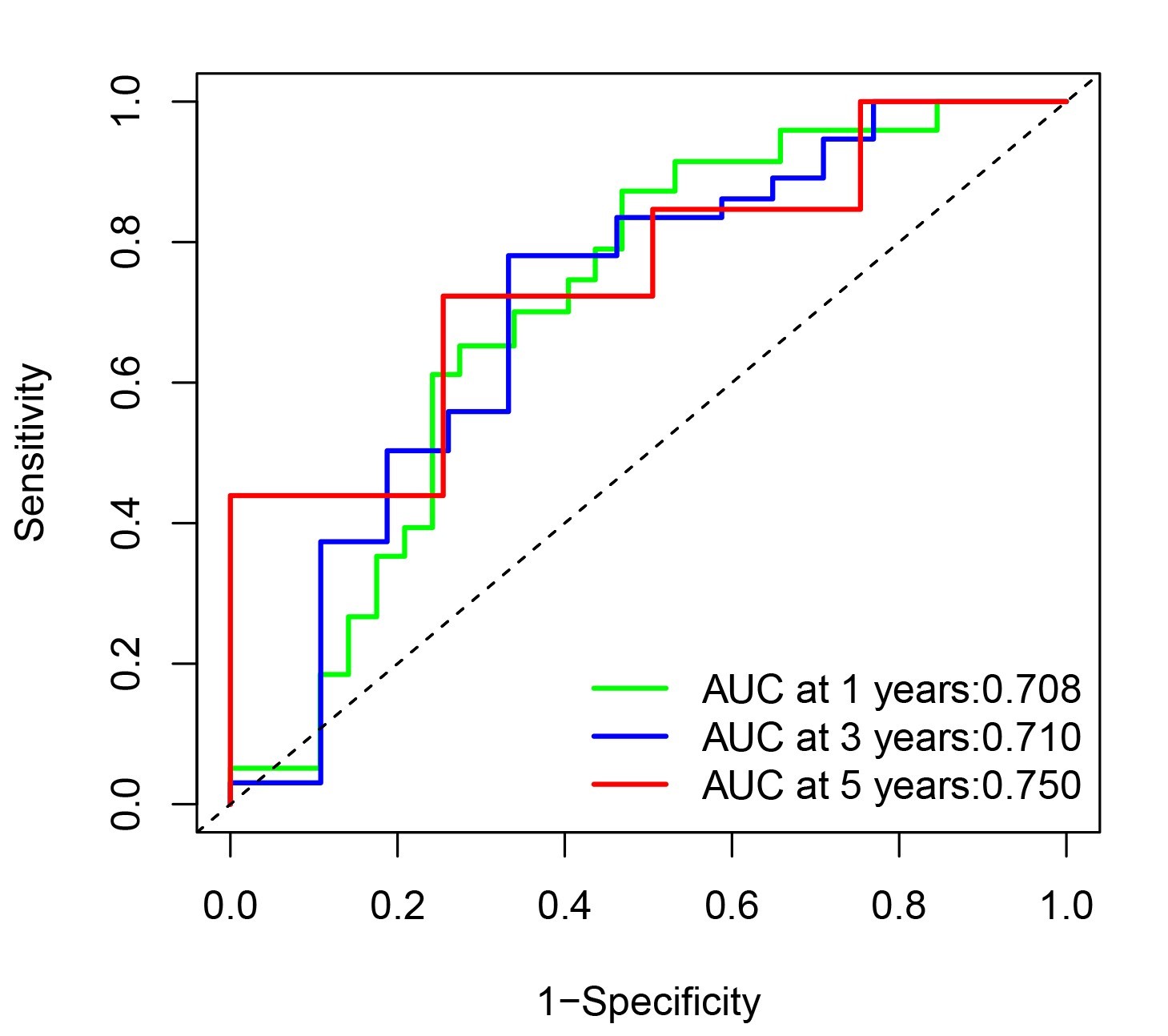

Supplement: Supplementary file 1 [file DataSheet1.zip › Supplementary Material/Supplementary Figure 1/Supplementary Figure 1C_test.ROC.jpg]

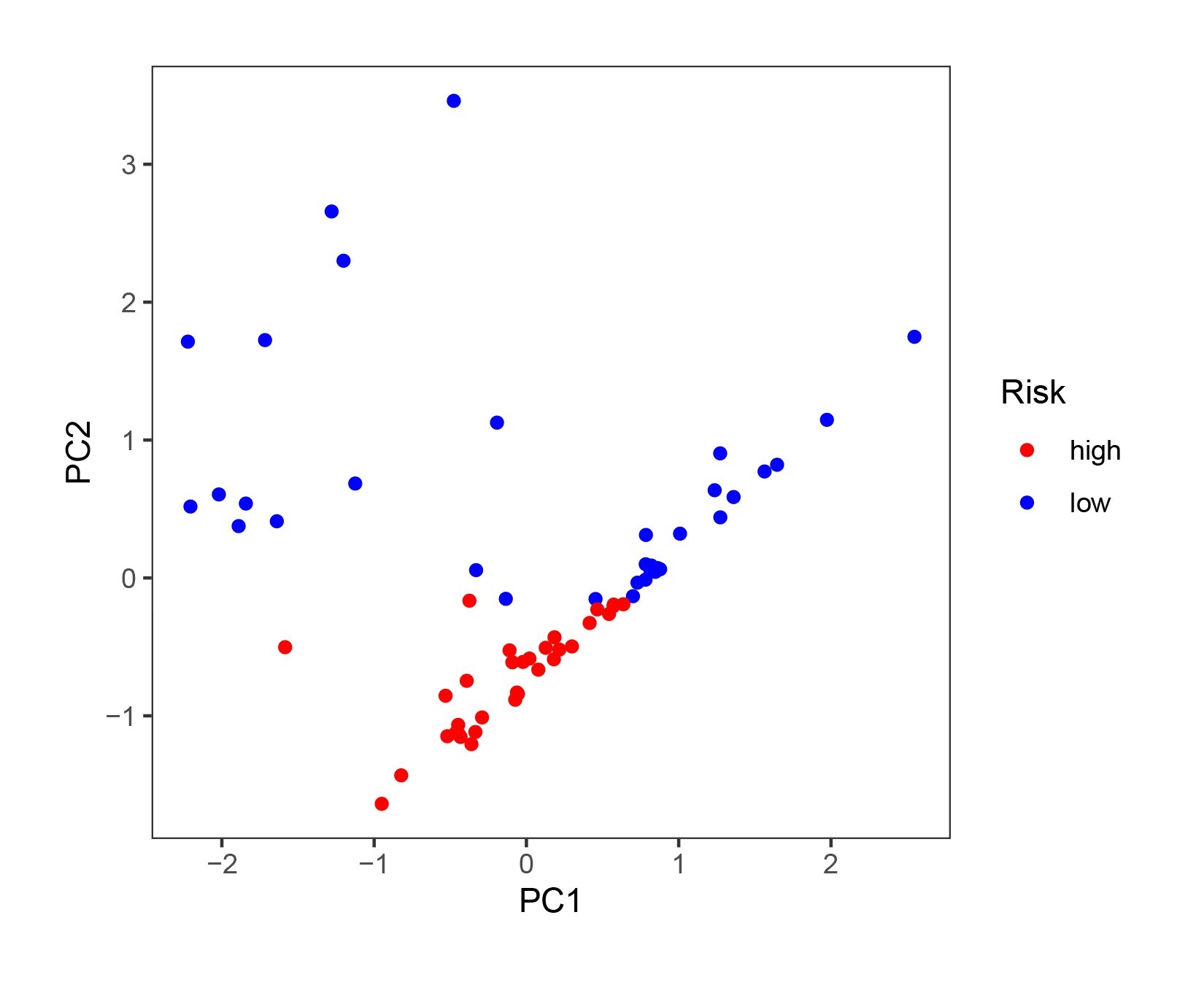

Supplement: Supplementary file 1 [file DataSheet1.zip › Supplementary Material/Supplementary Figure 1/Supplementary Figure 1D_train.PCA.jpg]

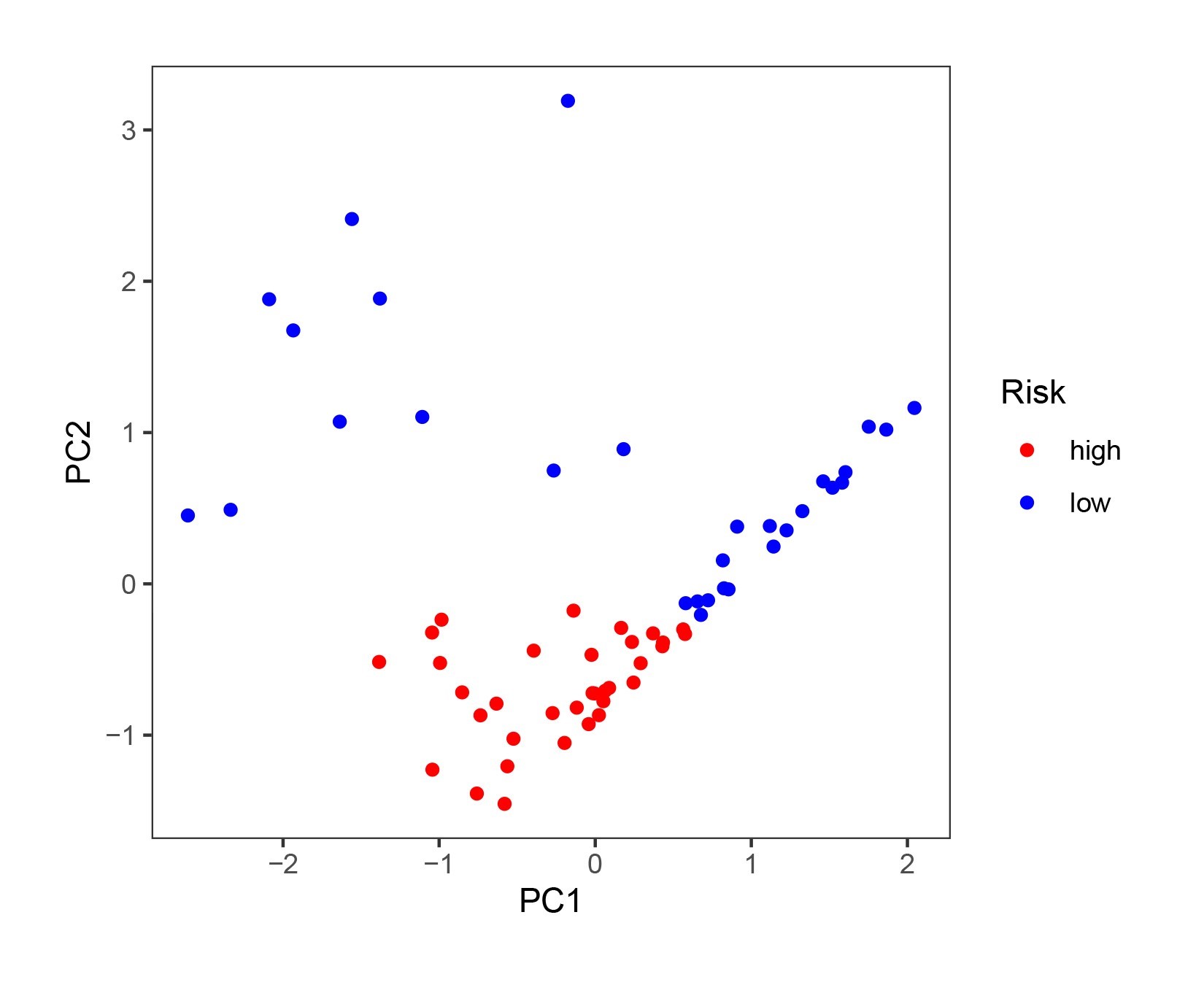

Supplement: Supplementary file 1 [file DataSheet1.zip › Supplementary Material/Supplementary Figure 1/Supplementary Figure 1E_test.PCA.jpg]

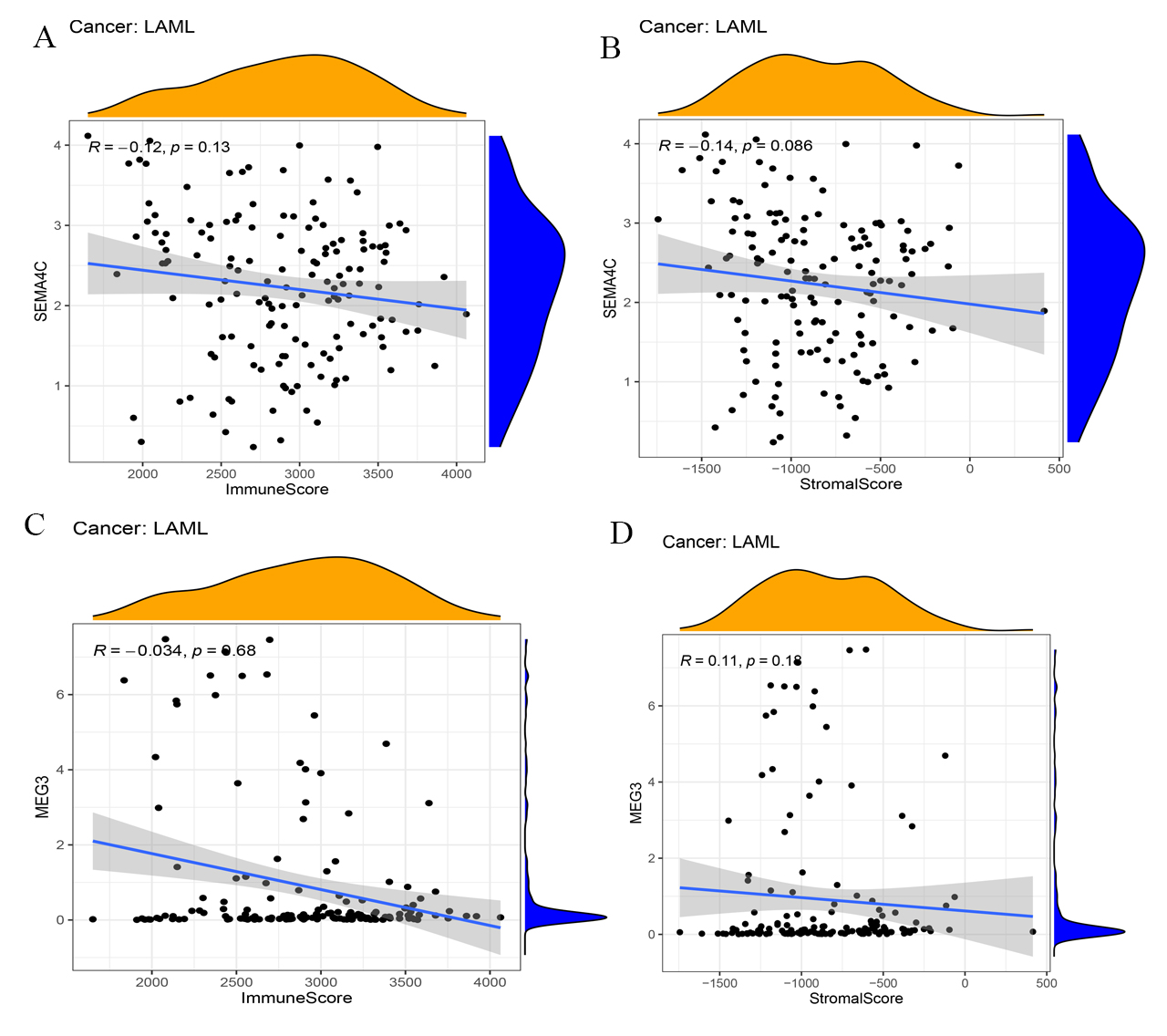

Supplement: Supplementary file 1 [file DataSheet1.zip › Supplementary Material/Supplementary Figure 2/Supplementary Figure 2.jpg]

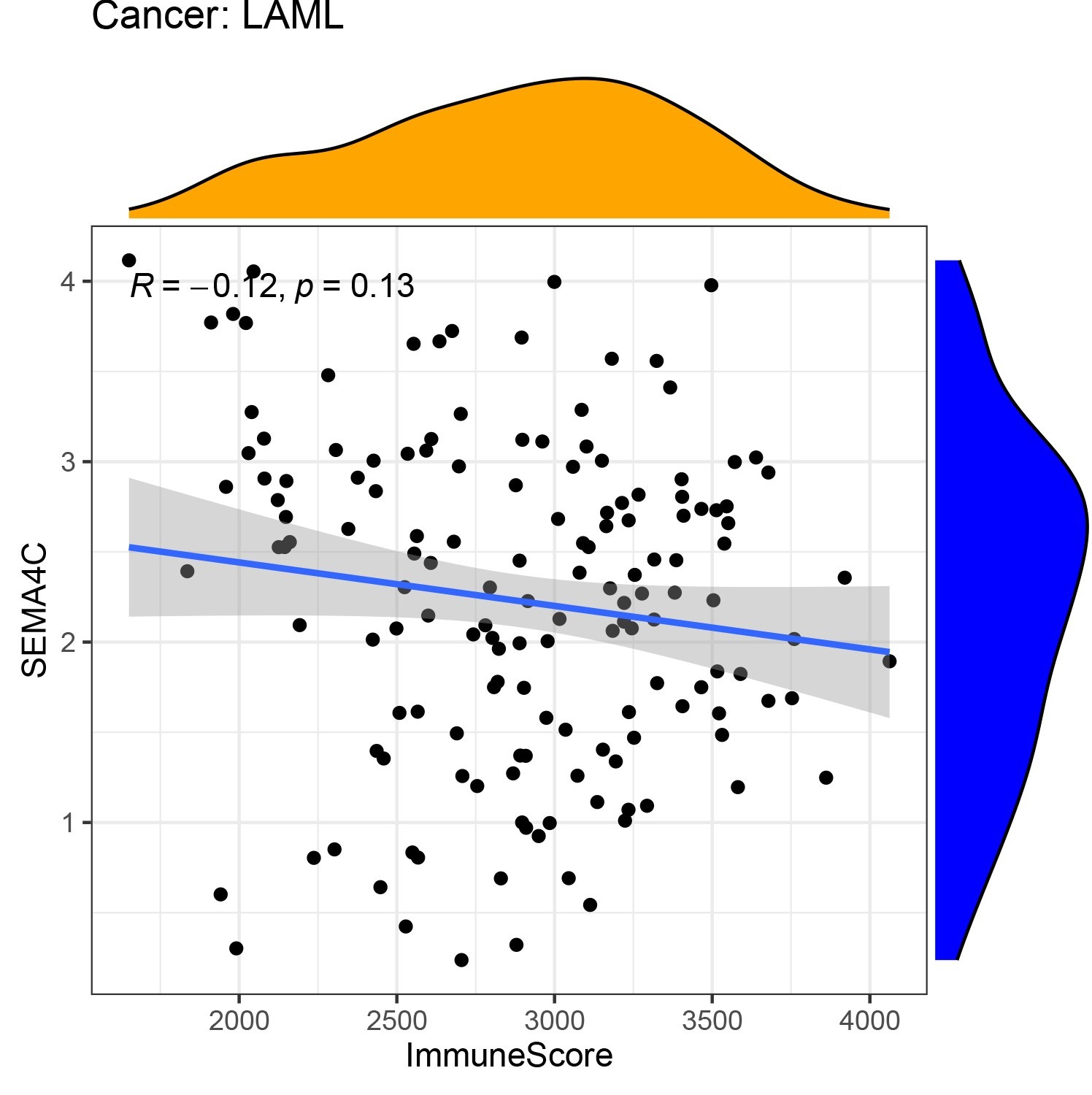

Supplement: Supplementary file 1 [file DataSheet1.zip › Supplementary Material/Supplementary Figure 2/Supplementary Figure 2A_estimateCor.SEMA4C_ImmuneScore.jpg]

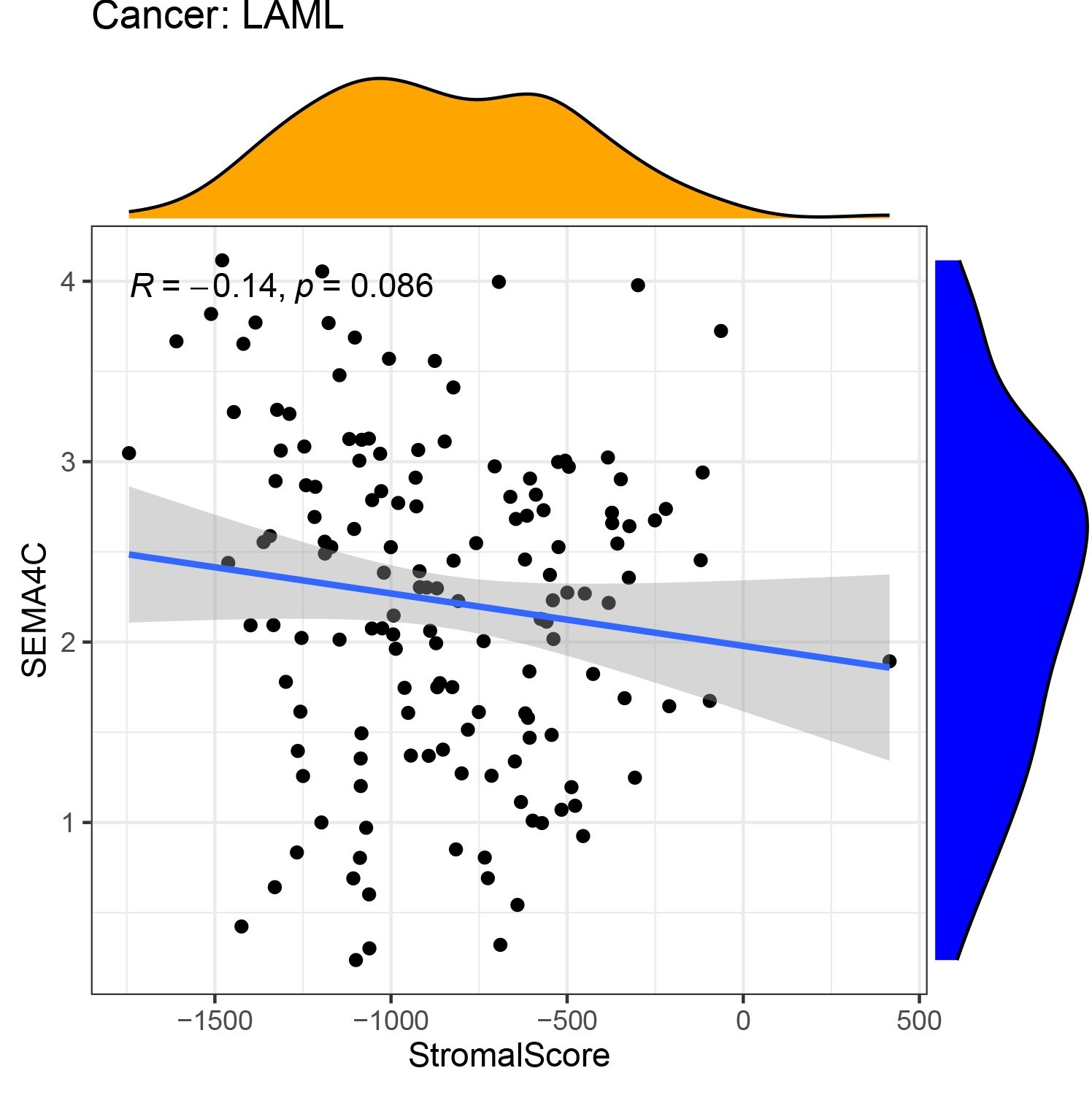

Supplement: Supplementary file 1 [file DataSheet1.zip › Supplementary Material/Supplementary Figure 2/Supplementary Figure 2B_estimateCor.SEMA4C_StromalScore.jpg]

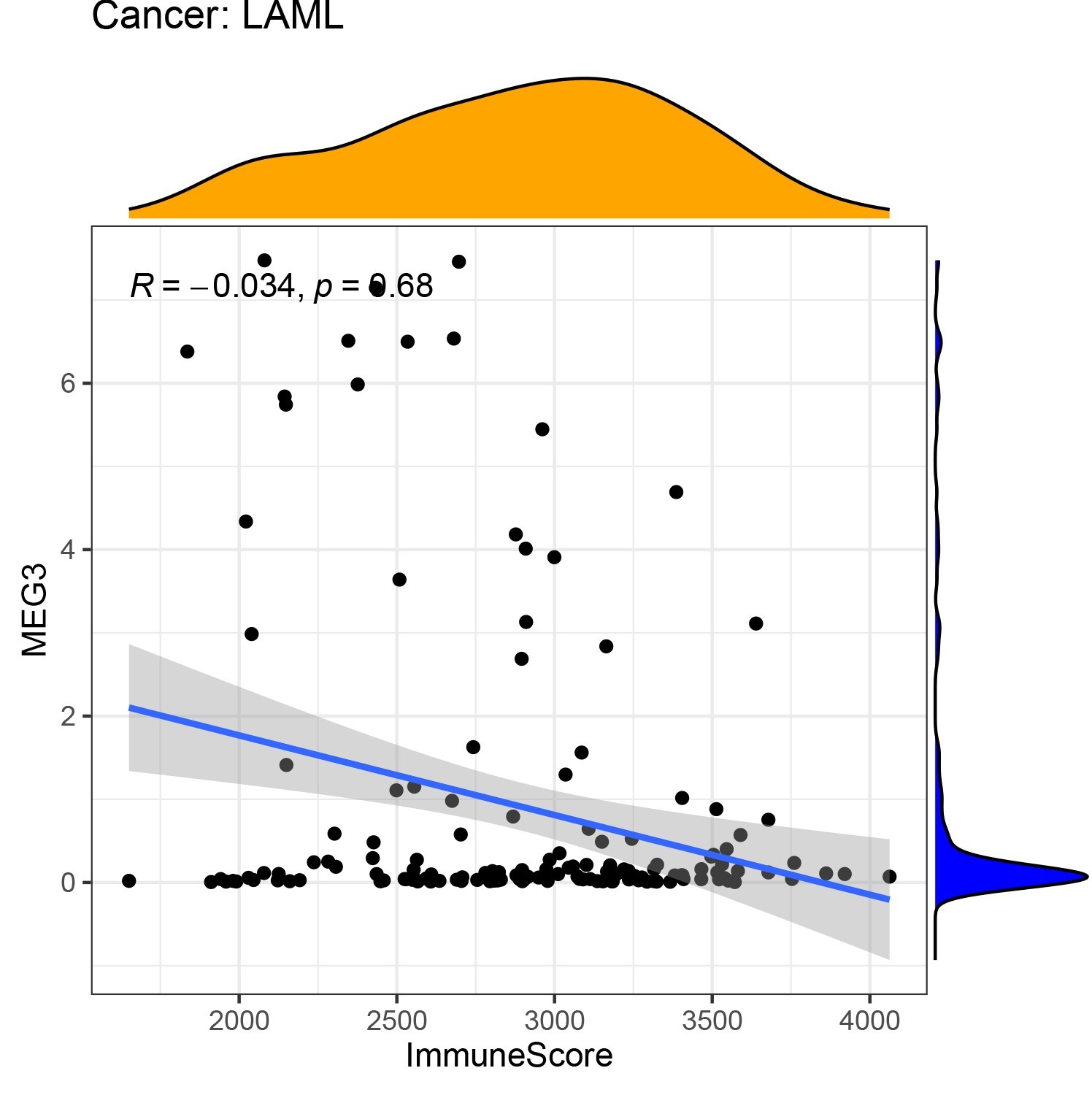

Supplement: Supplementary file 1 [file DataSheet1.zip › Supplementary Material/Supplementary Figure 2/Supplementary Figure 2C_estimateCor.MEG3_ImmuneScore.jpg]

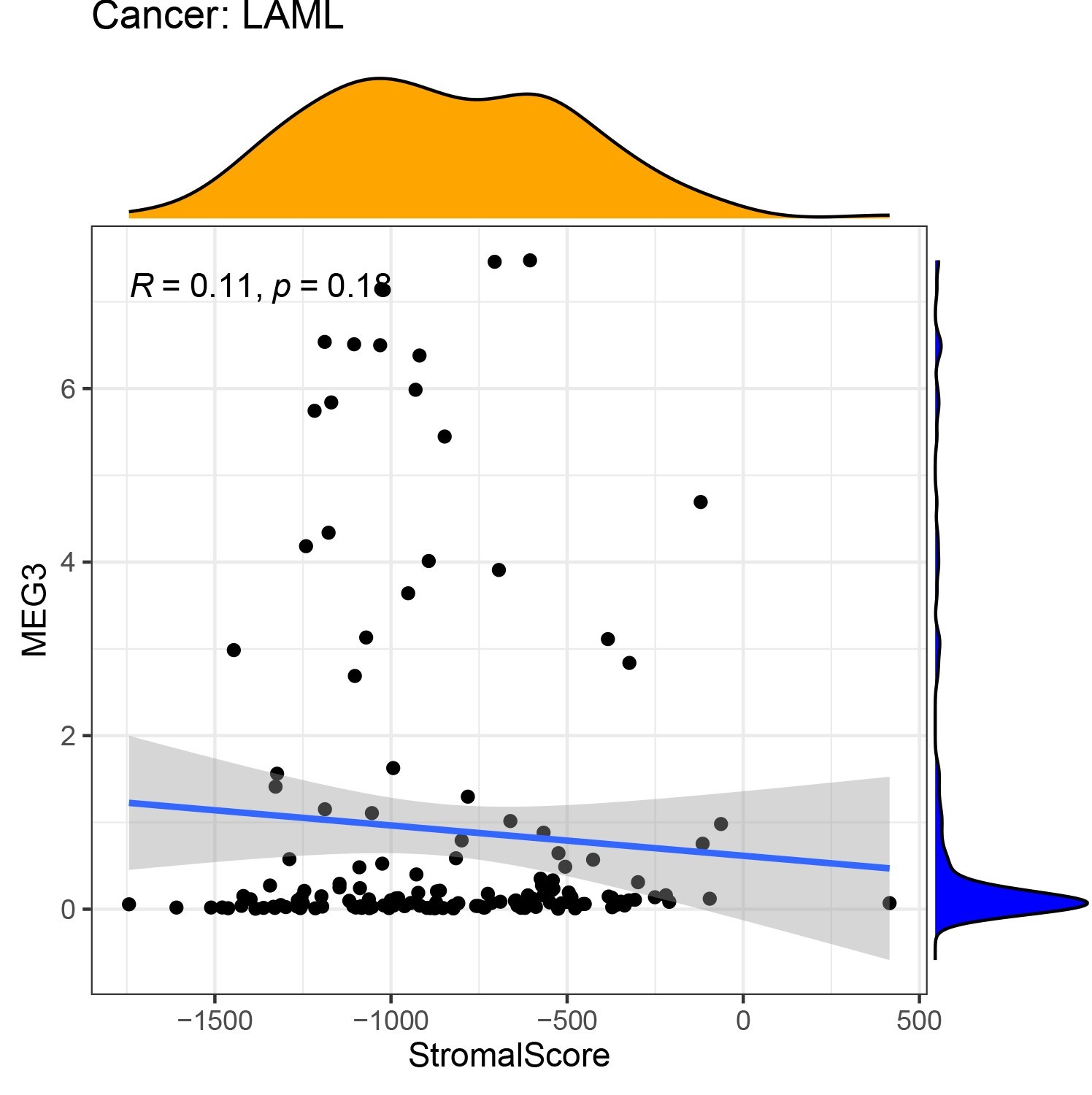

Supplement: Supplementary file 1 [file DataSheet1.zip › Supplementary Material/Supplementary Figure 2/Supplementary Figure 2D_estimateCor.MEG3_StromalScore.jpg]
